# Effect of Varying Nitrate Concentrations on Denitrifying Phosphorus Uptake by DPAOs With a Molecular Insight Into Pho Regulon Gene Expression

**DOI:** 10.3389/fmicb.2019.02586

**Published:** 2019-11-08

**Authors:** Chandan Mukherjee, Rajojit Chowdhury, Mst. Momtaj Begam, Sayak Ganguli, Ritabrata Basak, Basab Chaudhuri, Krishna Ray

**Affiliations:** ^1^Environmental Biotechnology Group, Department of Botany, West Bengal State University, Kolkata, India; ^2^Theoretical and Computational Biology Division, AIIST and The Biome, Kolkata, India; ^3^Department of Biochemistry, Ballygunge Science College, University of Calcutta, Kolkata, India; ^4^West Bengal State University, Kolkata, India

**Keywords:** denitrifying phosphorus removal, DPAO, single stage anoxic reactor, Pho regulon, negative regulation, transcriptional repression, poly-phosphate accumulation

## Abstract

Bacterial Pho regulon is a key regulator component in biological phosphorus-uptake. Poly-phosphate accumulating bacteria used in enhanced biological phosphorus removal (EBPR) system encounter negative regulation of the Pho regulon, resulting in reduced phosphorus-uptake from phosphorus-replete waste effluents. This study demonstrates possible trends of overcoming the PhoU negative regulation, resulting in excessive PO_4_^3–^-P uptake at varying concentrations of NO_3_^–^-N through denitrifying phosphorus removal process. We investigated the Pho regulon gene expression pattern and kinetic studies of P-removal by denitrifying phosphate accumulating organisms (DPAOs) which are able to remove both PO_4_^3–^-P and NO_3_^–^-N in single anoxic stage with the utilization of external carbon sources, without the use of stored polyhydroxyalkanoate (PHA) and without any anaerobic-aerobic or anaerobic-anoxic switches. Our study establishes that a minimum addition of 100 ppm NO_3_^–^-N leads to the withdrawal of the negative regulation of Pho regulon and results in ∼100% P-removal with concomitant escalated poly-phosphate accumulation by our established DPAO isolates and their artificially made consortium, isolated from sludge sample of PO_4_^3–^ -rich parboiled rice mill effluent, in a settling tank within 12 h of treatment. The same results were obtained when a phosphate rich effluent (stillage from distillery) mixed with a nitrate rich effluent (from explosive industry) was treated together in a single phase anoxic batch reactor, eliminating the need for alternating anaerobic/aerobic or anaerobic/anoxic switches for removing both the pollutants simultaneously. The highest poly-phosphate accumulation was observed to be more than 17% of cell dry weight. Our studies unequivocally establish that nitrate induction of Pho regulon is parallely associated with the repression of *PhoU* gene transcription, which is the negative regulator of Pho regulon. Based on earlier observations where similar nitrate mediated transcriptional repression was cited, we hypothesize the possible involvement of NarL/NarP transcriptional regulator proteins in *PhoU* repression. At present, we propose this denitrifying phosphorus removal endeavor as an innovative methodology to overcome the negative regulation of Pho regulon for accelerated unhindered phosphorus remediation from phosphate rich wastewater in India and the developing world where the stringency of EBPR and other reactors prevent their use due to financial reasons.

## Introduction

Phosphorus (P) rich waste effluents are objects of great concern worldwide. Globally, these waste effluents are treated with an enhanced biological phosphorus removal (EBPR) system that is based on an activated sludge process involving a group of microorganisms called poly-phosphate-accumulating organisms (PAOs), which actively take up soluble phosphorus (PO_4_^3–^-P) from wastewater and accumulate it in the form of poly-phosphate (poly-P) granules. This PO_4_^3–^-P uptake is enhanced when the organisms are alternated between a carbon-rich anaerobic environment and a carbon-poor aerobic environment. EBPR has the potential to remove 80–90% PO_4_^3–^-P, with a residual PO_4_^3–^-P value as low as <1 ppm in the effluent. Minnesota Pollution Control Agency (2006) reported an efficient EBPR system followed by good final clarification which achieved effluent phosphorus concentrations of 0.7 ppm if sufficient VFAs are available in the process. Lower effluent concentrations even down to 0.1 ppm could be achieved through supplemental chemical treatment and advanced effluent filtration techniques applied after EPBR operation. United States Environmental Protection Agency (2007) reported that Biological Nutrient Removal if complemented with chemical addition, tertiary clarification and filtration process, it can achieve average effluent phosphorus concentration as below as 0.06–0.07 ppm. Although a study from China reported 93% PO_4_^3–^-P removal efficiency by the EBPR process with effluent PO_4_^3–^-P level as low as 0.253 ppm from municipal wastewater, the influent PO_4_^3–^-P level was only 3.613 mg L^–1^ (Li et al., 2011). Similarly, another study on municipal wastewater treatment plants of China reported that 95% P removal was achieved in EBPR, but the influent P conc. was between 3 and 8.7 mg L^–1^ (Yang et al., 2017).

Phosphate-accumulating organisms usually face a genetic regulatory control that limits their PO_4_^3–^-P uptake ability. Through a coordinated expression of genes under the Pho regulon, the poly-phosphate-accumulating bacteria sense the external PO_4_^3–^-P conc., and external low levels allow the cells to uptake PO_4_^3–^-P, while the reverse takes place under externally PO_4_^3–^-P replete conc. The Pho regulon is a global regulatory mechanism that is involved in bacterial PO_4_^3–^-P management and was first characterized in *Escherichia coli* (*E. coli*) (Wanner and Chang, 1987). In *E. coli*, the Pho regulon expresses five major proteins, apart from a two-component system, PhoR-PhoB. Four of the major proteins are phosphate transporters (Pst), namely, PstS, PstC, PstA and PstB, and are the most conserved members of the Pho regulon system in bacteria, while the fifth is a metal-binding protein, PhoU. Under external PO_4_^3–^-P deplete conditions, PhoB is activated by PhoR acting as a kinase, but under external PO_4_^3–^-P replete conditions, PhoB activation is interrupted by PhoR acting as a phosphatase, which causes repression of the phosphate regulon (Yamada et al., 1990; Muda et al., 1992; Carmany et al., 2003). PhoU is involved in PhoB dephosphorylation under PO_4_^3–^-P rich conditions in a yet unknown mechanism. When PhoU is mutated or deleted, PhoR behaves as a constitutive kinase to phosphorylate PhoB, which leads to a high expression of Pho regulon genes (Steed and Wanner, 1993; Rice et al., 2009). This shows that PhoU is involved in the control of the Pst system and prevents uncontrolled PO_4_^3–^-P uptake, which could be toxic for the cells (Surin et al., 1986; Muda et al., 1992; Carmany et al., 2003; Gardner et al., 2014; Santos-Beneit, 2015). This phenomenon was observed in both *E. coli* and *Synechocystis* sp., which suggested that the *phoU* mutants might contribute to improved biological PO_4_^3–^-P removal and accumulation in the form of poly-P from wastewaters (Morohoshi et al., 2002). *E. coli phoU* mutants exhibited 1000-fold higher levels of poly-P accumulation than that of the wild-type under P-rich conditions, and an introduction of the wild-type *PhoU* gene back into the mutant reinstated poly-P accumulation to the wild-type level, thereby establishing the *PhoU* gene as the negative regulator for both PO_4_^3–^-P removal and poly-P accumulation (Kuroda and Ohtake, 2000). An environmental bacterium, *Citrobacter freundii*, was engineered for overexpression of the polyphosphate kinase gene based on a solo medium-copy plasmid strategy that resulted in 12.7% poly-P accumulation by the cells, thus overcoming the negative regulation of PhoU (Wang et al., 2017). The negative regulation of PhoU is a major limitation to PO_4_^3–^-P bioremediation when the soluble PO_4_^3–^-P level is high in waste effluents, e.g., effluents from fertilizer industry (25–308 ppm of PO_4_^3–^-P) (Abou-Elela et al., 1995), parboiled rice mill industry (34–143 ppm of PO_4_^3–^-P) (Faria et al., 2006), and rice-based distilleries (223.5 ± 27.5 ppm of PO_4_^3–^-P) (Bhattacharyya et al., 2014). The screening and development of *phoU* mutants of PAOs was considered to be a solution to overcome this limitation. However, the growth of *phoU* mutants was found to be severely defective and they were easily outgrown by revertant(s) that had lost the ability to accumulate poly-P during growth in a nutrient-rich medium (Hirota et al., 2013).

In the EBPR system, the PAOs are able to store phosphorus through sequential anaerobic-aerobic conditions. Carbon sources, particularly volatile fatty acids (VFAs), are taken up anaerobically and stored as poly-β-hydroxyalkanoates (PHA) with the release of P to the outer media and the degradation of glycogen. A higher amount of P is then taken up from the external media when an electron acceptor is supplied (normally oxygen, i.e., aerobic conditions) through PHA oxidation, which is accompanied by biomass growth and the regeneration of glycogen (Carvalho et al., 2007). Soon it was demonstrated that, not only under aerobic conditions but also under anoxic conditions, i.e., with nitrate as the electron acceptor, some PAOs are capable of phosphate uptake and accumulation (Kuba et al., 1993; Egly and Zehnder, 1994; Jørgensen and Pauli, 1995; Barker and Dold, 1997; Mino et al., 1998; Oehmen et al., 2010). It was suggested that nitrate can be used as an electron acceptor (i.e., anoxic conditions) instead of oxygen, which is advantageous because both NO_3_^–^-N and PO_4_^3–^-P are thus removed in the same process (Kerrn-Jespersen and Henze, 1993; Kuba et al., 1993). These organisms came to be known as denitrifying polyphosphate accumulating organisms or DPAOs. Moreover, when compared to conventional EBPR, simultaneous denitrification and P removal can save aeration, minimize sludge disposal and reduce the demand for the often-limiting carbon sources (Kuba et al., 1993, 1996). The activity of DPAOs has often been demonstrated, both in lab-scale and full-scale EBPR systems (Kuba et al., 1993; Ahn et al., 2002; Zilles et al., 2002; Shoji et al., 2003; Kong et al., 2004).

*Candidatus accumulibacter* has been identified as the most dominant DPAO in the EBPR process in wastewaters (Zeng et al., 2017). The use of 16S rRNA and polyphosphate kinase (*ppk1*) genes as the genetic markers suggested that *Ca. accumulibacter* is divided into two main clades: *Ca. accumulibacter phosphatis* clade I (PAO I) and *Ca. accumulibacter phosphatis* clade II (PAO II) (Rubio-Rincon et al., 2017). Further studies (Carvalho et al., 2007; Flowers et al., 2009; Oehmen et al., 2010) revealed that the PAO I clade was capable of considerable anoxic phosphate uptake activity using nitrate and/or nitrite as an electron acceptor whrereas PAO II lacked the respiratory nitrate reductase enzyme (*nar*), but possessed the mechanisms to denitrify nitrite. During the treatment of municipal wastewater by the EBPR system under complete nitrification, clade I which used nitrate as the electron acceptor, was found to be below 5% of the total *Ca. accumulibacter* population, whereas Clade IID and Clade IIC which used nitrite as the electron acceptor, were always found to be dominant (above 90 and 87.3% of the total population of *Ca. accumulibacter*, respectively), throughout the operational period (Zeng et al., 2016, 2017).

*Tetrasphaera* is another one of the most abundant PAOs present in the EBPR systems, but their capacity to achieve denitrifying P removal has not been determined. The capacity of *Tetrasphaera* to couple denitrification with P uptake has never been established (Marques et al., 2018). Four isolates of *Tetrasphaera* had been shown to possess the genes for the reduction of nitrate to nitrite and some isolates (*T. japonica* and *T. elongata*) had been shown to use nitrate and nitrite as electron acceptors (Lanham et al., 2018). Nevertheless, the amount of anoxic P uptake by a denitrifying *Tetrasphaera*-PAO has been found to be very low (Marques et al., 2018). Till date, the genera *Tetrasphaera* and *Candidatus accumulibacter* appear to be the only known DPAOs to be consistently found in high abundances in full-scale EBPR plants where they could be considered critical to P removal (Nielsen et al., 2019).

In another study, a denitrifying consortium derived from the fluidized bed reactor during the treatment of fish aquaculture effluent, was incubated under laboratory conditions in the presence or absence of nitrate (Barak and van Rijn, 2000a). One of the isolates of denitrifying bacteria (*Paracoccus denitrificans*) isolated from the fluidized bed reactor was characterized in detail for its denitrifying P-removal ability (Barak and van Rijn, 2000a). It was found that in contrast to PAOs, poly-P synthesis by *P. denitrificans* took place only in the presence of an external carbon source under either aerobic or anoxic conditions. Furthermore, unlike PAOs, *P. denitrificans* was unable to utilize PHA as an energy source for poly-P synthesis. Under anaerobic condition, these DPAOs were unable to use external carbon source for PHA synthesis, and degraded glycogen for PHA synthesis; degraded poly-P to release phosphate, which provided energy for growth. Under aerobic/anoxic condition, they produced poly-P and grew utilizing the energy provided by external carbon source and produced glycogen; in the absence of external carbon source, cells with PHA did not grow and did not accumulate poly-P (Kaltwasser et al., 1961; Ferguson and Gadian, 1979; Barak and van Rijn, 2000a). The feasibility of this type of phosphate removal was demonstrated for freshwater as well as marine recirculating systems (Barak and van Rijn, 2000a; Shnel et al., 2002; Barak et al., 2003; Gelfand et al., 2003; Rijn et al., 2006). According to Barak and van Rijn (2000a), the most striking feature of the mode of phosphate uptake and accumulation exhibited by *P. denitrificans* and other denitrifiers examined in their laboratory, was their ability to synthesize poly-P under either aerobic or anoxic conditions without the need for alternating anaerobic/aerobic (anoxic) switches. It has been repeatedly mentioned by these researchers that unlike conventional PAOs, phosphate uptake by these denitrifiers does not require switches between anaerobic/aerobic or anaerobic/anoxic phases (Barak et al., 2003; Rijn et al., 2006; Shi and Lee, 2006; Zheng et al., 2014). According to Medhi and Thakur (2018), this display of single stage simultaneous N and P removal makes the wastewater treatment by these DPAOs a sustainable process, if suitable technologies can be adopted.

Lee and Park (2008) reported isolation of a denitrifying polyphosphate-accumulating bacterium, *Paracoccus* sp. YKP-9, from activated sludge of a 5-stage biological nutrient removal process with a step feed system. This strain also accumulated poly-P with the energy provided by an external carbon source under anoxic condition using nitrate, but neither accumulated poly-P nor grew in the absence of an external carbon source under anoxic condition. Moreover, it did not consume intracellular PHA for poly-P accumulation. Similarly, Shi and Lee (2007) reported a denitrifying phosphorus-removing bacterium, *Brachymonas* sp. strain P12, growing in similar conventional EBPR models, but these models were developed under anoxic or aerobic conditions only, without an anaerobic stage.

In other words, all of the above described DPAOs can combine phosphorus removal and denitrification into a single process using the same amount of organic substrate (Yuan and Oleszkiewicz, 2011). Denitrification is believed to be a strictly anaerobic process (Kaspar, 1982; Tiedje et al., 1982; Payne, 1983) because O_2_ has been shown to suppress the activity of bacterial dissimilatory nitrate reductase (John, 1977; Tiedje et al., 1982; Payne, 1983) although several studies have established that denitrification could also occur in the presence of O_2_ (Voets et al., 1975; Meiberg et al., 1980; Bazylinski and Blakemorer, 1983; Robertson and Kuenen, 1983, 1984a,b; Lloyd et al., 1987; Davies et al., 1989). But O_2_ has no detrimental effect on the denitrifying polyphosphate accumulation activity (Kuba et al., 1996). Yuan and Oleszkiewicz (2011) showed that dissolved O_2_ conc. of 0.01 ± 0.01 mg L^–1^ provided favorable condition for DPAOs to grow under anoxic condition. Altogether with its lower COD requirement (Kuba et al., 1996), and reduced aeration costs, denitrifying phosphorus removal (DNPR) can become a cost-effective choice for removing phosphorus and nitrogen simultaneously.

Several researchers (Barak and van Rijn, 2000a, b; Barak et al., 2003; Rijn et al., 2006; Shi and Lee, 2006) have repeatedly emphasized that in the presence of nitrate, the denitrifying consortium is capable of phosphate uptake in excess of their metabolic requirements. It had been shown by them that the denitrifying consortium present in the fluidized bed reactor, assimilated ammonia and phosphate at a molar N:P ratio ranging from 0.5 to 2.4, with an average molar N:P ratio of 1.9. Taking into account that in general the molar N:P ratio of bacterial biomass varies from 5 to 16 (Brock and Madigan, 1991), it was concluded that only under denitrifying conditions i.e., in the presence of nitrate, phosphate is assimilated in excess of the metabolic requirements of the bacteria comprising the consortium. Phosphorus immobilization took place in the anoxic treatment stages of the system where it accumulated up to 19% of the sludge dry weight (Rijn et al., 2006). This fact attracted our attention. This provided us an indication that in the presence of nitrate, DPAOs will prove to be extraordinarily efficient to defy the inhibitory effects of the Pho regulon.

In this connection, we wanted to look at the Pho regulon gene expression profile during phosphorus uptake in some DPAO isolates, which had been isolated from the sludge of parboiled rice mill effluent. Under this background scenario, this study explored the possible trends of overcoming the negative regulation of PhoU resulting in excessive PO_4_^3–^-P uptake by using DNPR with NO_3_^–^-N at a varying range of concentrations. We initiated research to investigate the Pho regulon gene expression pattern and kinetic studies of P-removal by those DPAOs which were able to remove both PO_4_^3–^-P and NO_3_^–^-N in a single anoxic stage with the utilization of external carbon sources, and without any anaerobic-aerobic or anaerobic-anoxic switches.

## Materials and Methods

### Isolation, Enrichment and Identification of Bacterial Isolates in Search of DPAOs

Sludge sample from a PO_4_^3–^ -rich parboiled rice mill effluent settling tank (containing ∼40 ppm of PO_4_^3–^-P) was serially diluted (up to 10^–9^) in sterile water, and 100 μl of each dilution was spread onto a nutrient agar medium and was incubated at 37°C for 2 days. Repeated subculturing of single colonies was performed to obtain a pure culture of the isolates. The morphological and biochemical characteristics (including nitrate reduction ability) of the isolates were determined. Scanning electron microscopy (SEM) of the isolates was also performed (Zeiss SEM, Central Research Facility, Indian Institute of Technology, Kharagpur, India). Molecular identification of the pure bacterial isolates was carried out by a standard procedure of partial 16S rRNA gene amplification by universal primers. The sequences were subjected to blast search for identification and submitted to the NCBI database. The other biochemical tests to characterize the isolates were also carried out following the standard procedures.

### Growth, Carbon Source Utilization and PO_4_^3–^ Removal Ability of Probable DPAO Isolates and Consortium Under Anaerobic Condition

(1) Twenty four hour old cultures of the individual isolates and an artificial bacterial consortium (raised from the 24 h old culture of all the isolates by suspending a 100 mg cell pellet of each isolate in 10 ml of 0.84% saline solution) were inoculated into their respective screw capped flask containing 250 ml basal level modified synthetic wastewater SW medium (Supplementary Table S1) (Holler and Trösch, 2001). The final OD at 600 nm after inoculation should be 0.1.

(2) Three sets were prepared for each isolate and the consortium, where in the first set 300 ppm meat extract was used as carbon source and in the second set 1000 ppm acetate (added as sodium acetate) was used as carbon source (where no peptone and meat extract was given) and in the third set no carbon source was added. Fifty ppm of PO_4_^3–^-P (added as di-potassium hydrogen phosphate, K_2_HPO_4_) was added to all the flasks. Nitrate-nitrogen was not added in this anaerobic incubation. All the flasks containing media were flushed with pure nitrogen and all the flasks were put in the HiMedia Anaerobic gas jar with Anaero Gas Pack. All the flasks were incubated with 150 rpm shaking at 30°C for 12 h. After the incubation, culture growth in terms of OD at 600 nm, residual PO_4_^3–^-P concentrations and residual acetate concentrations (from acetate containing media) and residual dissolved organic carbon (DOC) concentrations (from meat extract containing media) were measured according to the following protocols.

Acetate was measured according to the protocol of Shi and Lee (2006) with some modifications using the GC-FID (Agilent) with Quadrex 007 Series bonded phase fused silica capillary column (15 m, 0.32 mm ID, 0.25 μm film thickness). Samples were acidified with 1 (N) H_2_SO_4_ to pH below 3. The flow rate of the carrier gas (N_2_) was 3 mL min^–1^. Injector/detector temperature was set to 220/230°C respectively. The column temperature was set in the range of 70°C – 150°C with temperature programing at the rate of 7°C min^–1^. A five point calibration curve was made between concentration and peak area of 100–2000 ppm acetate (added as sodium acetate). DOC was determined by simple spectrophotometric method (Moore, 1985). Absorbance at 330 nm of the media was measured for DOC calculation. The PO_4_^3–^-P conc. of the medium was measured by the molybdenum blue method (Krishnaswamy et al., 2009).

### Growth, Carbon Source Utilization, PO_4_^3–^-P and NO_3_^–^-N Removal Ability of Probable DPAO Isolates and Consortium Under Aerobic and Anoxic Conditions

(1) The inoculation steps were followed exactly as mentioned earlier (in the above section).

(2) Two main sets were prepared – one was SW with 1000 ppm of NO_3_^–^-N (added as potassium nitrate, KNO_3_) and other one was SW without any NO_3_^–^-N. Without NO_3_^–^-N sets were incubated aerobically in cotton plugged flasks and with NO_3_^–^-N sets were incubated anoxically in screw capped flasks. Both the sets had three sub-sets – without any carbon source, with 1000 ppm acetate (added as sodium acetate) and with 300 ppm meat extract. 50 ppm of PO_4_^3–^-P (added as di-potassium hydrogen phosphate, K_2_HPO_4_) was added to all the flasks. In total six different sets were experimentally set up for each isolate and the consortium.

For creating low DO anoxic environment, medium was dispensed in the reaction container with low surface area to volume ratio which limited the diffusion of O_2_ in the medium. To maintain efficient DO free environment, 1 cm of liquid paraffin oil was poured on the surface of the medium (according to Sigma-Aldrich nitrate broth product information). Added excess salt in the medium also created low DO level. The DO level was maintained at 0.01 mg L^–1^ and checked throughout all the experiments by DO meter (Mettler-Tolledo).

All the flasks were incubated with 150 rpm shaking at 30°C for 12 h. After the incubation, culture growth in terms of OD at 600 nm, residual PO_4_^3–^-P concentrations, residual NO_3_^–^-N and NO_2_^–^-N concentrations (from NO_3_^–^-N containing media), residual acetate concentrations (from acetate containing media) and residual dissolved organic carbon (DOC) concentrations (from meat extract containing media) and poly-P accumulation inside the cell were measured.

The NO_3_^–^-N conc. of the medium was determined by measuring the absorbance of nitrosalicylic acid by the spectrophotometric method (Cataldo et al., 1975). NO_2_^–^-N conc. was measured by adding 1% sulfanilic acid-0.05% *N*-naphthylene diamine-HCl dissolved in 1 (M) H_3_PO_4_, and measuring the absorbance spectrophotometrically (Baumann et al., 1996). The accumulated poly-P was extracted and quantified according to an established protocol that was standardized in our laboratory (Mukherjee and Ray, 2015), and is very specific for the polyphosphates having chain length not shorter than 10 residues (Lorenz et al., 1999; Kulaev et al., 2004; Omelon and Grynpas, 2008).

### Study for Maximum PO_4_^3–^-P and NO_3_^–^-N Removal Ability of the DPAO Isolates and the Consortium From Synthetic Wastewater

Modified SW with complex organic carbon source was used to study maximum PO_4_^3–^-P and NO_3_^–^-N removal efficiency of the individual isolates and the developed consortium. Forty-one combinations of SW were prepared with varying conc. of PO_4_^3–^-P (0, 10, 50, 100, 250, 500, and 1000 ppm added as K_2_HPO_4_) and NO_3_^–^-N (0, 50, 100, 500, 1000, and 2000 ppm added as KNO_3_), except for the combination without any PO_4_^3–^-P or NO_3_^–^-N. The individual isolates and the bacterial consortium (initial OD of 0.04 at 600 nm) was inoculated in 500 ml of SW for each combination and was incubated at 30°C for 12 h. The combinations without NO_3_^–^-N were incubated aerobically while combinations with NO_3_^–^-N were incubated at low DO anoxic environment. Evaluation of the amount of accumulated poly-P and estimation of the remaining PO_4_^3–^-P, NO_3_^–^-N and NO_2_^–^-N concentrations were determined at the end of 12 h. In a similar way, the control non-PAO bacteria like *E*. *coli* K 12 ER2925, *E*. *coli* K 12 PR1031 (obtained from New England Biolab, NEB) and *E*. *coli* DH5α strains were inoculated in all the earlier stated N and P combinations in SW, their P and N removal data were ascertained.

All the above experiments were run for 96 h, and the P and N removal data were collected at the end of 12, 24, 72, and 96 h. The growth of the isolates was also recorded by measuring the absorbance at 600 nm at the intervals of 12, 24, 48, 72, and 96 h. In addition, pH of all the media combinations were recorded at the end of 12, 24, 48, 72, and 96 hrs, for all the isolates including the control non-PAO *E. coli* strains.

### Study of Simultaneous PO_4_^3–^-P and NO_3_^–^-N Removal From the Mixture of PO_4_^3–^ Rich Stillage and NO_3_^–^ Rich Explosive Industry Effluent by the DPAO Consortium

Stillage, a PO_4_^3–^-P -rich effluent, derived from a rice-based ethanol producing distillery industry was collected from IFB Agro Industries, Noorpur, West Bengal, India. The NO_3_^–^-N-rich effluent was collected from an explosive manufacturing industry located in Jharkhand, India. All the effluent samples were transported to the laboratory in ice-cold conditions and were stored at 4°C until further analyses. A maximum of 400 ppm of soluble PO_4_^3–^-P was found in the stillage and a maximum of 15000 ppm NO_3_^–^-N was observed to be present in the explosive industry effluent. Different combinations of the effluent mixture were prepared with varying conc. of stillage (containing 0, 175, 200, 350, and 400 ppm PO_4_^3–^-P) and explosive industry effluent (containing 0, 750, 1000, 1500, and 3000 ppm NO_3_^–^-N), except the combination of 0 + 0. As the consortium was found to grow to a maximum of 3000 ppm of NO_3_^–^-N containing explosive effluent, the study was limited within this conc. of NO_3_^–^-N. Since the raw explosive industry effluent did not contain any carbon source, 0.05% peptone and 0.03% meat extract were added to the effluent in the combinations prepared without stillage. The batch reactors of dimensions 0.3 m^∗^0.3 m^∗^0.1 m were constructed, and each was filled with 5 L of different combinations of the effluent mixture and was inoculated with 2.5 g of 24 h old artificial bacterial consortium. The temperature was maintained at 30°C. Low DO environment for anoxic condition was created as described previously. The residual PO_4_^3–^-P, NO_3_^–^-N and NO_2_^–^-N contents were measured at 2, 6, and 12 h intervals.

### Study of the Changes in the Chemical Kinetics of PO_4_^3–^-P Removal in Presence of NO_3_^–^-N

The rates of PO_4_^3–^ and NO_3_^–^-N removal and the order of reaction of the remediation of the SW and effluent mixture by the bacterial consortium were established by a kinetic study by applying the integrated rate laws for the three observed conditions: (i) media combinations containing only PO_4_^3–^-P in aerobic condition, (ii) media combinations containing only NO_3_^–^-N in anoxic condition, and (iii) media combinations containing both PO_4_^3–^-P and NO_3_^–^-N under anoxic condition.

### Quantitative Real-Time PCR Study to Understand the Effect of NO_3_^–^-N on the Regulation of PO_4_^3–^-P Uptake by the Pho Regulon

The four PO_4_^3–^ transporter genes, namely, *PstS*, *PstC*, *PstA*, and *PstB*, and a regulator gene, *PhoU*, which are the units of the bacterial Pho regulon, were selected for this study. The beta subunit of the DNA directed RNA polymerase gene, *RpoB*; 16S rRNA gene; alpha subunit of DNA-directed RNA polymerase, *RpoA*; DNA gyrase A gene, *GyrA*; Recombinase A gene, *RecA* were selected to check their stability index (*M* value) by using the NormFinder software tools (Rocha et al., 2015). For *M* value detection, the expression of all the referred reference genes were evaluated under identical N and P added combinations of SW that were later utilized to find the expression pattern of our candidate genes. The maximum stable gene was used as the reference gene. Two pure isolates, *Escherichia coli* isolate SW11 (Accession no. KU740237–KU740238) and *Bacillus* sp. isolate SW7 (Accession no. KU740235–KU740236), which were found to be the best removers of PO_4_^3–^-P and NO_3_^–^-N (concluded from the experiments performed with individual isolates) and for which the Pho regulon gene sequences were available in public databases, were chosen for this study along with the control non-PAO bacteria, *E*. *coli* K 12 ER2925. The primers were designed with the help of the SCITOOLS site of Integrated DNA Technologies^1^ (Supplementary Table S2). For each of the two isolates, 15 ml of sixteen different combinations of the SW medium were prepared with varying PO_4_^3–^-P conc. (0, 100, 250, and 500 ppm) and NO_3_^–^-N conc. (0, 100, 1000, and 2000 ppm). Five replica sets of each were prepared for five different incubation periods of 1, 2, 4, 6, and 12 h. All of the tubes were inoculated with the overnight grown culture (initial OD of 0.1 at 600 nm) and were incubated at anoxic condition at 30°C for 12 h. Total RNA was extracted from each culture with Purezol RNA isolation reagent (Bio-Rad Laboratories, Hercules, CA, United States) and was quantified by the NanoDrop UV spectrophotometer (Eppendorf Company, Eppendorf, Hamburg, Germany). One μg RNA from each culture was taken for immediate cDNA synthesis by using a cDNA synthesis kit (BioBharati LifeScience Private Limited, Kolkata, West Bengal, India). One μl of each cDNA was used for quantitative real-time PCR (QRT PCR) using iTaq Universal SYBR Green Supermix (Bio-Rad Laboratories, Hercules, CA, United States). The PCR conditions were set as follows: Pre-incubation (1 cycle) – 94°C for 7 min; 3 step amplification (45 cycles) – 94°C for 15 s, 54°C for 40 s, and 72°C for 30 s; melting (1 cycle) – 95°C for 10 s, 65°C for 60 s, and 97°C for 1 s. The final data were analyzed, and accurate normalization was performed with the reference gene expression level.

### Statistical Analysis

Studies in triplicate sets were carried out for all of the experiments. All of the represented data were the mean ± standard deviation. The standard deviation was depicted with error bars in each graphical presentation. SigmaPlot 13.0 and Microsoft Excel software were used to prepare the graphical presentations. To observe the significance of changes in different parameters over a number of days, Duncan’s multiple range test was performed for each data with SPSS 13.0 software. Analysis of variance (ANOVA) along with Duncan’s multiple range test was used to determine if there were significant differences in the measured parameters. Values designated with different letters are significantly different at the 5% level.

## Results and Discussion

### Isolation and Identification of the Probable DPAOs

Scanning electron microscopy images were recorded for all 13 pure bacterial isolates (Supplementary Figure S1). 16S rRNA gene identification of the pure isolates segregated them into three main genera, *Bacillus*, *Escherichia*, and *Staphylococcus*, and the NCBI accession numbers of their sequences are shown in Supplementary Table S3. The SEM images depict long and short rod morphology for the *Bacilli*, bunch of cocci morphology for the *Staphylococci* and normal morphology for *E. coli*. The Gram characters and biochemical characteristics of all the isolates are presented in the Supplementary Figure S2. The most important biochemical character which was found to be common for all the isolates was that all these isolates were positive for nitrate reduction test. This significant finding led us to set up the subsequent experiments to confirm whether this denitrifying ability of the isolates was accompanied by poly-phosphate accumulating ability also. Possession of both the referred properties by the isolates would have confirmed their denitrifying poly-phosphate accumulating (DPAO) nature. The control non-PAO bacteria like *E*. *coli* K 12 ER2925, *E*. *coli* K 12 PR1031 and *E*. *coli* DH5α strains used in this study are established denitrifier strains (Stewart et al., 2002; Clegg et al., 2006).

### Growth, Carbon Source Utilization and PO_4_^3–^ Removal Ability of Probable DPAO Isolates and Consortium Under Anaerobic Condition

**FIGURE 1:**
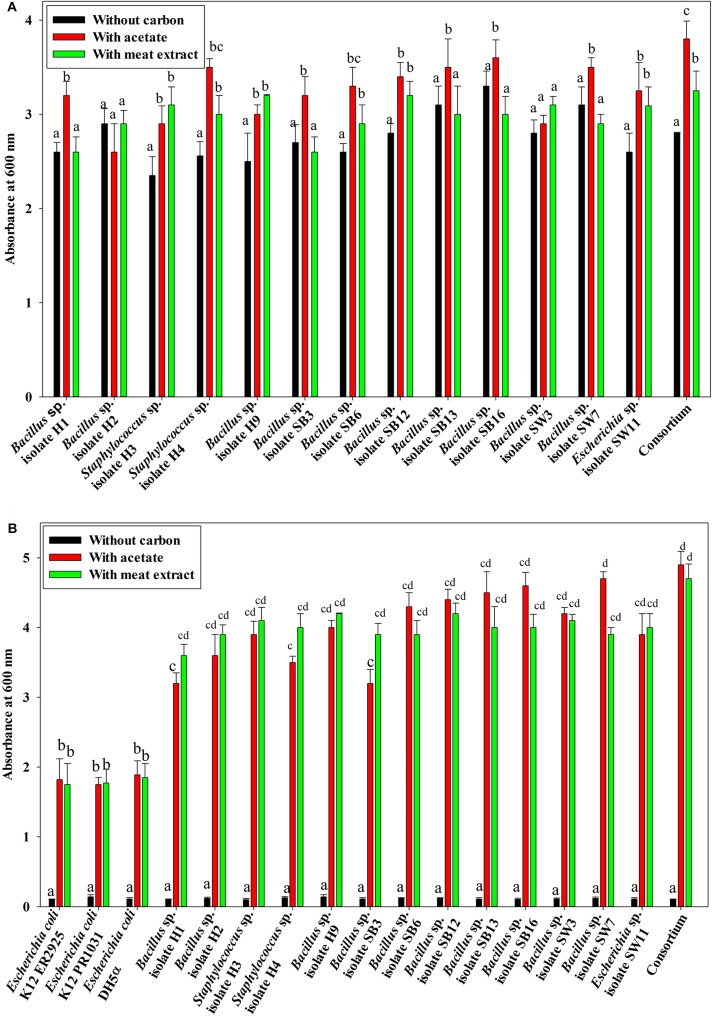
**(A)** Growth of the 13 DPAO bacterial isolates and their consortium under anaerobic condition with differential carbon sources in 12 h of culture. **(B)** Average anoxic and aerobic growth of the control non-PAO, *E*. *coli*, the 13 DPAO bacterial isolates and their consortium over 12 h in culture conditions with different carbon sources. Values designated with different letters are significantly different at the 5% level.

**FIGURE 2:**
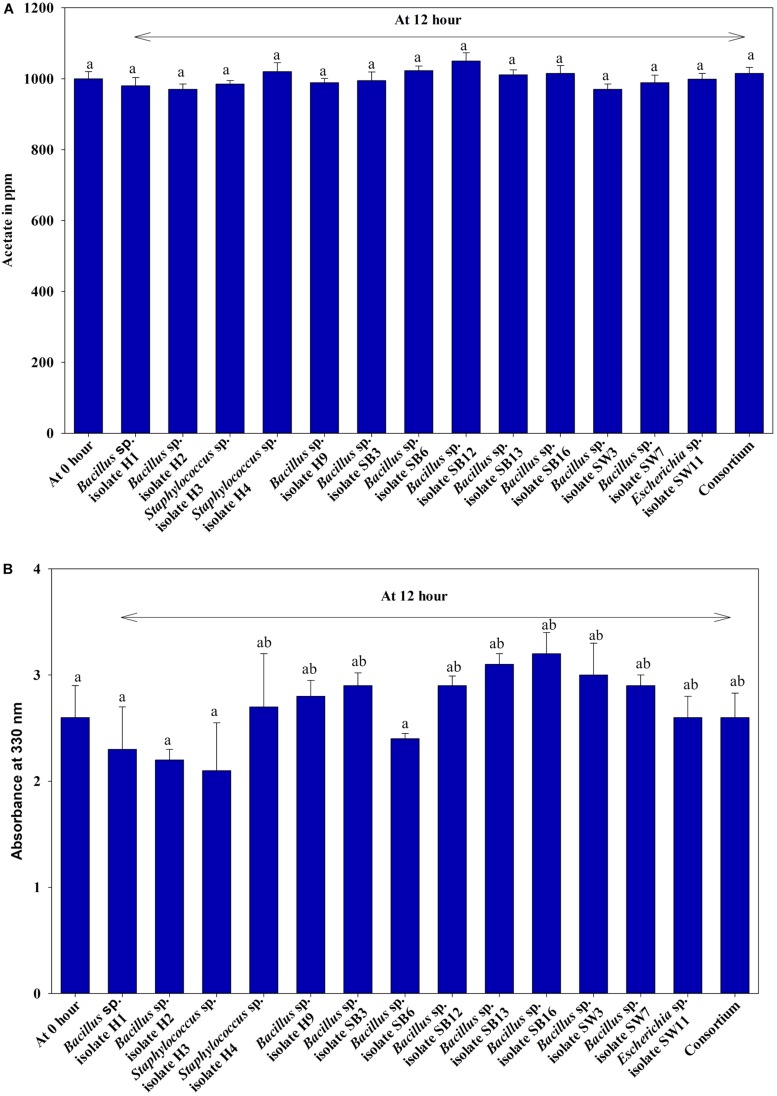
**(A)** Residual acetate concentration in the medium where only acetate was used as carbon source for 12 h of anaerobic growth of the 13 DPAO isolates and their consortium. **(B)** Residual dissolved organic carbon (DOC) in the medium supplemented with meat extract as carbon source for 12 h of anaerobic growth of the 13 DPAO isolates and their consortium. Values designated with different letters are significantly different at the 5% level.

Under anaerobic condition, no significant difference in growth between the individual isolates and the consortium was observed irrespective of the carbon sources used or without any carbon sources (Figure 1A). No significant difference in growth among the isolates and the consortium was also observed when VFA (acetate) or complex organic carbon source (meat extract) were used as the carbon sources (Figure 1A). When acetate was used as the carbon source, initial acetate concentration added was 1000 ppm in the medium. But no fall from this initial concentration was observed in the media even after 12 h as no isolate including consortium utilized this acetate as carbon source (Figure 2A). But growth rate of all the isolates was appreciable in acetate containing medium (Figure 1A). When meat extract was used as the carbon source, again no utilization of carbon source was observed even after 12 h of growth (Figure 2B). Some of the isolates rather released DOC during their growth. No significant difference in DOC utilization was observed between the individual isolates and the consortium (Figure 2B). Thus, it can be concluded that under anaerobic condition, our experimental bacterial isolates and their artificially made consortium failed to utilize the external carbon sources added. This non-utilization of carbon sources in anaerobic phase is very unusual for conventional DPAOs and has only been reported for *P. denitrificans* DPAO isolated by Barak and van Rijn (2000a).

**FIGURE 3:**
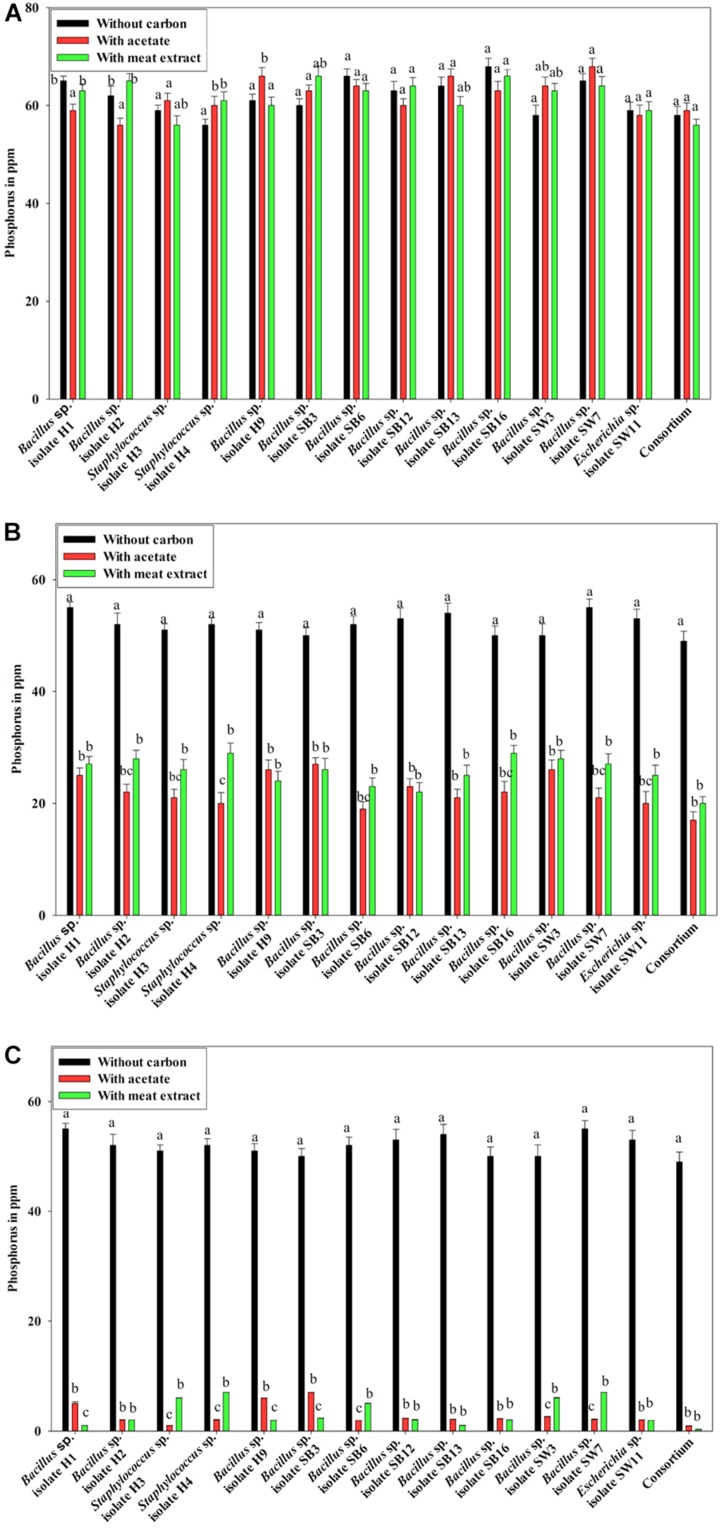
**(A)** Residual phosphate concentration (starting concentration 50 ppm) in the media after 12 h of anaerobic growth supplemented with different carbon sources for the 13 DPAO isolates and their consortium. **(B)** Residual phosphate concentration in the media (starting concentration 50 ppm) after 12 h of aerobic growth supplemented with different carbon sources for the 13 DPAO isolates and their consortium. **(C)** Residual phosphorus (starting concentration 50 ppm) in the medium with different carbon sources/no carbon sources after 12 h of anoxic growth of the 13 DPAO isolates and their consortium in the presence of 1000 ppm NO_3_^–^-N. Values designated with different letters are significantly different at the 5% level.

Additionally, the PO_4_^3–^-P removal scenario was also intriguing. Under anaerobic phase, no PO_4_^3–^-P removal was observed (Figure 3A). No reduction from the initial PO_4_^3–^-P concentration (50 ppm) was observed even after 12 h of culture (Figure 3A), instead PO_4_^3–^-P release occurred in all the cases irrespective of the carbon sources present (Figure 3A). Maximum 15 ppm extra PO_4_^3–^-P was released. No significant difference in PO_4_^3–^-P release was displayed by the individual isolates or the consortium. It was reported that denitrifying polyphosphate-accumulating bacterium *Paracoccus* sp. strain YKP-9 also released approximately 15.1 mg L^–1^ of PO_4_^3–^-P during the anaerobic phase in 48 h (Lee and Park, 2008).

These observations of anaerobic phase strikingly resembled denitrifying PAO *P. denitrificans* (Barak and van Rijn, 2000a) which was also unable to use external carbon source for PHA synthesis under anaerobic condition and released phosphate by degradation of poly-phosphates that provided energy for growth. The anaerobic phase growth criteria, carbon utilization pattern and PO_4_^3–^-P release characteristics pointed toward confirmation of the non-conventional DPAO nature of our denitrifying bacterial isolates and their respective consortium.

### Growth, Carbon Source Utilization, PO_4_^3–^-P and NO_3_^–^-N Removal Ability of Probable DPAO Isolates and Consortium Under Aerobic and Anoxic Conditions

**FIGURE 4:**
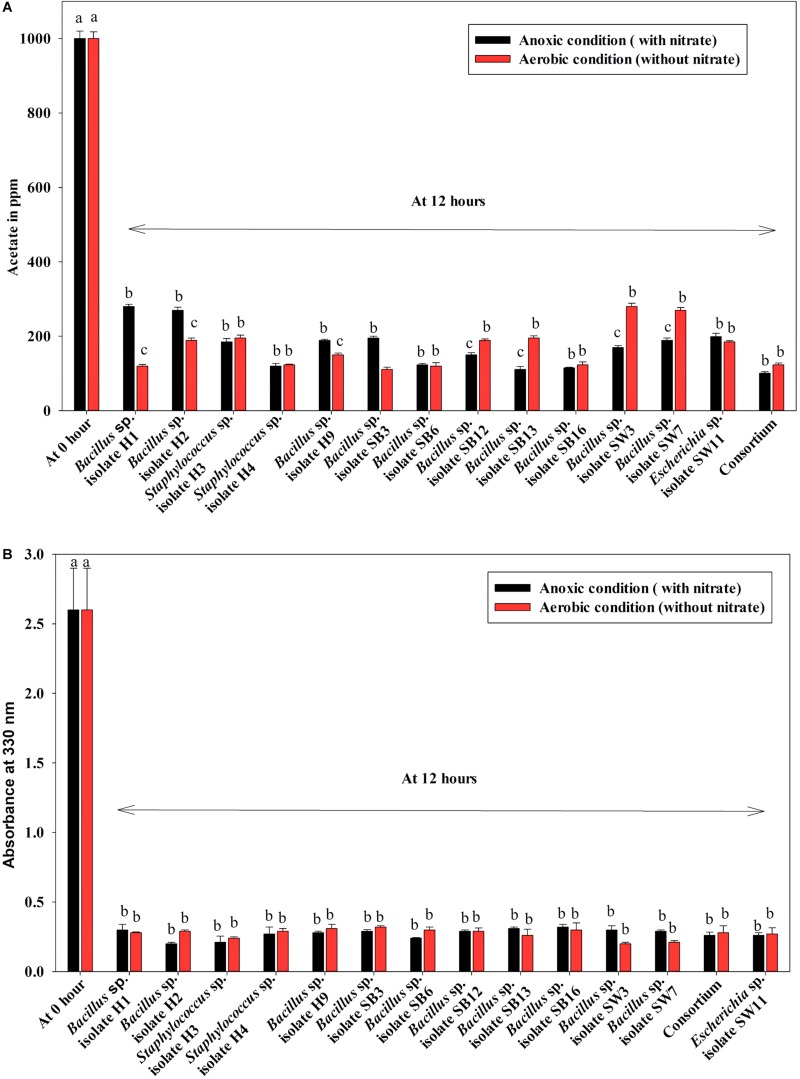
**(A)** Residual acetate in the medium supplemented with sodium acetate as the carbon source under both anoxic and aerobic growth after 12 h for the 13 DPAO isolates and their consortium. **(B)** Residual dissolved organic carbon (DOC) in the medium supplemented with meat extract as the carbon source under both anoxic and aerobic growth after 12 h for the 13 DPAO isolates and their consortium. Values designated with different letters are significantly different at the 5% level.

Under both aerobic and anoxic phases, no growth could be observed in the media with no carbon sources, whereas appreciable growth was observed for media with carbon sources (Figure 1B). Under both aerobic and anoxic phases, media with acetate and media with meat extract as the sole carbon sources, did not exhibit any significant difference in growth parameter (Figure 1B). The consortium showed considerable growth rate in both phases irrespective of the carbon sources used (Figure 1B). All the isolates and the consortium were found to utilize acetate efficiently from the medium (Figure 4A). The H1, H2 isolates efficiently used acetate in aerobic growth phase whereas the SW3, SW7 isolates used it efficiently in anoxic growth phase (Figure 4A). Rest of the isolates including the consortium efficiently used acetate in both the stages. All the isolates and the consortium were also found to efficiently utilize the DOC from the media containing meat extract as the complex organic carbon (Figure 4B). Therefore, it can be inferred that these probable DPAO isolates and their artificially made consortium needed external carbon sources in aerobic as well as in anoxic phase for their growth and to carry out their relevant metabolic activities. This finding is exactly opposite to the carbon utilization mode of the conventional DPAO like *Ac. candidatus*. The only other DPAOs reported to utilize carbon sources under both aerobic and anoxic conditions are *P. denitrificans* (Barak and van Rijn, 2000a) and *Paracoccus* sp. (YKP-9) (Lee and Park, 2008).

Furthermore, when the PO_4_^3–^-P removal activity was considered, interesting results were observed. In the media without any carbon source, where no bacterial growth was observed, PO_4_^3–^-P removal activity was also negative under both the aerobic and anoxic phases. But all the isolates including the consortium removed 50–55% of PO_4_^3–^-P in 12 h of aerobic growth in the absence of NO_3_^–^-N irrespective of the added carbon sources (Figure 3B) while all the isolates including the consortium removed more than 90% of PO_4_^3–^-P in 12 h of anoxic growth in the presence of 1000 ppm of NO_3_^–^-N irrespective of the added carbon sources (Figure 3C). This important finding led us to summarize that these denitrifying isolates and their consortium were capable of growth and PO_4_^3–^-P removal, both aerobically and anoxically, but they removed PO_4_^3–^-P at an accelerated rate under anoxic phase in the presence of NO_3_^–^-N in the media. Thereafter, all our subsequent experiments on PO_4_^3–^-P removal by these DPAOs were conducted under single anoxic phase in the presence of varying concentrations of NO_3_^–^-N. We assumed that this DNPR which resulted in excessive PO_4_^3–^-P removal/uptake would have been impossible without being accompanied by the withdrawal of *PhoU* gene mediated negative regulation.

**FIGURE 5:**
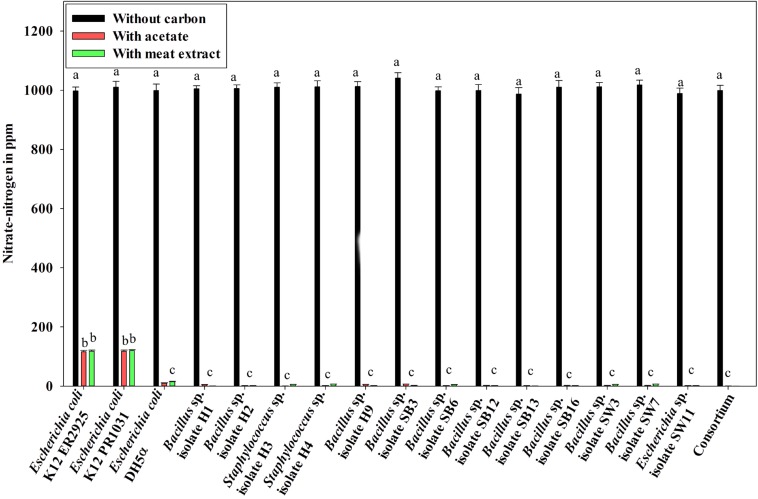
Residual NO_3_^–^-N (initial concentration 1000 ppm) in the media with different carbon sources/no carbon sources after 12 h of anoxic growth of the control non-PAO, *E. coli*, the 13 DPAO isolates and their consortium. Values designated with different letters are significantly different at the 5% level.

Denitrification activity under anoxic growth phase was thus observed as follows: (a) no denitrification was observed in media without any carbon sources (Figure 5); (b) almost 100% NO_3_^–^-N from the medium was removed irrespective of the carbon sources present in the medium (either acetate or meat extract) in 12 h of anoxic growth (Figure 5). We could not trace the presence of any NO_2_^–^-N in the media at the end of 12 h of anoxic growth. It proved that during denitrification NO_2_^–^-N was further reduced to its maximum reduced forms.

**FIGURE 6:**
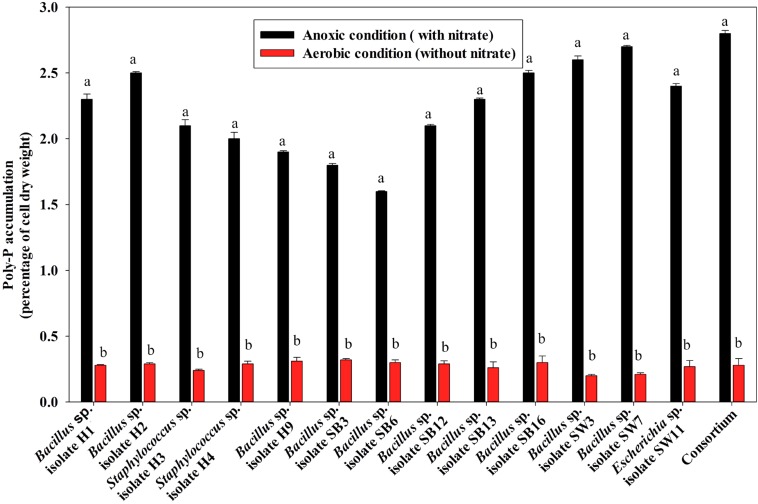
Poly-P accumulation by the 13 DPAO isolates and their consortium after 12 h of anoxic growth (in the presence of 1000 ppm of NO_3_^–^-N) and aerobic growth (in absence of NO_3_^–^-N) from 50 ppm initial PO_4_^3–^-P concentration in the medium. Values designated with different letters are significantly different at the 5% level.

For poly-P accumulating activity, about 9–11 fold higher poly-P accumulation than that of the aerobic growth, was observed when the isolates and the consortium were grown anoxically for 12 h in the presence of 1000 ppm of NO_3_^–^- N (Figure 6). The consortium showed the highest (2.7% of cell dry weight, CDW) accumulation in 12 h of anoxic growth phase (Figure 6). These results signified that the bacterial isolates and the consortium developed in this study were actually DPAOs, like *P. denitrificans* (Barak and van Rijn, 2000a), which were capable of simultaneous PO_4_^3–^-P and NO_3_^–^-N removal under single anoxic growth phase without the need for alternating anaerobic/aerobic or anaerobic/anoxic switches. No significant difference in PO_4_^3–^-P and NO_3_^–^-N removal could be detected in the experimental set ups with either acetate or meat extract as the sole carbon sources. For this reason, further studies were carried out with SW media with meat extract as the complex carbon source (Natcheva and Beschkov, 2003). For some of the subsequent remediation experiments, only the artificial DPAO consortium was used because apart from having high PO_4_^3–^-P and NO_3_^–^-N removal capabilities, it also showed the best poly-P accumulation rate over the individual isolates (Figure 6).

### Concurrent PO_4_^3–^-P and NO_3_^–^-N Removal With Concomitant Poly-P Accumulation by the DPAO Isolates and the Consortium From SW

**FIGURE 7:**
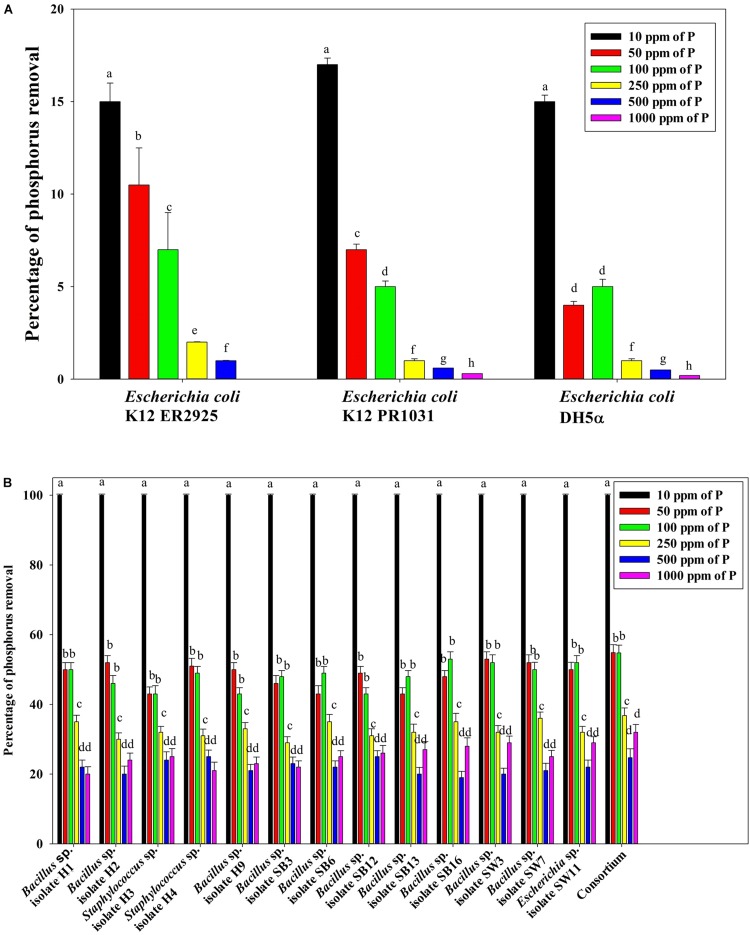
**(A)** PO_4_^3–^-P removal by the non-PAO *E*. *coli*. strains from different initial PO_4_^3–^-P concentrations after 12 h of aerobic growth in the absence of NO_3_^–^-N. **(B)** PO_4_^3–^-P removal by the 13 DPAO isolates and their consortium from different initial PO_4_^3–^-P concentrations after 12 h of aerobic growth in the absence of NO_3_^–^-N. Values designated with different letters are significantly different at the 5% level.

**FIGURE 8:**
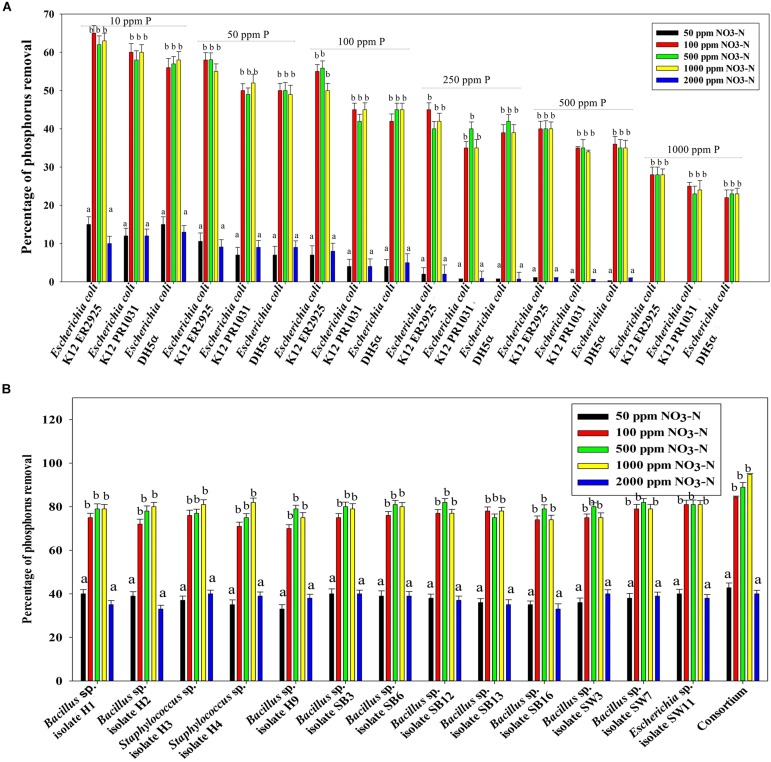
**(A)** PO_4_^3–^-P removal by the non-PAO *E*. *coli*. strains from different initial PO_4_^3–^-P concentrations (10–1000 ppm of PO_4_^3–^-P) after 12 h of anoxic growth in the presence of different initial NO_3_^–^-N concentrations. **(B)** PO_4_^3–^-P removal by the 13 DPAO isolates and their consortium from 250 ppm initial PO_4_^3–^-P concentration after 12 h of anoxic growth in the presence of different initial NO_3_^–^-N concentrations. Values designated with different letters are significantly different at the 5% level.

**FIGURE 9:**
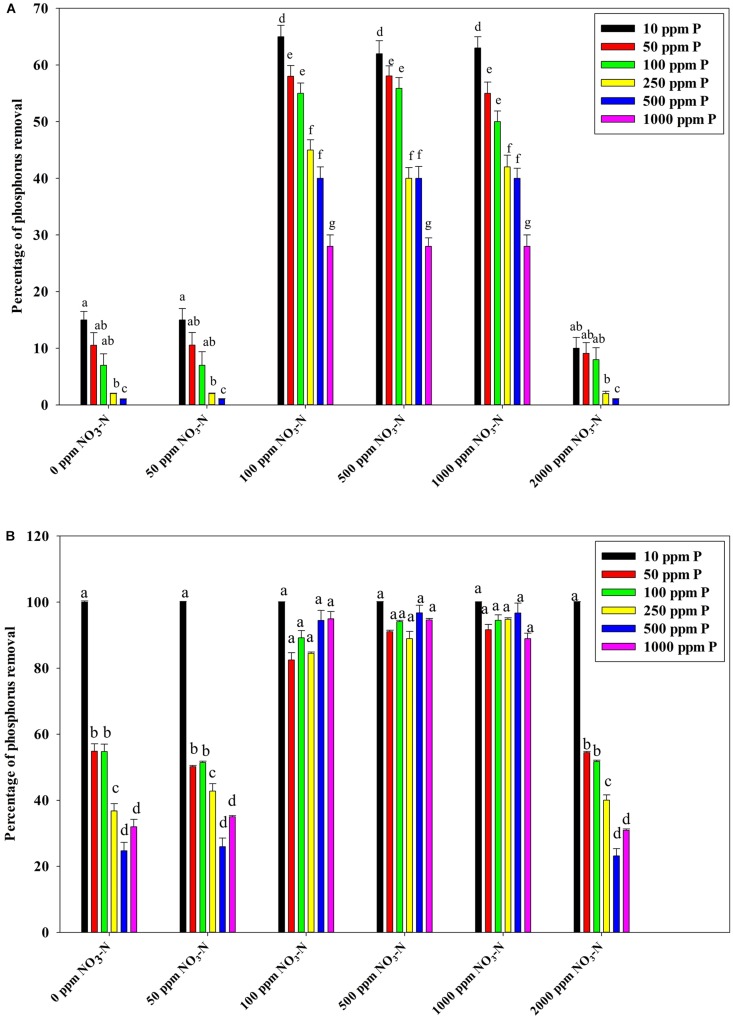
**(A)** PO_4_^3–^-P removal by the non-PAO, *E*. *coli*. K12 ER 2925 from different initial PO_4_^3–^-P concentrations after 12 h of anoxic growth in the presence of different initial NO_3_^–^-N concentrations. **(B)** PO_4_^3–^-P removal by the consortium of the 13 DPAO isolates from different initial PO_4_^3–^-P concentrations after 12 h of anoxic growth in the presence of different initial NO_3_^–^-N concentrations. Values designated with different letters are significantly different at the 5% level.

**FIGURE 10:**
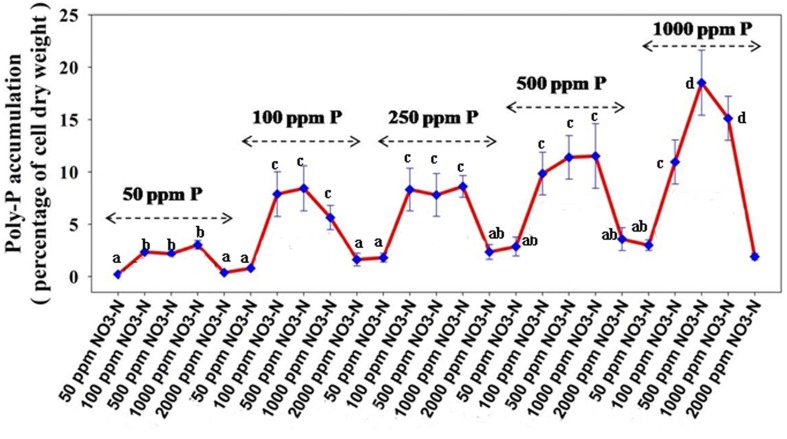
Poly-P accumulation concurrent with PO_4_^3–^-P removal from synthetic wastewater (SW) in combinations of 50–1000 ppm of PO_4_^3–^-P and 50–2000 ppm of NO_3_^–^-N by the consortium of the 13 DPAO isolates. Values designated with different letters are significantly different at the 5% level.

**FIGURE 11:**
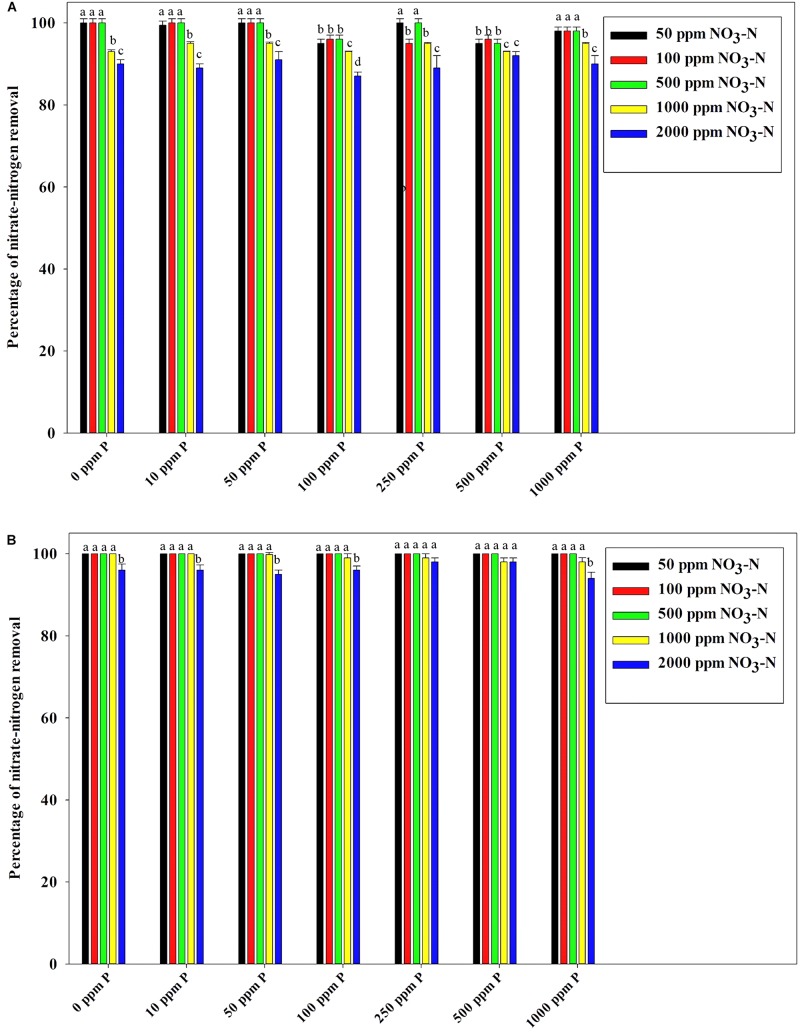
**(A)** NO_3_^–^-N removal by the non-PAO, *E*. *Coli*. K12 ER 2925 from different initial NO_3_^–^-N concentrations after 12 h of anoxic growth in the presence of different initial PO_4_^3–^-P concentrations. **(B)** NO_3_^–^-N removal by the consortium of 13 DPAO isolates from different initial NO_3_^–^-N concentrations after 12 h of anoxic growth in the presence of different initial PO_4_^3–^-P concentrations. Values designated with different letters are significantly different at the 5% level.

Figure 7B reveals an interesting scenario of PO_4_^3–^-P removal by the DPAOs in the absence of NO_3_^–^-N in SW medium, under aerobic condition. The percentage of PO_4_^3–^-P removal (at the end of 12 h) receded gradually with an increasing conc. of initial PO_4_^3–^-P, i.e., PO_4_^3–^-P removal falling from ∼100% when the initial PO_4_^3–^-P was 10 ppm to 25–30% in case of 1000 ppm of initial PO_4_^3–^-P. Hence, we considered any external PO_4_^3–^-P conc. above 10 ppm as P-replete condition in this study. In contrast, the control non-PAO denitrifiers removed only about 15% PO_4_^3–^-P from 10 ppm initial conc. to only 1–2% P-removal from 1000 ppm of initial PO_4_^3–^-P conc. in 12 h (Figure 7A). However, when 100, 500, and 1000 ppm of NO_3_^–^-N were added to the SW under anoxic environment, a distinct 3–4-fold increase in the PO_4_^3–^-P removal percentage was observed at the very end of only 2 h, and 85–100% P removal was observed within 12 h, irrespective of the initial PO_4_^3–^-P conc. (Figures 8B, 9B). By contrast, 0, 50, and 2000 ppm of NO_3_^–^-N added combinations failed to elicit any such increase in the PO_4_^3–^-P removal percentage (Figures 8B, 9B). Similarly, in the three control non-PAO denitrifiers, *E*. *coli* K 12 ER2925, *E*. *coli* K 12 PR1031 and *E*. *coli* DH5α strains, 55–65% PO_4_^3–^-P removal from 10 ppm initial conc. to 30–35% PO_4_^3–^-P removal from 1000 ppm initial conc. was observed for 100, 500, and 1000 ppm of NO_3_^–^-N concentrations (Figures 8A, 9A). This result provided a clue that the addition of 100–1000 ppm NO_3_^–^-N to phosphorus-rich SW under anoxic condition might be critical for achieving the exceptionally high PO_4_^3–^-P removal (∼85–100%) by the DPAO consortium (Figure 9B). A very congruous poly-P accumulation scenario was also achieved, where the poly-P accrual by the DPAO consortium also increased dramatically to 5–10%, 7.5–10%, 7.5–15%, and 10–22% of CDW from SW containing 100, 250, 500, and 1000 ppm of the initial PO_4_^3–^-P, respectively, in all of the 100, 500, and 1000 ppm of NO_3_^–^-N added combinations (Figure 10). Thus, the fact that 100–1000 ppm of NO_3_^–^-N acted as the inducer concentrations, which resulted in a 3–4-fold increase in the PO_4_^3–^-P uptake and correspondingly accelerated the poly-P accumulation, was constantly proven by these results. The consortium and the control non-PAO *E. coli.* denitrifiers also demonstrated a very high denitrification rate of approximately 90–100% within 12 h for 50–2000 ppm of initial NO_3_^–^-N conc. combined with the 0–1000 ppm of initial PO_4_^3–^-P combinations (Figures 11A,B), nullifying any effect of the PO_4_^3–^-P addition on NO_3_^–^-N removal from SW. No presence of any NO_2_^–^-N in the media at the end of 12 h of anoxic growth was observed repeatedly. We presume that within the 12 h period of denitrification, NO_2_^–^-N was reduced to its maximally reduced gaseous forms.

Although all of the above experiments were continued till 96 h, it was found that insignificant increase in P removal occurred after 12 h (Supplementary Figures S3, S4) with 90–100% nitrate being removed within the 12 h period. Simultaneously, analysis of the growth curves of each of the isolates from each of the P and N combinations over 96 h revealed identical growth increase for each of the isolates till 24 h, after which they reached the stationary phase (Supplementary Figure S5). The growth data demonstrated that the exponential phase of the isolates continued till 24 h.

Increase of pH over 96 h was observed in the media combinations with nitrate because of denitrification, with starting pH at 7 and the maximally increased pH at 7.6 (Supplementary Table S4). Any P-removal caused by chemical precipitation of phosphates at this increased pH can be easily ruled out as this observed pH is not at all favorable for chemical precipitation of insoluble phosphates (Kofina and Koutsoukos, 2005; Daneshgar et al., 2018; Simoes et al., 2018).

### Simultaneous PO_4_^3–^-P and NO_3_^–^-N Removal From the Mixture of PO_4_^3–^-P Rich Stillage and NO_3_^–^-N Rich Explosive Industry Effluent by the DPAO Consortium

**FIGURE 12:**
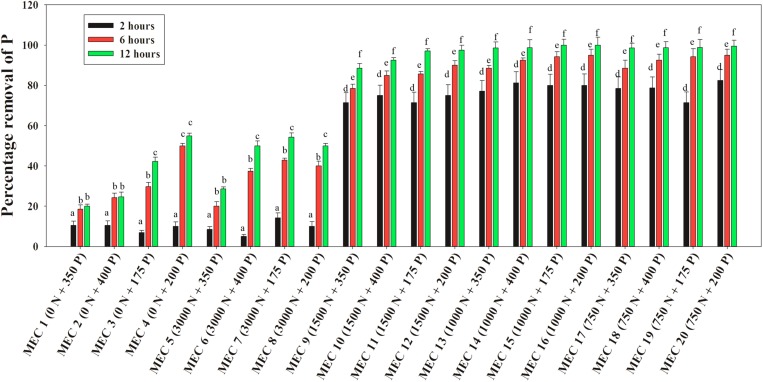
Percentages of PO_4_^3–^-P removal over 12 h by the consortium of the 13 DPAO isolates from the mixed effluent medium with different combinations of PO_4_^3–^-P and NO_3_^–^-N concentration attained by mixing stillage from the rice-based distillery industry and effluent from the explosives industry where MEC stands for Mixed Effluent Combination. Values designated with different letters are significantly different at the 5% level.

A similar situation of the NO_3_^–^-N mediated inductive effect on PO_4_^3–^-P removal was observed when the DPAO consortium was applied to actual wastewaters. Stillage combinations having 175, 200, 350, and 400 ppm of the initial PO_4_^3–^-P conc. mixed with an explosive industry effluent of 750, 1000, and 1500 ppm of NO_3_^–^-N conc., were found to acquire a 6–8-fold increase in PO_4_^3–^-P removal within the initial 2 h, and finally, within 12 h, 85–100% P removal was achieved in comparison to 20–55% P removal in combinations without NO_3_^–^-N (Figure 12). The inductive effect of NO_3_^–^-N on PO_4_^3–^-P removal was again observed along the range of 750–1500 ppm NO_3_^–^-N. Similarly, 100% NO_3_^–^-N removal was also complete within 12 h from all of the mixed effluent combinations (Supplementary Figure S6), once more justifying the absence of any kind of effect of the initial PO_4_^3–^-P conc. on NO_3_^–^-N removal.

### Chemical Kinetics of PO_4_^3–^-P and NO_3_^–^-N Removal and the Observed Inductive Effect of NO_3_^–^-N Addition on PO_4_^3–^-P Removal

**FIGURE 13:**
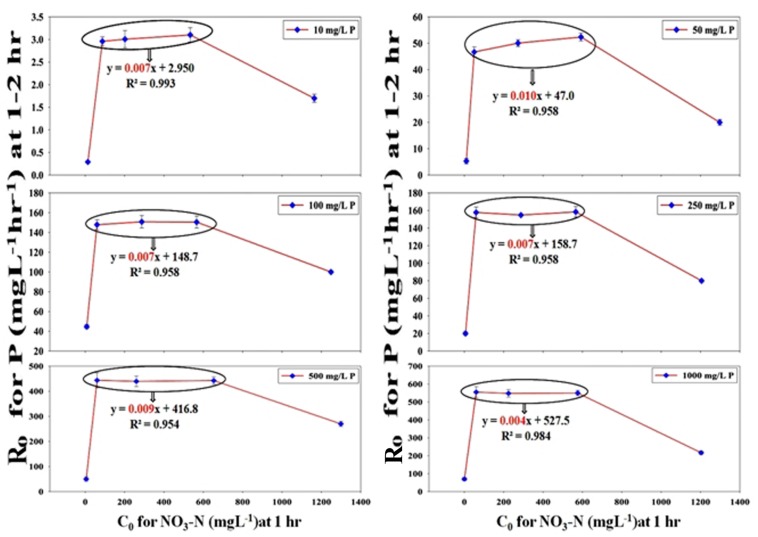
Chemical kinetics of the inductive effect of NO_3_^–^-N on PO_4_^3−^-P removal achieving a stable maximally induced state as observed by plotting the initial rate of PO_4_^3−^-P removal (R_0_) at 1–2 h against the initial conc. of NO_3_^–^-N (C_0_) at the end of 1 h for 100, 500 and 1000 ppm of NO_3_^–^-N (within the bubble) from SW by the consortium of the 13 DPAO isolates. The straight line equation within the bubble nearing an *m* value (slope) of almost 0 justified that maximum induction in R_0_ that already had occurred at C_0_ attained for 100 ppm of NO_3_^–^-N at the end of 1 h and was maintained at the same level for the C_0_ obtained for 1000 ppm of NO_3_^–^-N. An initial PO_4_^3−^-P conc. of 10–1000 ppm and initial NO_3_^–^-N conc. of 50–2000 ppm in different combinations are shown. Uninduced R_0_ was seen at 1–2 h in combinations with 50 and 2000 ppm of C_0_ at the end of 1 h.

Using classical chemical kinetics, the apparent reaction orders of PO_4_^3–^-P and NO_3_^–^-N removals were established and the dependency of PO_4_^3–^-P removal on NO_3_^–^-N addition was also proven strongly by the kinetic models. The PO_4_^3–^-P removal pattern from SW and the effluent mixture containing only stillage and no NO_3_^–^-N followed the zero-order kinetics for the 12 h period, which was observed from the graph plotted as X_*A*_ $=\frac{{\left[P\right]}_{0}-\left[P\right]}{{\left[P\right]}_{0}}$, along the Y-axis and the time (t) along the X-axis, where [P] is the conc. of PO_4_^3–^-P at a certain time and [P]_0_ is the initial conc. of PO_4_^3–^-P at *t* = 0. The plot with an *R*^2^ value that varied from 0.960–0.979 gave straight lines that passed through the origin with slopes (k/[P]_0_) decreasing from 0.080 for 10 ppm of PO_4_^3–^-P to 0.007 for 1000 ppm of PO_4_^3–^-P (Supplementary Figure S7A). In contrast, for the NO_3_^–^-N removal pattern from both SW and mixed effluent in combinations with or without PO_4_^3–^-P, -log_*e*_(1-X_*A*_) was plotted along the *Y*-axis and time (t) was plotted along the *X*-axis. The plots followed first-order kinetics and gave straight lines that passed through the origin with an *R*^2^ value that varied from 0.949–0.993 (Supplementary Figure S7B) to 0.913–0.990 (Supplementary Figure S7C). Interestingly, PO_4_^3–^-P removal in the presence of 50–2000 ppm NO_3_^–^-N followed neither zero- nor first- or second-order kinetics. Hence, the method of the initial rates was used to determine the apparent order. The value of R_0_ (initial rate of the reaction) was calculated from the initial PO_4_^3–^-P conc. at certain times, t_1_ and t_2_ (t_2_ > t_1_) following the equation, $R_{0}=\frac{{\left[P\,O_{4}^{3-}\right]}_{t_{2}}-{\left[P\,O_{4}^{3-}\right]}_{t_{1}}}{t_{2}-t_{1}}$ (Atkins et al., 2018). When the initial PO_4_^3–^-P removal rate, R_0_ (for 10–1000 ppm of PO_4_^3–^-P between times 2 and 1 h) along the *Y*-axis, was plotted against the initial NO_3_^–^-N conc., C_0_ (50–2000 ppm at time 1 h) along the *X*-axis, a 4–8-fold increase in the PO_4_^3–^-P removal rate was noticed (Figure 13). In the graphical representation, a linear plateau achieved for the PO_4_^3–^-P removal in the NO_3_^–^-N addition range of 100–1000 ppm implied that a steady state rate of the PO_4_^3–^-P uptake was reached, and further NO_3_^–^-N addition no longer induced any more appreciable increase. The kinetic analyses again reaffirmed the fact that the addition of a minimum conc. of 100 ppm of initial NO_3_^–^-N within the very first hour of remediation was enough to induce the highest PO_4_^3–^-P removal rate from both the SW and the mixed effluents.

### Validation of the PO_4_^3–^-P Removal Kinetics and the Inductive Effect of NO_3_^–^-N on Pho Regulon Genes by a Quantitative Real-Time PCR Study

**FIGURE 14:**
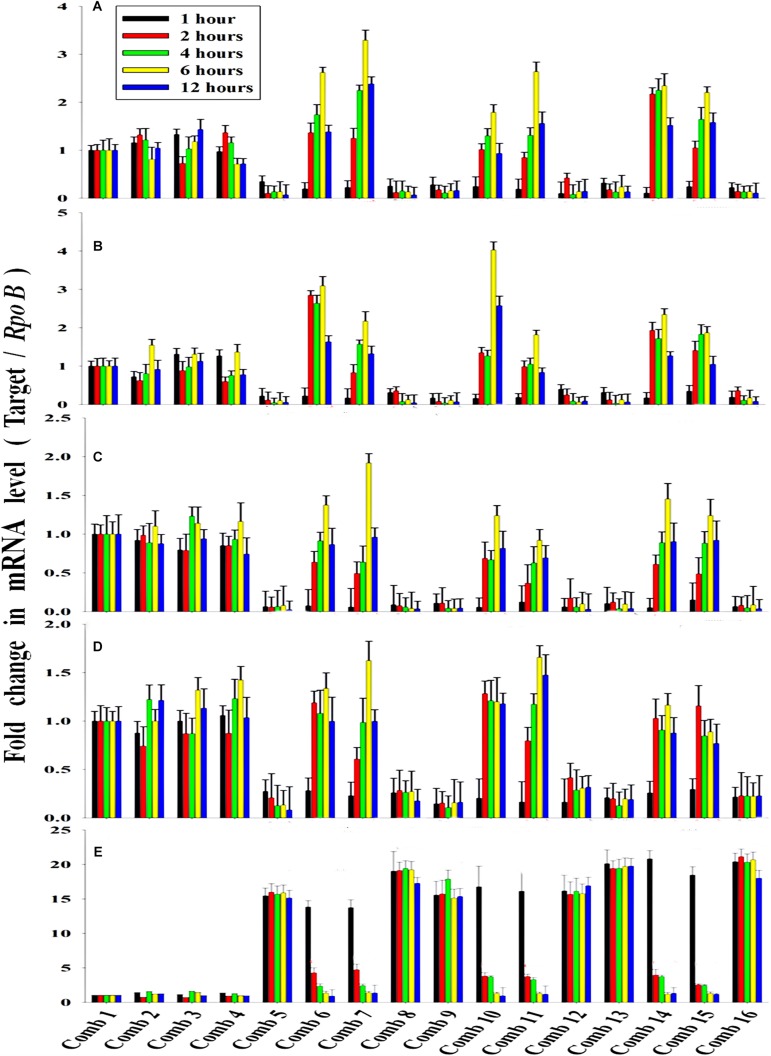
The average transcript level of Pho regulon genes- **(A)** *PstS*, **(B)** *PstC*, **(C)** *PstA*, **(D)** *PstB*, **(E)** *PhoU* of the *Bacillus* sp. isolate SW7 (Accession no. KU740235-KU740236) and the *Escherichia coli* isolate SW11 (Accession no. KU740237-KU740238) over a period of 12 h when grown in SW with the following combinations of PO_4_^3–^-P and NO_3_^–^-N. Comb 1–4: 0 ppm PO_4_^3–^-P with 0, 100, 1000, 2000 ppm of NO_3_^–^-N; Comb 5–8: 100 ppm PO_4_^3–^-P with 0, 100, 1000, 2000 ppm of NO_3_^–^-N; Comb 9–12: 250 ppm PO_4_^3–^-P with 0, 100, 1000, 2000 ppm of NO_3_^–^-N; Comb 13–16: 500 ppm PO_4_^3–^-P with 0, 100, 1000, 2000 ppm of NO_3_^–^-N where Comb stands for Combinations.

**FIGURE 15:**
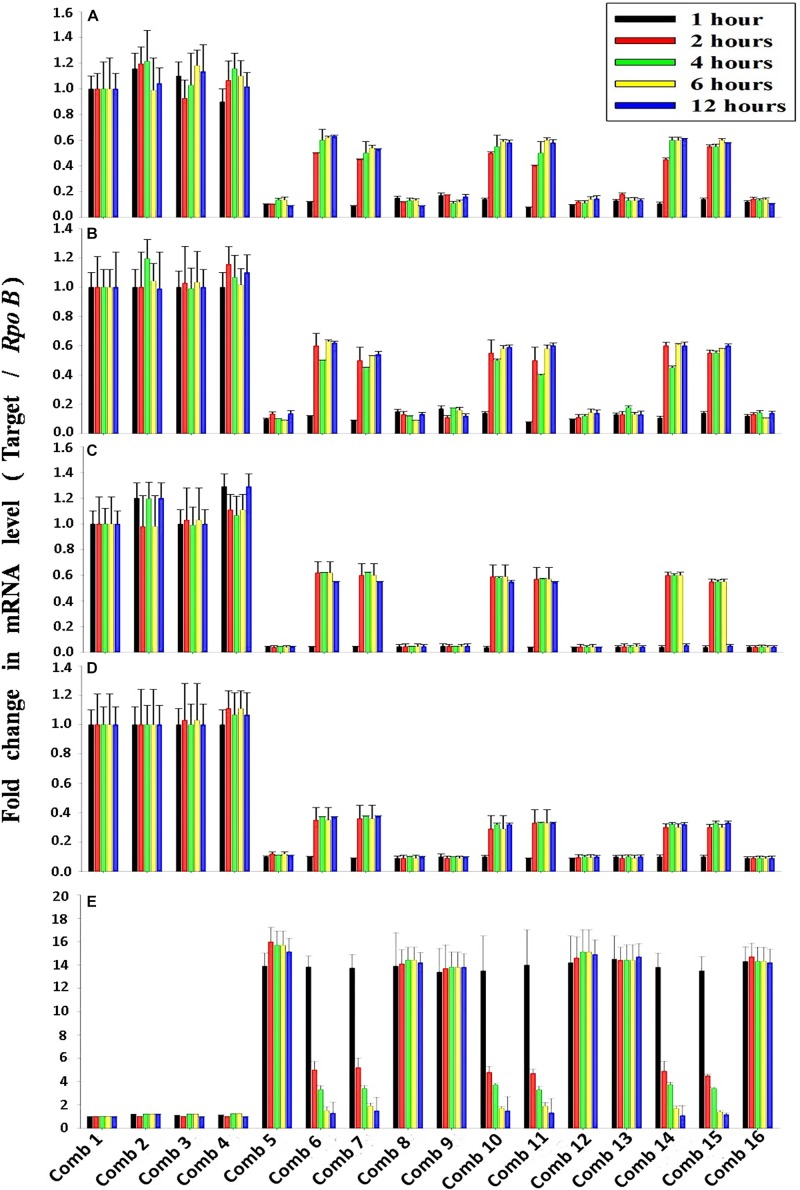
The average transcript level of Pho regulon genes- **(A)** *PstS*, **(B)** *PstC*, **(C)** *PstA*, **(D)** *PstB*, **(E)** *PhoU* of the non-PAO, *E*. *Coli*. K12 ER 2925 over a period of 12 h when grown in SW with the following combinations of PO_4_^3–^-P and NO_3_^–^-N. Comb 1–4: 0 ppm PO_4_^3–^-P with 0, 100, 1000, 2000 ppm of NO_3_^–^-N; Comb 5–8: 100 ppm PO_4_^3–^-P with 0, 100, 1000, 2000 ppm of NO_3_^–^-N; Comb 9–12: 250 ppm PO_4_^3–^-P with 0, 100, 1000, 2000 ppm of NO_3_^–^-N; Comb 13–16: 500 ppm PO_4_^3–^-P with 0, 100, 1000, 2000 ppm of NO_3_^–^-N where Comb stands for Combinations.

The average transcription level of the four transporter genes, *PstS*, *PstC*, *PstA* and *PstB*, and the negative regulator gene, *PhoU*, relative to P removal with added NO_3_^–^-N from the *Escherichia coli* isolate SW11 and the *Bacillus* sp. isolate SW7 is depicted in Figure 14. As a comparison the expression level of the same five candidate genes are also displayed in Figure 15 from the identical media combinations for the control non-PAO *E. Coli.* K 12 ER 2925. *RpoB* gene was selected as the reference gene for proper normalization as it possessed the lowest *M* value (highest stability) (Supplementary Figure S8). The expression of the four *Pst* transporters was always maintained at a high basal level from the initial 1st hour until 12 h in PO_4_^3–^-P deplete conditions to refute any effect of the added NO_3_^–^-N (Figures 14A–D, 15A–D). This supported earlier reports which stated that during P-starved conditions, the *Pst* genes were highly expressed (Hirota et al., 2013). However, the moment that the PO_4_^3–^-P replete situation was created by adding 100, 250, or 500 ppm of PO_4_^3–^-P to the medium, the expression of the *Pst* genes decreased drastically, even at the very 1st hour, and even when NO_3_^–^-N was added to the medium (Figures 14A–D, 15A–D). The repression of the Pho regulon during external PO_4_^3–^-P rich conditions had also been observed by researchers earlier (Muda et al., 1992). Intriguingly, from the 2nd hour until the 12th hour, *PstS*, *PstC*, *PstA*, and *PstB* expression levels started to increase and were reinstated to the original level, if not higher, for the combinations of 100, 250, and 500 ppm of PO_4_^3–^-P with 100–1000 ppm of NO_3_^–^-N (Figures 14A–D) in contrast to the restoration of the expression of the same P-transporter genes for *E. Coli* K 12 2925 to 40–60% of its original basal level (Figures 15A–D). The sustenance of this high expression of *Pst* transporters under the PO_4_^3–^-P replete condition was contrary to earlier reported observations. We speculated that the addition of NO_3_^–^-N might have played an inductive role in an unknown mechanism to withdraw the repression of the Pho regulon by restoring *Pst* transporter expression. It was again seen that even a minimum of 100 ppm NO_3_^–^-N addition under a highly PO_4_^3–^-P rich environment (up to 500 ppm PO_4_^3–^-P in the experiments) could solely induce the Pho regulon by causing a high expression of *Pst* transporter genes. The 100, 250, and 500 ppm of PO_4_^3–^-P with 2000 ppm of NO_3_^–^-N added failed to elicit any such restoration of *PstSCAB* expression level (Figures 14A–D, 15A–D). When no PO_4_^3–^-P was present, the *PhoU* gene was observed to be downregulated for the studied 12 h period, despite the added NO_3_^–^-N (Figures 14E, 15E), with a simultaneous upregulation of the *Pst* genes. However, the moment that the PO_4_^3–^-P replete condition was achieved with the addition of 100, 250, or 500 ppm of PO_4_^3–^-P, the *PhoU* expression was significantly upregulated to 15–20-fold from the very 1st hour until the 12th hour, even in the absence of NO_3_^–^-N in the medium (Figures 14E, 15E), with simultaneous down regulation of the *Pst* genes. Amazingly, upon the addition of 100 and 1000 ppm of NO_3_^–^-N under PO_4_^3–^-P rich conditions (100, 250, and 500 ppm of P), the expression of *PhoU* started to reduce significantly from the 2nd hour of incubation, and this 15–20-fold downfall continued steadily until the 12th hour (Figures 14E, 15E). The down regulated *PhoU* expression level at a high PO_4_^3–^-P conc. with 100–1000 ppm of added NO_3_^–^-N strangely resembled the environment without external PO_4_^3–^-P. The down regulation of *PhoU* was accompanied by a concurrent up regulation of *Pst* transporter genes, which agreed with earlier studies that had established the PhoU protein as a negative regulator of Pho regulon (Gardner et al., 2014). However, the addition of even 100 ppm NO_3_^–^-N withdrew the negative regulation by *PhoU* unexpectedly, even under a PO_4_^3–^-P replete state, by some unknown mechanism. As long as *PhoU* was down regulated by adding NO_3_^–^-N, the concurrent high expression of *PstSCAB* transporters could be achieved, which accelerated the PO_4_^3–^-P uptake (almost 100% uptake even from 1000 ppm of PO_4_^3–^-P replete environment) from both PO_4_^3–^-P rich SW and mixed effluents. However, it was again observed that 2000 ppm of NO_3_^–^-N addition failed to down regulate the *PhoU* expression in all the experimental strains (Figures 14E, 15E).

In a recent study (Wang et al., 2018) with *Dolichospermum flos-aquae*, a diazotrophic cyanobacterium, the expression levels of genes involved in PO_4_^3–^-P uptake, such as the gene for the hydrolysis of phosphomonoesters, *PhoD*, and the PO_4_^3–^ transporter, *PstS*, were observed to be upregulated in PO_4_^3–^-P deficient cultures upon nitrogen (N) addition. N addition also enhanced poly-P formation and alkaline phosphatase activity in PO_4_^3–^-P deficient cultures. During the PO_4_^3–^-P deficient treatment, the expression levels of *PstS* and *PhoD* were significantly down regulated during the initial 2–4 days, but a 2.8-fold up regulation was observed 6–8 days later in *PstS*, and a 1.2–8.6-fold up regulation was seen in *PhoD*, in the absence of N. However, after N addition, the *PstS* expression was up regulated by 10.7-fold on day 12, and the *PhoD* expression was up regulated by 17.9-fold after day 8, even in the PO_4_^3–^-P deficient treatment. Interestingly, in the above study (Wang et al., 2018), the up regulation of *PstS* and *PhoD* genes occurred upon the addition of N in PO_4_^3–^-P deplete conditions, whereas in the case of the DPAO isolates used in this study, NO_3_^–^-N addition enhanced the expression of *PstSCAB* genes even under PO_4_^3–^-P replete conditions, although the gene was normally reported to be down regulated in bacteria under a PO_4_^3–^-P replete environment (Muda et al., 1992; Hirota et al., 2013).

### NO_3_^–^-N as the Transcriptional Repressor of the *PhoU* Gene

The most surprising result presented in this study was the demonstration that NO_3_^–^-N at a specific concentration limit, i.e., within 100–1000 ppm, acted as an activator of the Pho regulon even under PO_4_^3–^-P replete condition. Usually Pho regulon is known to be activated by external PO_4_^3–^-P starvation signal and by PhoB-PhoR two component system. But in this case, during the DNPR process, in response to a completely opposite signal of excess PO_4_^3–^-P in external environment (in response to which Pho regulon is normally destined to get shut off), Pho regulon gets activated in presence of 100–1000 ppm of NO_3_^–^-N. Under PO_4_^3–^-P replete external environment, PhoB is dephosphorylated by PhoR-PhoU complex (Wanner, 1992), but this intervention by PhoU seems to be far from possible here as the *PhoU* expression itself is found to be down regulated since the second hour of NO_3_^–^-N addition till the 12 h period (Figures 14E, 15E). Hence, we assume that the sensor PhoB is phosphorylated by normal kinase activity of PhoR at this period without facing any repression from PhoU. We speculate that 100-1000 ppm of NO_3_^–^-N by some unknown mechanism is repressing the expression of *PhoU* for this period, but we also have to remember that during this 12 h period NO_3_^–^-N is also being simultaneously removed almost at about 100% removal rate from the wastewater/culture media by the DPAO isolates and their consortium. Hence, 100–1000 ppm of NO_3_^–^-N can be considered to be only the initial inducing concentration needed to repress the expression of *PhoU* gene and the repression is maintained uninterruptedly even after this NO_3_^–^-N concentration is gradually decreased to near zero over the 12 h period. At this juncture, we could only assume that NO_3_^–^-N must be having an unusual role in the initiation of transcriptional inhibition of *PhoU*, but how it is being implemented does not fall within the experimental designs of our study. A similar repression was reported in *E. coli* where *PFL* gene encoding the enzyme pyruvate formate-lyase, a key enzyme of anaerobic metabolism catalyzing the non-oxidative cleavage of pyruvate to acetyl coenzyme A and formate, was regulated by nitrate (Kaiser and Sawers, 1995). It was demonstrated that the expression of the Pfl operon was negatively regulated by nitrate and when bacterial cells were cultured in minimal medium this regulation was markedly stronger than when they were cultured in rich medium. Nitrate regulation of Pfl operon was proved to be mediated exclusively by the dual sensors, NarX and NarQ and the dual transcription regulators NarL and NarP (Kaiser and Sawers, 1995). Nitrate signaling in bacteria is known to be mediated by the homologous sensor proteins NarX and NarQ (Stewart, 1993). Mutational analyses have defined a heptamer sequence necessary for specific DNA binding by the NarL protein. These heptamers are located at different positions in the regulatory regions of different operons. The response regulator, NarL binds DNA to control nitrate induction and repression of the genes encoding nitrate respiration enzymes and alternate anaerobic respiratory enzymes, respectively (Stewart, 1993). In another instance, repression of gene expression was reported in *hyaABCDEF* and *hybOABCDEFG* operons of *E. coli* encoding for hydrogenase 1 and hydrogenase 2 enzymes respectively, in presence of nitrate as the terminal electron acceptor (Richard et al., 1999). Nitrate repression could however be relieved in a *narL narP* double mutant, indicating that both NarL and NarP mediated nitrate repression of these two operons. In this study also, nitrate was used as the terminal electron acceptor as an anoxic low DO environment was maintained throughout the experiments. We are still unable to confer at this stage that the nitrate repression of *PhoU* gene is mediated by NarL/NarP protein binding to the upstream sequences of *PhoU*, although we could hypothesize that this nitrate mediated repression might involve NarL/NarP protein binding to the upstream elements of *PhoU* based on earlier observations on similar nitrate mediated transcriptional repression. In *Burkholderia pseudomallei*, biofilm formation was found to be inhibited in response to exogenous nitrate (Mangalea et al., 2017). Transposon insertional mutants of *NarL*, the DNA-binding response regulator, *NarX*, the nitrate sensor, *NarG-1* and *NarH-1*, the alpha and beta subunits of major nitrate reductase and of *NarK-1*, a nitrate/nitrite transporter, demonstrated insensitivity to biofilm inhibition by nitrate. These results suggested that nitrate sensory cascades and nitrate metabolism pathway were somehow linked to and responsible for biofilm inhibition based on nitrate availability. Apart from these, nitrate was also found to inhibit other alternative respiratory pathways in bacteria. In *Dechloromonas aromatica* strain RCB, in transition studies from aerobic metabolism through nitrate reduction to perchlorate reduction, increase in the level of transcripts necessary for nitrate and perchlorate reduction was observed concomitantly with decrease in the concentration of exogenous nitrate and perchlorate respectively, suggesting that nitrate negatively regulates transcription of perchlorate reductase thus inhibiting perchlorate reduction leading to preferential utilization of nitrate (Sun and Coates, 2007). All these studies indicated that nitrate mediated transcriptional repression is quite widespread among the microbial community. In eukaryotic system also, supply of exogenous nitrate was found to immediately induce the expression of a transcriptional repressor gene in rice, designated as *NIGT1* (Nitrate-Inducible, GARP-type Transcriptional Repressor 1) (Sawaki et al., 2013).

### A Single Phase Denitrifying Phosphorus Removal Process

This whole study was based on a single anoxic phase denitrifying phosphorus removal process by the DPAO isolates and their consortium. Our established DPAOs are found to be quite different from the conventional PAOs. PAO needs two stages – anaerobic and aerobic for efficient PO_4_^3–^-P removal. In anaerobic phase, they use external carbon source like VFA to produce PHA (polyhydroxyalkanoates), and the stored glycogen and polyphosphate is broken down and phosphorus is released to the external environment. In aerobic phase, they degrade the PHA and produce polyphosphate intracellularly through the uptake of PO_4_^3–^-P from the external environment. Therefore, for PAOs, there is no need of external carbon source in this aerobic phase when actual PO_4_^3–^-P removal is bound to happen in EBPR system. But our DPAOs produce poly-phosphate only in the presence of external carbon source under anoxic condition where nitrate acts as an electron acceptor. In absence of external carbon sources, cells with PHA do not grow and do not produce poly-phosphate. Not only that, in their anaerobic growth phase, they are also unable to utilize the external carbon sources like PAOs to produce PHA. Rather they produce PHA by degrading internal poly-phosphate or glycogen in anaerobic phase (Barak and van Rijn, 2000a). Our developed DPAOs showed similar characteristics under anaerobic phase growth and only their anoxic growth phase opened up the scope for simultaneous biological PO_4_^3–^-P and NO_3_^–^-N removal carried out in this study with NO_3_^–^ as the terminal electron acceptor, where external carbon sources present in the effluent/SW could be utilized to its optimum, surpassing the need of anaerobic stage. The EBPR mechanism of DPAO *Brachymonas* sp. strain P12 was similar to the conventional anaerobic–aerobic (or anaerobic–anoxic) EBPR models, but these models were developed under anoxic or aerobic conditions only, without an anaerobic stage (Shi and Lee, 2007). In their search for DPAO isolates from piggery sludge, enriched sludge, and winery sludge, Shi and Lee (2006) combined anoxic denitrifying ability with aerobic–anoxic PO_4_^3–^-P removal and compared it with conventional anaerobic–aerobic and anaerobic–anoxic PO_4_^3–^-P removal. They finally optimized a single-stage anoxic PO_4_^3–^-P removal process with NO_3_^–^ as the final electron acceptor for the culture, isolated DPAOs and achieved simultaneous PO_4_^3–^-P and NO_3_^–^-N removal efficiencies of 96 and 86%, respectively from a single stage anoxic reactor. Our results obtained in this study completely matched their findings. This DNPR process, performed in a single stage anoxic phase, also provided evidences of excessive PO_4_^3–^-P removal, defying the negative regulation of PhoU protein in the presence of a certain conc. range of NO_3_^–^-N.

While executing the remediation of mixed effluent, the carbon sources utilized were the complex carbon sources of PO_4_^3–^-P rich stillage which were easily fermented to produce alcohol along with the unfermented complex carbon sources of VFAs, whereas in experiments with SW, added meat extract were the complex carbon sources utilized. Study of Natcheva and Beschkov (2003) has already established that anoxic denitrification occurs well in the presence of complex carbon sources like meat extract and in our study we did not get any significant differences in terms of PO_4_^3–^-P and NO_3_^–^-N removal where VFA like acetate was used as the sole carbon source instead of meat extract. Our study thus described a single anoxic phase denitrifying PO_4_^3–^-P removal with NO_3_^–^ as terminal electron acceptor in the presence of external complex carbon sources like meat extract. The denitrification phenomenon was monitored mainly in terms of decreasing concentration of NO_3_^–^ from the effluent/SW. We have tested whether this denitrification phenomenon resulted in reduction of nitrate to nitrite. But nitrite could not be traced at the end of 12 h experiments even after repetitive measurements, which made us to conclude that nitrite had been maximally reduced to its gaseous forms through this denitrification process.

### Significance of the Findings and Practical Implications

Simultaneous removal of PO_4_^3–^-P and NO_3_^–^-N from wastewaters has long been described by several workers (Lee et al., 2001; Hu et al., 2003; Wang et al., 2009; Yang et al., 2010; Yamashita and Yamamoto-Ikemoto, 2014; Delgadillo-Mirquez et al., 2016; Wan et al., 2017; Zhang et al., 2017), but all of those studies relied on a two-reactor system with a continuous switching between the aerobic-anaerobic-anoxic-aerobic cycles. A recent approach that reported PO_4_^3–^-P and NO_3_^–^-N removal efficiencies of 97.8 and 98.2%, respectively, required an obligatory aerobic/anaerobic system for the anammox reaction and a requisite aerobic/anaerobic/anoxic process for denitrifying P removal (Zhang et al., 2017). In another approach, simultaneous PO_4_^3–^-P and NO_3_^–^-N removal efficiencies of 94 and 91%, respectively, were reported from domestic wastewater in a two-sludge sequencing batch reactor following an anaerobic-anoxic/nitrification process with the necessity of maintaining the influent (chemical oxygen demand, COD) COD/P and COD/Total N ratios of 19.9 and 9.9, respectively (Wang et al., 2009). A novel sequencing batch moving bed membrane bioreactor for wastewater treatment was also reported, where the total PO_4_^3–^-P and NO_3_^–^-N removal efficiencies averaged at 82.6 and 84.1%, respectively, with the PO_4_^3–^-P remediation competence being dependent on the length of the aerobic/anaerobic cycle and varying considerably if the switch did not occur at the appropriate time (Yang et al., 2010). In India, the upflow anaerobic sludge blanket (UASB) reactor was designed especially for PO_4_^3–^-P removal from industrial effluents based on the anaerobic treatment of the wastewater with methanogenic bacteria, but the maintenance of the anaerobic condition with a removal efficiency of just 10–50 ppm of PO_4_^3–^-P rendered this system to be comparatively incompetent (Haridas, 2010). These operational complexities and the high maintenance cost of the reactor systems often lead to the closure of wastewater treatment plants in industries, especially in India and in other developing countries. In contrast, in the present study, a simplified single anoxic phase batch reactor system with a compatible denitrifying polyphosphate accumulating bacterial consortium was reported, which is a perfect combination to unequivocally remediate the excess PO_4_^3–^-P and NO_3_^–^-N from wastewater simultaneously or individually. This study explicitly proved that the addition of only 100 ppm of NO_3_^–^-N to PO_4_^3–^-P rich wastewater would result in 100% PO_4_^3–^-P removal, thereby defying the limitation of the PO_4_^3–^-P uptake by the repression of Pho regulon in a PO_4_^3–^-P replete environment. This finding, if exploited in the EBPR system, could overcome the limitation in PO_4_^3–^-P removal. The corresponding elevated accumulation of poly-P by the bacterial consortium has a high potential to be used as a PO_4_^3–^ biofertilizer. The experiments performed here were restricted to a 5 L system; however, further successful scaling-up to higher volumes that are suitable for industrial purposes is necessary for a worthwhile application of this study.

## Data Availability Statement

All datasets generated for this study are included in the article/Supplementary Material.

## Author Contributions

CM designed the study, performed the experiments, analyzed the results, and contributed toward preparation of the manuscript. RC and MB performed the experiments and helped in manuscript writing. SG helped in designing the primers for real-time PCR. RB and BC performed the kinetic analyses. KR as a supervisor, identified the research problem, conceptualized and designed the work, and wrote the manuscript.

## Conflict of Interest

The authors declare that the research was conducted in the absence of any commercial or financial relationships that could be construed as a potential conflict of interest.

## Footnotes

1 https://eu.idtdna.com/scitools/applications/realtimepcr/
