# Supplementary material for: Effect of Varying Nitrate Concentrations on Denitrifying Phosphorus Uptake by DPAOs With a Molecular Insight Into Pho Regulon Gene Expression
Source: Front Microbiol. 2019 Nov 8;10:2586. doi: 10.3389/fmicb.2019.02586 (PMC6856094; doi:10.3389/fmicb.2019.02586)
Supplement: Supplementary file 1 [file Table_1.docx]

**Effect of varying nitrate concentrations on denitrifying phosphorus uptake by DPAOs with a molecular insight into Pho regulon gene expression**

Chandan Mukherjee^1^, Rajojit Chowdhury^1^, Mst. Momtaj Begam^1^, Sayak Ganguli^2^, Ritabrata Basak^3^, Basab Chaudhuri^4^, Krishna Ray^1*^

^1^Environmental Biotechnology Group, Department of Botany, West Bengal State University, Berunanpukuria, Malikapur, Barasat, Kolkata 700126, India

^2^Theoretical and Computational Biology Division, AIIST and The Biome, West Bengal, India

^3^Department of Biochemistry, Ballygunge Science College, University of Calcutta, 35, Ballygunge Circular Road, Kolkata 700019, India

^4^Vice Chancellor, West Bengal State University, Berunanpukuria, Malikapur, Barasat, Kolkata 700126, India

^*^Corresponding author email: kray91@gmail.com

**Supplementary Table S1.** **Composition of the modified synthetic wastewater (SW).**

| **Ingredients** | **Amount (in ppm)** |
| --- | --- |
| Peptic digest of animal tissue | 500.0 |
| Meat extract | 300.0 |
| Sodium chloride | 3000.0 |
| Calcium chloride dihydrate | 8.0 |
| Magnesium sulphate heptahydrate | 4.0 |
| pH | 7.0 ± 0.2 |

**Supplementary Table S2.** **Primer sequences (5'-3') for quantitative real-time PCR.**

| Gene | Forward (F)/ Reverse (R) | Sequence | Amplicon Length (in base pair) | |
| --- | --- | --- | --- | --- |
| *Escherichia coli* isolate SW11 (Accession no. KU740237-KU740238) | | | | |
| *PstS* | F | AGACTGGAGCAAAACCTTCG | 126 | |
|  | R | ACTTCTGTGCCTTGTTCTGG |  |  |
| *PstC* | F | TGTGCTATTGATGTTGGGTGG | 121 | |
|  | R | GTAGATATCGTTCGGTGCATCC |  |  |
| *PstA* | F | CGGGCTGTTAATTTTGTGGG | 85 | |
|  | R | TTTACGACCATATTCCGCCAG |  |  |
| *PstB* | F | TGAAGCAGGATTACACCGTG | 134 | |
|  | R | TGGCTTGGTGAACAGATCG |  |  |
| *PhoU* | F | TGGTAAGTTTGGAGTCGCTG | 141 | |
|  | R | CAATACCTTCGTACTCCTGATCG |  |  |
| *RpoB* | F | TGTAACTGCGGTAGCTAAACG | 139 | |
|  | R | AGAACGGGTGTATTTGGTCAG |  |  |
| *16S rRNA* | F | AAGTCGAACGGTAACAGGAAG | 140 | |
|  | R | CTTTGTGCTTGCGACGTTATG |  |  |
| *RpoA* | F | GTGACCCTTGAGCCTTTAGAG | 116 | |
|  | R | ACACCATCAATCTCAACCTCG |  |  |
| *GyrA* | F | CACCAGGGCTACGTTAAGTATC | 137 | |
|  | R | ATATGGTCGTGAGTGTTCGC |  |  |
| *RecA* | F | GAAGATCGGTCAGGGTAAAGC | 134 | |
|  | R | CATCTACAGAGAAATCCGGCG |  |  |
| *Bacillus* sp.isolate SW7 (Accession no. KU740235-KU740236) | | | | |
| *PstS* | F | ACATGCCATTGCTTTTGTAGG | | 141 |
|  | R | GTAAGTAGCGGCCTAGATAACG | |  |
| *PstC* | F | CGTTAGCGGTTCAAATGGTAATC | | 125 |
|  | R | CCAAGCAGTTCCGTTCAATG | |  |
| *PstA* | F | AGTCTTGGATTAGGTGCAACG | | 140 |
|  | R | CCCGCTGTGTAAATTAATGCTG | |  |
| *PstB* | F | TGGGTCTAATGCCGATGTTG | | 149 |
|  | R | CAGCGATTTGGGATGAGTTAAAAG | |  |
| *PhoU* | F | ACAGCGACAGATTTAGAGCG | | 96 |
|  | R | GTGCAAACTAACAGCCACTTC | |  |
| *RpoB* | F | CAGTTAGTCCGCGTATACATCG | | 131 |
|  | R | ACCCCTTTGTTACCATGTCG | |  |
| *16S rRNA* | F | AAGTGTTAGAGGGTTTCCGC | | 137 |
|  | R | CGAATTAAACCACATGCTCCAC | |  |
| *RpoA* | F | ACGTGTGACTTACCAAGTGG | | 137 |
|  | R | GCTCAGTTAAGATTTTGGCACC | |  |
| *GyrA* | F | CATGGTGATTCAGCCGTTTATG | | 144 |
|  | R | CATTCTTGCCTCTGTATAACGC | |  |
| *RecA* | F | GTGGTCGTGCGTTGAAATTC | | 150 |
|  | R | CAACTTCAGCAACACGGAATG | |  |

**Supplementary Table S3.** **NCBI accession numbers of the bacterial isolates.**

| Isolate | Accession Number |
| --- | --- |
| *Bacillus* sp. isolate H1 | KU740213-KU740214 |
| *Bacillus* sp. isolate H2 | KU740215-KU740216 |
| *Staphylococcus* sp. isolate H3 | KU740217-KU740218 |
| *Staphylococcus* sp. isolate H4 | KU740219-KU740220 |
| *Bacillus* sp. isolate H9 | KU740221-KU740222 |
| *Bacillus* sp. isolate SB3 | KU740223-KU740224 |
| *Bacillus* sp. isolate SB6 | KU740225-KU740226 |
| *Bacillus* sp. isolate SB12 | KU740227-KU740228 |
| *Bacillus* sp. isolate SB13 | KU740229-KU740230 |
| *Bacillus* sp. isolate SB16 | KU740231-KU740232 |
| *Bacillus* sp. isolate SW3 | KU740233-KU740234 |
| *Bacillus* sp. isolate SW7 | KU740235-KU740236 |
| *Escherichia* sp. isolate SW11 | KU740237-KU740238 |

**Supplementary Table S4. Complete pH data over 96 hrs from each of the media combinations for each of the bacterial isolates used in the experiments.**

| Name of the organisms | Culture conditions | | pH | | | | | |
| --- | --- | --- | --- | --- | --- | --- | --- | --- |
|  | PO_4_^3-^-P in ppm | NO_3_^-^-N in ppm | 0 hour | 12 hours | 24 hours | 48 hours | 72 hours | 96 hours |
| *Escherichia coli* K12 ER2925 | 0 | 0 | 7.0 | 7.2 | 6.9 | 6.7 | 7.2 | 6.9 |
|  |  | 50 | 7.0 | 7.2 | 7.4 | 7.5 | 7.5 | 7.2 |
|  |  | 100 | 7.0 | 7.3 | 7.4 | 7.5 | 7.5 | 7.3 |
|  |  | 500 | 7.0 | 7.5 | 7.5 | 7.5 | 7.5 | 7.5 |
|  |  | 1000 | 7.0 | 7.3 | 7.4 | 7.5 | 7.5 | 7.3 |
|  |  | 2000 | 7.0 | 7.6 | 7.6 | 7.5 | 7.5 | 7.6 |
|  | 10 | 0 | 7.0 | 7.1 | 7.0 | 6.9 | 7.1 | 6.9 |
|  |  | 50 | 7.0 | 7.2 | 7.2 | 7.6 | 7.6 | 7.2 |
|  |  | 100 | 7.0 | 7.2 | 7.4 | 7.5 | 7.5 | 7.3 |
|  |  | 500 | 7.0 | 7.2 | 7.4 | 7.5 | 7.5 | 7.4 |
|  |  | 1000 | 7.0 | 7.3 | 7.3 | 7.6 | 7.6 | 7.4 |
|  |  | 2000 | 7.0 | 7.2 | 7.4 | 7.6 | 7.6 | 7.4 |
|  | 50 | 0 | 7.0 | 6.9 | 7.2 | 7.1 | 7.0 | 7.1 |
|  |  | 50 | 7.0 | 7.2 | 7.3 | 7.5 | 7.4 | 7.3 |
|  |  | 100 | 7.0 | 7.3 | 7.4 | 7.5 | 7.5 | 7.4 |
|  |  | 500 | 7.0 | 7.5 | 7.4 | 7.5 | 7.6 | 7.4 |
|  |  | 1000 | 7.0 | 7.3 | 7.4 | 7.5 | 7.5 | 7.4 |
|  |  | 2000 | 7.0 | 7.6 | 7.4 | 7.5 | 7.6 | 7.5 |
|  | 100 | 0 | 7.0 | 7.0 | 6.9 | 7.0 | 7.0 | 7.2 |
|  |  | 50 | 7.0 | 7.2 | 7.2 | 7.6 | 7.5 | 7.2 |
|  |  | 100 | 7.0 | 7.3 | 7.3 | 7.5 | 7.4 | 7.3 |
|  |  | 500 | 7.0 | 7.4 | 7.5 | 7.5 | 7.6 | 7.5 |
|  |  | 1000 | 7.0 | 7.1 | 7.3 | 7.6 | 7.6 | 7.3 |
|  |  | 2000 | 7.0 | 7.2 | 7.6 | 7.6 | 7.5 | 7.6 |
|  | 250 | 0 | 7.0 | 7.0 | 7.1 | 7.0 | 7.0 | 6.9 |
|  |  | 50 | 7.0 | 7.2 | 7.2 | 7.4 | 7.5 | 7.3 |
|  |  | 100 | 7.0 | 7.2 | 7.3 | 7.5 | 7.5 | 7.4 |
|  |  | 500 | 7.0 | 7.2 | 7.5 | 7.6 | 7.5 | 7.5 |
|  |  | 1000 | 7.0 | 7.2 | 7.3 | 7.5 | 7.5 | 7.4 |
|  |  | 2000 | 7.0 | 7.2 | 7.6 | 7.6 | 7.5 | 7.3 |
|  | 500 | 0 | 7.0 | 7.0 | 7.1 | 7.0 | 7.0 | 6.9 |
|  |  | 50 | 7.0 | 7.2 | 7.3 | 7.5 | 7.5 | 7.2 |
|  |  | 100 | 7.0 | 7.3 | 7.4 | 7.5 | 7.4 | 7.2 |
|  |  | 500 | 7.0 | 7.5 | 7.5 | 7.5 | 7.6 | 7.3 |
|  |  | 1000 | 7.0 | 7.3 | 7.4 | 7.5 | 7.6 | 7.3 |
|  |  | 2000 | 7.0 | 7.6 | 7.3 | 7.5 | 7.5 | 7.3 |
| *Escherichia coli* K12 PR1031 | 0 | 0 | 7.0 | 7.1 | 7.2 | 7.0 | 7.0 | 6.9 |
|  |  | 50 | 7.0 | 7.2 | 7.2 | 7.5 | 7.5 | 7.2 |
|  |  | 100 | 7.0 | 7.3 | 7.3 | 7.5 | 7.5 | 7.3 |
|  |  | 500 | 7.0 | 7.3 | 7.5 | 7.5 | 7.5 | 7.5 |
|  |  | 1000 | 7.0 | 7.3 | 7.3 | 7.5 | 7.5 | 7.3 |
|  |  | 2000 | 7.0 | 7.2 | 7.6 | 7.5 | 7.5 | 7.6 |
|  | 10 | 0 | 7.0 | 7.0 | 7.1 | 7.0 | 7.0 | 6.9 |
|  |  | 50 | 7.0 | 7.3 | 7.2 | 7.6 | 7.6 | 7.2 |
|  |  | 100 | 7.0 | 7.5 | 7.2 | 7.5 | 7.5 | 7.3 |
|  |  | 500 | 7.0 | 7.6 | 7.2 | 7.5 | 7.5 | 7.3 |
|  |  | 1000 | 7.0 | 7.4 | 7.3 | 7.6 | 7.6 | 7.3 |
|  |  | 2000 | 7.0 | 7.4 | 7.2 | 7.6 | 7.6 | 7.4 |
|  | 50 | 0 | 7.0 | 7.1 | 6.9 | 7.0 | 7.1 | 7.0 |
|  |  | 50 | 7.0 | 7.3 | 7.2 | 7.4 | 7.4 | 7.1 |
|  |  | 100 | 7.0 | 7.4 | 7.3 | 7.5 | 7.5 | 7.3 |
|  |  | 500 | 7.0 | 7.2 | 7.5 | 7.6 | 7.6 | 7.3 |
|  |  | 1000 | 7.0 | 7.2 | 7.3 | 7.5 | 7.5 | 7.5 |
|  |  | 2000 | 7.0 | 7.2 | 7.6 | 7.6 | 7.6 | 7.4 |
|  | 100 | 0 | 7.0 | 7.0 | 7.0 | 7.1 | 7.0 | 7.2 |
|  |  | 50 | 7.0 | 7.2 | 7.2 | 7.6 | 7.5 | 7.2 |
|  |  | 100 | 7.0 | 7.3 | 7.3 | 7.5 | 7.4 | 7.3 |
|  |  | 500 | 7.0 | 7.5 | 7.4 | 7.5 | 7.6 | 7.5 |
|  |  | 1000 | 7.0 | 7.4 | 7.1 | 7.6 | 7.6 | 7.3 |
|  |  | 2000 | 7.0 | 7.6 | 7.2 | 7.6 | 7.5 | 7.6 |
|  | 250 | 0 | 7.0 | 7.2 | 7.0 | 7.1 | 7.1 | 6.9 |
|  |  | 50 | 7.0 | 7.2 | 7.2 | 7.5 | 7.5 | 7.2 |
|  |  | 100 | 7.0 | 7.3 | 7.2 | 7.5 | 7.5 | 7.3 |
|  |  | 500 | 7.0 | 7.5 | 7.2 | 7.5 | 7.5 | 7.5 |
|  |  | 1000 | 7.0 | 7.3 | 7.2 | 7.5 | 7.5 | 7.3 |
|  |  | 2000 | 7.0 | 7.6 | 7.2 | 7.5 | 7.5 | 7.6 |
|  | 500 | 0 | 7.0 | 7.0 | 7.0 | 6.9 | 7.0 | 7.1 |
|  |  | 50 | 7.0 | 7.2 | 7.2 | 7.5 | 7.5 | 7.2 |
|  |  | 100 | 7.0 | 7.3 | 7.3 | 7.5 | 7.4 | 7.3 |
|  |  | 500 | 7.0 | 7.4 | 7.5 | 7.5 | 7.6 | 7.3 |
|  |  | 1000 | 7.0 | 7.5 | 7.3 | 7.5 | 7.6 | 7.4 |
|  |  | 2000 | 7.0 | 7.3 | 7.6 | 7.5 | 7.5 | 7.5 |
| *Escherichia coli* DH5α | 0 | 0 | 7.0 | 6.9 | 7.1 | 7.0 | 7.0 | 7.1 |
|  |  | 50 | 7.0 | 7.2 | 7.2 | 7.5 | 7.5 | 7.2 |
|  |  | 100 | 7.0 | 7.3 | 7.3 | 7.5 | 7.5 | 7.3 |
|  |  | 500 | 7.0 | 7.5 | 7.3 | 7.5 | 7.5 | 7.5 |
|  |  | 1000 | 7.0 | 7.3 | 7.3 | 7.5 | 7.5 | 7.3 |
|  |  | 2000 | 7.0 | 7.6 | 7.2 | 7.5 | 7.5 | 7.6 |
|  | 10 | 0 | 7.0 | 6.9 | 7.0 | 7.0 | 7.0 | 7.1 |
|  |  | 50 | 7.0 | 7.2 | 7.3 | 7.6 | 7.6 | 7.2 |
|  |  | 100 | 7.0 | 7.4 | 7.5 | 7.5 | 7.5 | 7.3 |
|  |  | 500 | 7.0 | 7.3 | 7.6 | 7.5 | 7.5 | 7.5 |
|  |  | 1000 | 7.0 | 7.3 | 7.4 | 7.6 | 7.6 | 7.3 |
|  |  | 2000 | 7.0 | 7.3 | 7.4 | 7.6 | 7.6 | 7.6 |
|  | 50 | 0 | 7.0 | 7.1 | 7.1 | 6.9 | 7.1 | 7.1 |
|  |  | 50 | 7.0 | 7.3 | 7.3 | 7.4 | 7.4 | 7.2 |
|  |  | 100 | 7.0 | 7.3 | 7.4 | 7.5 | 7.5 | 7.3 |
|  |  | 500 | 7.0 | 7.3 | 7.2 | 7.6 | 7.6 | 7.5 |
|  |  | 1000 | 7.0 | 7.3 | 7.2 | 7.5 | 7.5 | 7.3 |
|  |  | 2000 | 7.0 | 7.4 | 7.2 | 7.6 | 7.6 | 7.6 |
|  | 100 | 0 | 7.0 | 6.9 | 7.0 | 7.2 | 6.9 | 6.9 |
|  |  | 50 | 7.0 | 7.3 | 7.2 | 7.6 | 7.5 | 7.2 |
|  |  | 100 | 7.0 | 7.4 | 7.3 | 7.5 | 7.5 | 7.4 |
|  |  | 500 | 7.0 | 7.4 | 7.5 | 7.5 | 7.5 | 7.5 |
|  |  | 1000 | 7.0 | 7.3 | 7.4 | 7.6 | 7.5 | 7.4 |
|  |  | 2000 | 7.0 | 7.4 | 7.6 | 7.6 | 7.5 | 7.4 |
|  | 250 | 0 | 7.0 | 7.1 | 7.2 | 7.1 | 7.1 | 7.0 |
|  |  | 50 | 7.0 | 7.2 | 7.2 | 7.4 | 7.5 | 7.2 |
|  |  | 100 | 7.0 | 7.3 | 7.3 | 7.5 | 7.4 | 7.4 |
|  |  | 500 | 7.0 | 7.5 | 7.5 | 7.6 | 7.6 | 7.4 |
|  |  | 1000 | 7.0 | 7.3 | 7.3 | 7.5 | 7.6 | 7.4 |
|  |  | 2000 | 7.0 | 7.6 | 7.6 | 7.6 | 7.5 | 7.4 |
|  | 500 | 0 | 7.0 | 6.9 | 7.0 | 7.1 | 7.0 | 7.1 |
|  |  | 50 | 7.0 | 7.2 | 7.2 | 7.6 | 7.5 | 7.2 |
|  |  | 100 | 7.0 | 7.3 | 7.3 | 7.5 | 7.5 | 7.3 |
|  |  | 500 | 7.0 | 7.4 | 7.4 | 7.5 | 7.5 | 7.5 |
|  |  | 1000 | 7.0 | 7.5 | 7.5 | 7.6 | 7.5 | 7.3 |
|  |  | 2000 | 7.0 | 7.4 | 7.3 | 7.6 | 7.5 | 7.6 |
| *Bacillus* sp.isolate H1 | 0 | 0 | 7.0 | 6.9 | 7.2 | 7.2 | 7.0 | 7.2 |
|  |  | 50 | 7.0 | 7.2 | 7.2 | 7.5 | 7.5 | 7.2 |
|  |  | 100 | 7.0 | 7.3 | 7.3 | 7.5 | 7.5 | 7.3 |
|  |  | 500 | 7.0 | 7.5 | 7.5 | 7.5 | 7.5 | 7.5 |
|  |  | 1000 | 7.0 | 7.3 | 7.3 | 7.5 | 7.5 | 7.3 |
|  |  | 2000 | 7.0 | 7.6 | 7.6 | 7.5 | 7.5 | 7.6 |
|  | 10 | 0 | 7.0 | 7.0 | 7.1 | 7.0 | 6.9 | 7.1 |
|  |  | 50 | 7.0 | 7.2 | 7.2 | 7.6 | 7.6 | 7.2 |
|  |  | 100 | 7.0 | 7.4 | 7.2 | 7.5 | 7.5 | 7.2 |
|  |  | 500 | 7.0 | 7.4 | 7.2 | 7.5 | 7.5 | 7.2 |
|  |  | 1000 | 7.0 | 7.3 | 7.3 | 7.6 | 7.6 | 7.3 |
|  |  | 2000 | 7.0 | 7.4 | 7.2 | 7.6 | 7.6 | 7.2 |
|  | 50 | 0 | 7.0 | 7.2 | 6.9 | 7.0 | 7.1 | 6.9 |
|  |  | 50 | 7.0 | 7.3 | 7.2 | 7.6 | 7.5 | 7.2 |
|  |  | 100 | 7.0 | 7.4 | 7.3 | 7.5 | 7.5 | 7.3 |
|  |  | 500 | 7.0 | 7.4 | 7.5 | 7.5 | 7.5 | 7.5 |
|  |  | 1000 | 7.0 | 7.4 | 7.3 | 7.6 | 7.5 | 7.3 |
|  |  | 2000 | 7.0 | 7.4 | 7.6 | 7.6 | 7.5 | 7.6 |
|  | 100 | 0 | 7.0 | 6.9 | 7.0 | 7.0 | 7.0 | 7.0 |
|  |  | 50 | 7.0 | 7.2 | 7.2 | 7.4 | 7.6 | 7.2 |
|  |  | 100 | 7.0 | 7.3 | 7.3 | 7.5 | 7.5 | 7.3 |
|  |  | 500 | 7.0 | 7.5 | 7.4 | 7.6 | 7.5 | 7.4 |
|  |  | 1000 | 7.0 | 7.3 | 7.1 | 7.5 | 7.6 | 7.1 |
|  |  | 2000 | 7.0 | 7.6 | 7.2 | 7.6 | 7.6 | 7.2 |
|  | 250 | 0 | 7.0 | 7.1 | 7.0 | 7.0 | 7.0 | 7.0 |
|  |  | 50 | 7.0 | 7.2 | 7.2 | 7.5 | 7.4 | 7.2 |
|  |  | 100 | 7.0 | 7.3 | 7.2 | 7.5 | 7.5 | 7.2 |
|  |  | 500 | 7.0 | 7.5 | 7.2 | 7.5 | 7.6 | 7.2 |
|  |  | 1000 | 7.0 | 7.3 | 7.2 | 7.5 | 7.5 | 7.2 |
|  |  | 2000 | 7.0 | 7.6 | 7.2 | 7.5 | 7.6 | 7.2 |
|  | 500 | 0 | 7.0 | 7.1 | 7.0 | 7.0 | 7.0 | 7.0 |
|  |  | 50 | 7.0 | 7.3 | 7.2 | 7.4 | 7.5 | 7.2 |
|  |  | 100 | 7.0 | 7.4 | 7.3 | 7.5 | 7.5 | 7.3 |
|  |  | 500 | 7.0 | 7.5 | 7.5 | 7.6 | 7.5 | 7.5 |
|  |  | 1000 | 7.0 | 7.4 | 7.3 | 7.5 | 7.5 | 7.3 |
|  |  | 2000 | 7.0 | 7.3 | 7.6 | 7.5 | 7.5 | 7.6 |
| *Bacillus* sp.isolate H2 | 0 | 0 | 7.0 | 7.2 | 7.1 | 7.0 | 7.0 | 7.2 |
|  |  | 50 | 7.0 | 7.2 | 7.2 | 7.6 | 7.5 | 7.2 |
|  |  | 100 | 7.0 | 7.3 | 7.3 | 7.5 | 7.5 | 7.3 |
|  |  | 500 | 7.0 | 7.5 | 7.3 | 7.5 | 7.5 | 7.5 |
|  |  | 1000 | 7.0 | 7.3 | 7.3 | 7.6 | 7.5 | 7.3 |
|  |  | 2000 | 7.0 | 7.6 | 7.2 | 7.6 | 7.5 | 7.6 |
|  | 10 | 0 | 7.0 | 7.1 | 7.0 | 7.0 | 7.0 | 7.1 |
|  |  | 50 | 7.0 | 7.2 | 7.3 | 7.4 | 7.6 | 7.2 |
|  |  | 100 | 7.0 | 7.2 | 7.5 | 7.5 | 7.5 | 7.2 |
|  |  | 500 | 7.0 | 7.2 | 7.6 | 7.6 | 7.5 | 7.2 |
|  |  | 1000 | 7.0 | 7.3 | 7.4 | 7.5 | 7.6 | 7.3 |
|  |  | 2000 | 7.0 | 7.2 | 7.4 | 7.6 | 7.6 | 7.2 |
|  | 50 | 0 | 7.0 | 6.9 | 7.1 | 7.5 | 7.0 | 6.9 |
|  |  | 50 | 7.0 | 7.2 | 7.3 | 7.5 | 7.4 | 7.2 |
|  |  | 100 | 7.0 | 7.3 | 7.4 | 7.5 | 7.5 | 7.3 |
|  |  | 500 | 7.0 | 7.5 | 7.2 | 7.5 | 7.6 | 7.5 |
|  |  | 1000 | 7.0 | 7.3 | 7.2 | 7.5 | 7.5 | 7.3 |
|  |  | 2000 | 7.0 | 7.6 | 7.2 | 7.5 | 7.6 | 7.6 |
|  | 100 | 0 | 7.0 | 7.0 | 7.0 | 7.0 | 7.1 | 7.0 |
|  |  | 50 | 7.0 | 7.2 | 7.2 | 7.5 | 7.6 | 7.2 |
|  |  | 100 | 7.0 | 7.3 | 7.3 | 7.5 | 7.5 | 7.3 |
|  |  | 500 | 7.0 | 7.4 | 7.5 | 7.5 | 7.5 | 7.4 |
|  |  | 1000 | 7.0 | 7.1 | 7.4 | 7.5 | 7.6 | 7.1 |
|  |  | 2000 | 7.0 | 7.2 | 7.6 | 7.5 | 7.6 | 7.2 |
|  | 250 | 0 | 7.0 | 7.0 | 7.2 | 7.2 | 7.1 | 7.0 |
|  |  | 50 | 7.0 | 7.2 | 7.2 | 7.6 | 7.5 | 7.2 |
|  |  | 100 | 7.0 | 7.2 | 7.3 | 7.5 | 7.5 | 7.2 |
|  |  | 500 | 7.0 | 7.2 | 7.5 | 7.5 | 7.5 | 7.2 |
|  |  | 1000 | 7.0 | 7.2 | 7.3 | 7.6 | 7.5 | 7.2 |
|  |  | 2000 | 7.0 | 7.2 | 7.6 | 7.6 | 7.5 | 7.2 |
|  | 500 | 0 | 7.0 | 7.0 | 7.0 | 7.0 | 6.9 | 7.0 |
|  |  | 50 | 7.0 | 7.2 | 7.2 | 7.4 | 7.5 | 7.2 |
|  |  | 100 | 7.0 | 7.3 | 7.3 | 7.5 | 7.5 | 7.3 |
|  |  | 500 | 7.0 | 7.5 | 7.4 | 7.6 | 7.5 | 7.5 |
|  |  | 1000 | 7.0 | 7.3 | 7.5 | 7.5 | 7.5 | 7.3 |
|  |  | 2000 | 7.0 | 7.6 | 7.3 | 7.6 | 7.5 | 7.6 |
| *Staphylococcus* sp.isolate H3 | 0 | 0 | 7.0 | 7.1 | 6.9 | 7.0 | 7.0 | 7.1 |
|  |  | 50 | 7.0 | 7.2 | 7.2 | 7.4 | 7.5 | 7.2 |
|  |  | 100 | 7.0 | 7.3 | 7.3 | 7.5 | 7.5 | 7.3 |
|  |  | 500 | 7.0 | 7.3 | 7.5 | 7.6 | 7.5 | 7.3 |
|  |  | 1000 | 7.0 | 7.3 | 7.3 | 7.5 | 7.5 | 7.3 |
|  |  | 2000 | 7.0 | 7.2 | 7.6 | 7.5 | 7.5 | 7.2 |
|  | 10 | 0 | 7.0 | 7.0 | 6.9 | 7.0 | 7.0 | 7.0 |
|  |  | 50 | 7.0 | 7.3 | 7.2 | 7.5 | 7.6 | 7.3 |
|  |  | 100 | 7.0 | 7.5 | 7.4 | 7.5 | 7.5 | 7.5 |
|  |  | 500 | 7.0 | 7.6 | 7.3 | 7.5 | 7.5 | 7.6 |
|  |  | 1000 | 7.0 | 7.4 | 7.3 | 7.5 | 7.6 | 7.4 |
|  |  | 2000 | 7.0 | 7.4 | 7.3 | 7.5 | 7.6 | 7.4 |
|  | 50 | 0 | 7.0 | 7.1 | 7.1 | 7.0 | 6.9 | 7.1 |
|  |  | 50 | 7.0 | 7.3 | 7.3 | 7.4 | 7.4 | 7.3 |
|  |  | 100 | 7.0 | 7.4 | 7.3 | 7.5 | 7.5 | 7.4 |
|  |  | 500 | 7.0 | 7.2 | 7.3 | 7.6 | 7.6 | 7.2 |
|  |  | 1000 | 7.0 | 7.2 | 7.3 | 7.5 | 7.5 | 7.2 |
|  |  | 2000 | 7.0 | 7.2 | 7.4 | 7.5 | 7.6 | 7.2 |
|  | 100 | 0 | 7.0 | 7.0 | 6.9 | 7.0 | 7.2 | 7.0 |
|  |  | 50 | 7.0 | 7.2 | 7.3 | 7.4 | 7.6 | 7.2 |
|  |  | 100 | 7.0 | 7.3 | 7.4 | 7.5 | 7.5 | 7.3 |
|  |  | 500 | 7.0 | 7.5 | 7.4 | 7.6 | 7.5 | 7.5 |
|  |  | 1000 | 7.0 | 7.4 | 7.3 | 7.5 | 7.6 | 7.4 |
|  |  | 2000 | 7.0 | 7.6 | 7.4 | 7.5 | 7.6 | 7.6 |
|  | 250 | 0 | 7.0 | 7.2 | 7.1 | 7.0 | 7.1 | 7.2 |
|  |  | 50 | 7.0 | 7.2 | 7.2 | 7.5 | 7.4 | 7.2 |
|  |  | 100 | 7.0 | 7.3 | 7.3 | 7.5 | 7.5 | 7.3 |
|  |  | 500 | 7.0 | 7.5 | 7.5 | 7.5 | 7.6 | 7.5 |
|  |  | 1000 | 7.0 | 7.3 | 7.3 | 7.5 | 7.5 | 7.3 |
|  |  | 2000 | 7.0 | 7.6 | 7.6 | 7.5 | 7.6 | 7.6 |
|  | 500 | 0 | 7.0 | 7.0 | 6.9 | 7.0 | 7.1 | 7.0 |
|  |  | 50 | 7.0 | 7.2 | 7.2 | 7.6 | 7.6 | 7.2 |
|  |  | 100 | 7.0 | 7.3 | 7.3 | 7.5 | 7.5 | 7.3 |
|  |  | 500 | 7.0 | 7.4 | 7.4 | 7.5 | 7.5 | 7.4 |
|  |  | 1000 | 7.0 | 7.5 | 7.5 | 7.6 | 7.6 | 7.5 |
|  |  | 2000 | 7.0 | 7.3 | 7.4 | 7.6 | 7.6 | 7.3 |
| *Staphylococcus* sp.isolate H4 | 0 | 0 | 7.0 | 7.1 | 6.9 | 7.0 | 7.2 | 7.2 |
|  |  | 50 | 7.0 | 7.2 | 7.2 | 7.4 | 7.5 | 7.2 |
|  |  | 100 | 7.0 | 7.3 | 7.3 | 7.5 | 7.5 | 7.3 |
|  |  | 500 | 7.0 | 7.5 | 7.5 | 7.6 | 7.5 | 7.5 |
|  |  | 1000 | 7.0 | 7.3 | 7.3 | 7.5 | 7.5 | 7.3 |
|  |  | 2000 | 7.0 | 7.6 | 7.6 | 7.6 | 7.5 | 7.6 |
|  | 10 | 0 | 7.0 | 7.1 | 7.0 | 7.0 | 7.1 | 7.1 |
|  |  | 50 | 7.0 | 7.2 | 7.2 | 7.5 | 7.6 | 7.2 |
|  |  | 100 | 7.0 | 7.3 | 7.4 | 7.5 | 7.5 | 7.2 |
|  |  | 500 | 7.0 | 7.5 | 7.4 | 7.5 | 7.5 | 7.2 |
|  |  | 1000 | 7.0 | 7.3 | 7.3 | 7.5 | 7.6 | 7.3 |
|  |  | 2000 | 7.0 | 7.6 | 7.4 | 7.5 | 7.6 | 7.2 |
|  | 50 | 0 | 7.0 | 7.2 | 7.2 | 7.0 | 7.0 | 6.9 |
|  |  | 50 | 7.0 | 7.2 | 7.3 | 7.5 | 7.4 | 7.2 |
|  |  | 100 | 7.0 | 7.3 | 7.4 | 7.5 | 7.5 | 7.3 |
|  |  | 500 | 7.0 | 7.5 | 7.4 | 7.5 | 7.6 | 7.5 |
|  |  | 1000 | 7.0 | 7.3 | 7.4 | 7.5 | 7.5 | 7.3 |
|  |  | 2000 | 7.0 | 7.6 | 7.4 | 7.5 | 7.6 | 7.6 |
|  | 100 | 0 | 7.0 | 6.9 | 6.9 | 7.0 | 7.0 | 7.0 |
|  |  | 50 | 7.0 | 7.2 | 7.2 | 7.5 | 7.5 | 7.2 |
|  |  | 100 | 7.0 | 7.4 | 7.3 | 7.5 | 7.4 | 7.3 |
|  |  | 500 | 7.0 | 7.5 | 7.5 | 7.5 | 7.6 | 7.4 |
|  |  | 1000 | 7.0 | 7.4 | 7.3 | 7.5 | 7.6 | 7.1 |
|  |  | 2000 | 7.0 | 7.4 | 7.6 | 7.5 | 7.5 | 7.2 |
|  | 250 | 0 | 7.0 | 7.0 | 7.1 | 7.1 | 7.0 | 7.0 |
|  |  | 50 | 7.0 | 7.2 | 7.2 | 7.5 | 7.5 | 7.2 |
|  |  | 100 | 7.0 | 7.4 | 7.3 | 7.5 | 7.5 | 7.2 |
|  |  | 500 | 7.0 | 7.4 | 7.5 | 7.5 | 7.5 | 7.2 |
|  |  | 1000 | 7.0 | 7.4 | 7.3 | 7.5 | 7.5 | 7.2 |
|  |  | 2000 | 7.0 | 7.4 | 7.6 | 7.5 | 7.5 | 7.2 |
|  | 500 | 0 | 7.0 | 7.1 | 7.1 | 7.1 | 7.0 | 7.0 |
|  |  | 50 | 7.0 | 7.2 | 7.3 | 7.5 | 7.5 | 7.2 |
|  |  | 100 | 7.0 | 7.3 | 7.4 | 7.5 | 7.4 | 7.3 |
|  |  | 500 | 7.0 | 7.5 | 7.5 | 7.5 | 7.6 | 7.5 |
|  |  | 1000 | 7.0 | 7.3 | 7.4 | 7.5 | 7.6 | 7.3 |
|  |  | 2000 | 7.0 | 7.6 | 7.3 | 7.5 | 7.5 | 7.6 |
| *Bacillus* sp.isolate H9 | 0 | 0 | 7.0 | 7.23 | 7.2 | 7.2 | 7.0 | 6.9 |
|  |  | 50 | 7.0 | 7.2 | 7.2 | 7.5 | 7.5 | 7.4 |
|  |  | 100 | 7.0 | 7.3 | 7.3 | 7.5 | 7.5 | 7.4 |
|  |  | 500 | 7.0 | 7.5 | 7.5 | 7.5 | 7.5 | 7.5 |
|  |  | 1000 | 7.0 | 7.3 | 7.3 | 7.5 | 7.5 | 7.4 |
|  |  | 2000 | 7.0 | 7.6 | 7.6 | 7.5 | 7.5 | 7.6 |
|  | 10 | 0 | 7.0 | 7.1 | 7.1 | 7.1 | 7.0 | 7.0 |
|  |  | 50 | 7.0 | 7.2 | 7.2 | 7.6 | 7.6 | 7.2 |
|  |  | 100 | 7.0 | 7.2 | 7.2 | 7.5 | 7.5 | 7.4 |
|  |  | 500 | 7.0 | 7.2 | 7.2 | 7.5 | 7.5 | 7.4 |
|  |  | 1000 | 7.0 | 7.3 | 7.3 | 7.6 | 7.6 | 7.3 |
|  |  | 2000 | 7.0 | 7.2 | 7.2 | 7.6 | 7.6 | 7.4 |
|  | 50 | 0 | 7.0 | 6.9 | 6.9 | 6.9 | 7.1 | 7.2 |
|  |  | 50 | 7.0 | 7.2 | 7.2 | 7.4 | 7.4 | 7.3 |
|  |  | 100 | 7.0 | 7.3 | 7.3 | 7.5 | 7.5 | 7.4 |
|  |  | 500 | 7.0 | 7.5 | 7.5 | 7.6 | 7.6 | 7.4 |
|  |  | 1000 | 7.0 | 7.3 | 7.3 | 7.5 | 7.5 | 7.4 |
|  |  | 2000 | 7.0 | 7.6 | 7.6 | 7.6 | 7.6 | 7.4 |
|  | 100 | 0 | 7.0 | 7.0 | 7.0 | 7.0 | 7.0 | 6.9 |
|  |  | 50 | 7.0 | 7.2 | 7.2 | 7.5 | 7.5 | 7.2 |
|  |  | 100 | 7.0 | 7.3 | 7.3 | 7.4 | 7.4 | 7.3 |
|  |  | 500 | 7.0 | 7.4 | 7.4 | 7.6 | 7.6 | 7.5 |
|  |  | 1000 | 7.0 | 7.1 | 7.1 | 7.6 | 7.6 | 7.3 |
|  |  | 2000 | 7.0 | 7.2 | 7.2 | 7.5 | 7.5 | 7.6 |
|  | 250 | 0 | 7.0 | 7.0 | 7.0 | 7.0 | 7.1 | 7.1 |
|  |  | 50 | 7.0 | 7.2 | 7.2 | 7.6 | 7.5 | 7.2 |
|  |  | 100 | 7.0 | 7.2 | 7.2 | 7.5 | 7.5 | 7.3 |
|  |  | 500 | 7.0 | 7.2 | 7.2 | 7.5 | 7.5 | 7.5 |
|  |  | 1000 | 7.0 | 7.2 | 7.2 | 7.6 | 7.5 | 7.3 |
|  |  | 2000 | 7.0 | 7.2 | 7.2 | 7.6 | 7.5 | 7.6 |
|  | 500 | 0 | 7.0 | 7.0 | 7.0 | 7.0 | 7.0 | 7.1 |
|  |  | 50 | 7.0 | 7.2 | 7.2 | 7.4 | 7.5 | 7.3 |
|  |  | 100 | 7.0 | 7.3 | 7.3 | 7.5 | 7.4 | 7.4 |
|  |  | 500 | 7.0 | 7.5 | 7.5 | 7.6 | 7.6 | 7.5 |
|  |  | 1000 | 7.0 | 7.3 | 7.3 | 7.5 | 7.6 | 7.4 |
|  |  | 2000 | 7.0 | 7.6 | 7.6 | 7.6 | 7.5 | 7.3 |
| *Bacillus* sp.isolate SB3 | 0 | 0 | 7.0 | 7.1 | 7.1 | 7.1 | 7.0 | 7.2 |
|  |  | 50 | 7.0 | 7.2 | 7.2 | 7.5 | 7.5 | 7.2 |
|  |  | 100 | 7.0 | 7.3 | 7.3 | 7.5 | 7.5 | 7.3 |
|  |  | 500 | 7.0 | 7.3 | 7.3 | 7.5 | 7.5 | 7.5 |
|  |  | 1000 | 7.0 | 7.3 | 7.3 | 7.5 | 7.5 | 7.3 |
|  |  | 2000 | 7.0 | 7.2 | 7.2 | 7.5 | 7.5 | 7.6 |
|  | 10 | 0 | 7.0 | 7.0 | 7.0 | 7.1 | 7.0 | 7.1 |
|  |  | 50 | 7.0 | 7.3 | 7.3 | 7.5 | 7.6 | 7.2 |
|  |  | 100 | 7.0 | 7.5 | 7.5 | 7.4 | 7.5 | 7.2 |
|  |  | 500 | 7.0 | 7.6 | 7.6 | 7.6 | 7.5 | 7.2 |
|  |  | 1000 | 7.0 | 7.4 | 7.4 | 7.6 | 7.6 | 7.3 |
|  |  | 2000 | 7.0 | 7.4 | 7.4 | 7.5 | 7.6 | 7.2 |
|  | 50 | 0 | 7.0 | 7.1 | 7.1 | 7.1 | 7.1 | 6.9 |
|  |  | 50 | 7.0 | 7.3 | 7.3 | 7.5 | 7.4 | 7.2 |
|  |  | 100 | 7.0 | 7.4 | 7.4 | 7.5 | 7.5 | 7.3 |
|  |  | 500 | 7.0 | 7.2 | 7.2 | 7.5 | 7.6 | 7.5 |
|  |  | 1000 | 7.0 | 7.2 | 7.2 | 7.5 | 7.5 | 7.3 |
|  |  | 2000 | 7.0 | 7.2 | 7.2 | 7.6 | 7.6 | 7.6 |
|  | 100 | 0 | 7.0 | 7.0 | 7.0 | 7.0 | 6.9 | 7.0 |
|  |  | 50 | 7.0 | 7.2 | 7.2 | 7.5 | 7.5 | 7.2 |
|  |  | 100 | 7.0 | 7.3 | 7.3 | 7.4 | 7.5 | 7.3 |
|  |  | 500 | 7.0 | 7.5 | 7.5 | 7.6 | 7.5 | 7.4 |
|  |  | 1000 | 7.0 | 7.4 | 7.4 | 7.6 | 7.5 | 7.1 |
|  |  | 2000 | 7.0 | 7.6 | 7.6 | 7.5 | 7.5 | 7.2 |
|  | 250 | 0 | 7.0 | 7.2 | 7.2 | 7.2 | 7.1 | 7.0 |
|  |  | 50 | 7.0 | 7.2 | 7.2 | 7.5 | 7.5 | 7.2 |
|  |  | 100 | 7.0 | 7.3 | 7.3 | 7.5 | 7.4 | 7.2 |
|  |  | 500 | 7.0 | 7.5 | 7.5 | 7.5 | 7.6 | 7.2 |
|  |  | 1000 | 7.0 | 7.3 | 7.3 | 7.5 | 7.6 | 7.2 |
|  |  | 2000 | 7.0 | 7.6 | 7.6 | 7.5 | 7.5 | 7.2 |
|  | 500 | 0 | 7.0 | 7.0 | 7.0 | 7.0 | 7.0 | 7.0 |
|  |  | 50 | 7.0 | 7.2 | 7.2 | 7.5 | 7.5 | 7.2 |
|  |  | 100 | 7.0 | 7.3 | 7.3 | 7.5 | 7.5 | 7.3 |
|  |  | 500 | 7.0 | 7.4 | 7.4 | 7.5 | 7.5 | 7.5 |
|  |  | 1000 | 7.0 | 7.5 | 7.5 | 7.5 | 7.5 | 7.3 |
|  |  | 2000 | 7.0 | 7.3 | 7.3 | 7.5 | 7.5 | 7.6 |
| *Bacillus* sp.isolate SB6 | 0 | 0 | 7.0 | 6.9 | 7.1 | 7.0 | 6.7 | 7.1 |
|  |  | 50 | 7.0 | 7.2 | 7.2 | 7.5 | 7.5 | 7.2 |
|  |  | 100 | 7.0 | 7.3 | 7.3 | 7.5 | 7.5 | 7.3 |
|  |  | 500 | 7.0 | 7.5 | 7.3 | 7.5 | 7.5 | 7.3 |
|  |  | 1000 | 7.0 | 7.3 | 7.3 | 7.5 | 7.5 | 7.3 |
|  |  | 2000 | 7.0 | 7.6 | 7.2 | 7.5 | 7.5 | 7.2 |
|  | 10 | 0 | 7.0 | 6.9 | 7.0 | 7.0 | 6.9 | 7.0 |
|  |  | 50 | 7.0 | 7.2 | 7.3 | 7.6 | 7.6 | 7.3 |
|  |  | 100 | 7.0 | 7.3 | 7.5 | 7.5 | 7.5 | 7.5 |
|  |  | 500 | 7.0 | 7.4 | 7.6 | 7.5 | 7.5 | 7.6 |
|  |  | 1000 | 7.0 | 7.4 | 7.4 | 7.6 | 7.6 | 7.4 |
|  |  | 2000 | 7.0 | 7.4 | 7.4 | 7.6 | 7.6 | 7.4 |
|  | 50 | 0 | 7.0 | 7.1 | 7.1 | 7.0 | 7.1 | 7.1 |
|  |  | 50 | 7.0 | 7.3 | 7.3 | 7.4 | 7.5 | 7.3 |
|  |  | 100 | 7.0 | 7.4 | 7.4 | 7.5 | 7.5 | 7.4 |
|  |  | 500 | 7.0 | 7.4 | 7.2 | 7.6 | 7.5 | 7.2 |
|  |  | 1000 | 7.0 | 7.4 | 7.2 | 7.5 | 7.5 | 7.2 |
|  |  | 2000 | 7.0 | 7.5 | 7.2 | 7.6 | 7.5 | 7.2 |
|  | 100 | 0 | 7.0 | 7.2 | 7.0 | 7.0 | 7.0 | 7.0 |
|  |  | 50 | 7.0 | 7.2 | 7.2 | 7.5 | 7.6 | 7.2 |
|  |  | 100 | 7.0 | 7.3 | 7.3 | 7.5 | 7.5 | 7.3 |
|  |  | 500 | 7.0 | 7.5 | 7.5 | 7.5 | 7.5 | 7.5 |
|  |  | 1000 | 7.0 | 7.3 | 7.4 | 7.5 | 7.6 | 7.4 |
|  |  | 2000 | 7.0 | 7.6 | 7.6 | 7.5 | 7.6 | 7.6 |
|  | 250 | 0 | 7.0 | 6.9 | 7.2 | 7.0 | 7.0 | 7.2 |
|  |  | 50 | 7.0 | 7.3 | 7.2 | 7.5 | 7.4 | 7.2 |
|  |  | 100 | 7.0 | 7.4 | 7.3 | 7.4 | 7.5 | 7.3 |
|  |  | 500 | 7.0 | 7.5 | 7.5 | 7.6 | 7.6 | 7.5 |
|  |  | 1000 | 7.0 | 7.4 | 7.3 | 7.6 | 7.5 | 7.3 |
|  |  | 2000 | 7.0 | 7.3 | 7.6 | 7.5 | 7.6 | 7.6 |
|  | 500 | 0 | 7.0 | 6.9 | 7.0 | 7.0 | 7.0 | 7.0 |
|  |  | 50 | 7.0 | 7.2 | 7.2 | 7.5 | 7.5 | 7.2 |
|  |  | 100 | 7.0 | 7.2 | 7.3 | 7.5 | 7.5 | 7.3 |
|  |  | 500 | 7.0 | 7.3 | 7.4 | 7.5 | 7.5 | 7.4 |
|  |  | 1000 | 7.0 | 7.3 | 7.5 | 7.5 | 7.5 | 7.5 |
|  |  | 2000 | 7.0 | 7.3 | 7.3 | 7.5 | 7.5 | 7.3 |
| *Bacillus* sp.isolate SB12 | 0 | 0 | 7.0 | 6.9 | 6.9 | 7.0 | 7.0 | 6.9 |
|  |  | 50 | 7.0 | 7.2 | 7.2 | 7.5 | 7.5 | 7.2 |
|  |  | 100 | 7.0 | 7.3 | 7.3 | 7.4 | 7.5 | 7.3 |
|  |  | 500 | 7.0 | 7.5 | 7.5 | 7.6 | 7.5 | 7.5 |
|  |  | 1000 | 7.0 | 7.3 | 7.3 | 7.6 | 7.5 | 7.3 |
|  |  | 2000 | 7.0 | 7.6 | 7.6 | 7.5 | 7.5 | 7.6 |
|  | 10 | 0 | 7.0 | 6.9 | 6.9 | 7.0 | 7.0 | 6.9 |
|  |  | 50 | 7.0 | 7.2 | 7.2 | 7.5 | 7.6 | 7.2 |
|  |  | 100 | 7.0 | 7.3 | 7.4 | 7.5 | 7.5 | 7.4 |
|  |  | 500 | 7.0 | 7.3 | 7.3 | 7.5 | 7.5 | 7.3 |
|  |  | 1000 | 7.0 | 7.3 | 7.3 | 7.5 | 7.6 | 7.3 |
|  |  | 2000 | 7.0 | 7.4 | 7.3 | 7.5 | 7.6 | 7.3 |
|  | 50 | 0 | 7.0 | 7.0 | 7.1 | 7.1 | 7.0 | 7.1 |
|  |  | 50 | 7.0 | 7.1 | 7.3 | 7.6 | 7.4 | 7.3 |
|  |  | 100 | 7.0 | 7.3 | 7.3 | 7.5 | 7.5 | 7.3 |
|  |  | 500 | 7.0 | 7.3 | 7.3 | 7.5 | 7.6 | 7.3 |
|  |  | 1000 | 7.0 | 7.5 | 7.3 | 7.6 | 7.5 | 7.3 |
|  |  | 2000 | 7.0 | 7.4 | 7.4 | 7.6 | 7.6 | 7.4 |
|  | 100 | 0 | 7.0 | 7.2 | 6.9 | 7.0 | 7.1 | 6.9 |
|  |  | 50 | 7.0 | 7.2 | 7.3 | 7.4 | 7.6 | 7.3 |
|  |  | 100 | 7.0 | 7.3 | 7.4 | 7.5 | 7.5 | 7.4 |
|  |  | 500 | 7.0 | 7.5 | 7.4 | 7.6 | 7.5 | 7.4 |
|  |  | 1000 | 7.0 | 7.3 | 7.3 | 7.5 | 7.6 | 7.3 |
|  |  | 2000 | 7.0 | 7.6 | 7.4 | 7.6 | 7.6 | 7.4 |
|  | 250 | 0 | 7.0 | 6.9 | 7.1 | 7.1 | 7.1 | 7.1 |
|  |  | 50 | 7.0 | 7.2 | 7.2 | 7.5 | 7.5 | 7.2 |
|  |  | 100 | 7.0 | 7.3 | 7.3 | 7.4 | 7.5 | 7.3 |
|  |  | 500 | 7.0 | 7.5 | 7.5 | 7.6 | 7.5 | 7.5 |
|  |  | 1000 | 7.0 | 7.3 | 7.3 | 7.6 | 7.5 | 7.3 |
|  |  | 2000 | 7.0 | 7.6 | 7.6 | 7.5 | 7.5 | 7.6 |
|  | 500 | 0 | 7.0 | 7.1 | 6.9 | 7.1 | 6.9 | 6.9 |
|  |  | 50 | 7.0 | 7.2 | 7.2 | 7.5 | 7.5 | 7.2 |
|  |  | 100 | 7.0 | 7.3 | 7.3 | 7.5 | 7.5 | 7.3 |
|  |  | 500 | 7.0 | 7.3 | 7.4 | 7.5 | 7.5 | 7.4 |
|  |  | 1000 | 7.0 | 7.4 | 7.5 | 7.5 | 7.5 | 7.5 |
|  |  | 2000 | 7.0 | 7.5 | 7.4 | 7.5 | 7.5 | 7.4 |
| *Bacillus* sp.isolate SB13 | 0 | 0 | 7.0 | 7.2 | 6.9 | 7.1 | 7.0 | 6.9 |
|  |  | 50 | 7.0 | 7.2 | 7.2 | 7.5 | 7.5 | 7.4 |
|  |  | 100 | 7.0 | 7.3 | 7.3 | 7.4 | 7.5 | 7.4 |
|  |  | 500 | 7.0 | 7.5 | 7.5 | 7.6 | 7.5 | 7.5 |
|  |  | 1000 | 7.0 | 7.3 | 7.3 | 7.6 | 7.5 | 7.4 |
|  |  | 2000 | 7.0 | 7.6 | 7.6 | 7.5 | 7.5 | 7.6 |
|  | 10 | 0 | 7.0 | 7.1 | 7.0 | 7.1 | 7.0 | 7.0 |
|  |  | 50 | 7.0 | 7.2 | 7.2 | 7.5 | 7.6 | 7.2 |
|  |  | 100 | 7.0 | 7.2 | 7.4 | 7.5 | 7.5 | 7.4 |
|  |  | 500 | 7.0 | 7.2 | 7.4 | 7.5 | 7.5 | 7.4 |
|  |  | 1000 | 7.0 | 7.3 | 7.3 | 7.5 | 7.6 | 7.3 |
|  |  | 2000 | 7.0 | 7.2 | 7.4 | 7.5 | 7.6 | 7.4 |
|  | 50 | 0 | 7.0 | 6.9 | 7.2 | 7.0 | 6.9 | 7.2 |
|  |  | 50 | 7.0 | 7.2 | 7.3 | 7.5 | 7.4 | 7.3 |
|  |  | 100 | 7.0 | 7.3 | 7.4 | 7.5 | 7.5 | 7.4 |
|  |  | 500 | 7.0 | 7.5 | 7.4 | 7.5 | 7.6 | 7.4 |
|  |  | 1000 | 7.0 | 7.3 | 7.4 | 7.5 | 7.5 | 7.4 |
|  |  | 2000 | 7.0 | 7.6 | 7.4 | 7.5 | 7.6 | 7.4 |
|  | 100 | 0 | 7.0 | 7.0 | 6.9 | 7.0 | 7.2 | 6.9 |
|  |  | 50 | 7.0 | 7.2 | 7.2 | 7.6 | 7.6 | 7.2 |
|  |  | 100 | 7.0 | 7.3 | 7.3 | 7.5 | 7.5 | 7.3 |
|  |  | 500 | 7.0 | 7.4 | 7.5 | 7.5 | 7.5 | 7.5 |
|  |  | 1000 | 7.0 | 7.1 | 7.3 | 7.6 | 7.6 | 7.3 |
|  |  | 2000 | 7.0 | 7.2 | 7.6 | 7.6 | 7.6 | 7.6 |
|  | 250 | 0 | 7.0 | 7.0 | 7.1 | 7.1 | 7.1 | 7.1 |
|  |  | 50 | 7.0 | 7.2 | 7.2 | 7.4 | 7.4 | 7.2 |
|  |  | 100 | 7.0 | 7.2 | 7.3 | 7.5 | 7.5 | 7.3 |
|  |  | 500 | 7.0 | 7.2 | 7.5 | 7.6 | 7.6 | 7.5 |
|  |  | 1000 | 7.0 | 7.2 | 7.3 | 7.5 | 7.5 | 7.3 |
|  |  | 2000 | 7.0 | 7.2 | 7.6 | 7.6 | 7.6 | 7.6 |
|  | 500 | 0 | 7.0 | 7.0 | 7.1 | 7.1 | 7.1 | 7.1 |
|  |  | 50 | 7.0 | 7.2 | 7.3 | 7.6 | 7.6 | 7.3 |
|  |  | 100 | 7.0 | 7.3 | 7.4 | 7.5 | 7.5 | 7.4 |
|  |  | 500 | 7.0 | 7.5 | 7.5 | 7.5 | 7.5 | 7.5 |
|  |  | 1000 | 7.0 | 7.3 | 7.4 | 7.6 | 7.6 | 7.4 |
|  |  | 2000 | 7.0 | 7.6 | 7.3 | 7.6 | 7.6 | 7.3 |
| *Bacillus* sp.isolate SB16 | 0 | 0 | 7.0 | 7.1 | 7.2 | 7.1 | 7.0 | 7.2 |
|  |  | 50 | 7.0 | 7.2 | 7.2 | 7.4 | 7.5 | 7.2 |
|  |  | 100 | 7.0 | 7.3 | 7.3 | 7.5 | 7.5 | 7.3 |
|  |  | 500 | 7.0 | 7.3 | 7.5 | 7.6 | 7.5 | 7.5 |
|  |  | 1000 | 7.0 | 7.3 | 7.3 | 7.5 | 7.5 | 7.3 |
|  |  | 2000 | 7.0 | 7.2 | 7.6 | 7.6 | 7.5 | 7.6 |
|  | 10 | 0 | 7.0 | 7.0 | 7.1 | 7.0 | 6.9 | 7.1 |
|  |  | 50 | 7.0 | 7.3 | 7.2 | 7.6 | 7.6 | 7.2 |
|  |  | 100 | 7.0 | 7.5 | 7.2 | 7.5 | 7.5 | 7.2 |
|  |  | 500 | 7.0 | 7.6 | 7.2 | 7.5 | 7.5 | 7.2 |
|  |  | 1000 | 7.0 | 7.4 | 7.3 | 7.6 | 7.6 | 7.3 |
|  |  | 2000 | 7.0 | 7.4 | 7.2 | 7.6 | 7.6 | 7.2 |
|  | 50 | 0 | 7.0 | 7.1 | 6.9 | 7.1 | 7.1 | 6.9 |
|  |  | 50 | 7.0 | 7.3 | 7.2 | 7.4 | 7.5 | 7.2 |
|  |  | 100 | 7.0 | 7.4 | 7.3 | 7.5 | 7.5 | 7.3 |
|  |  | 500 | 7.0 | 7.2 | 7.5 | 7.6 | 7.5 | 7.5 |
|  |  | 1000 | 7.0 | 7.2 | 7.3 | 7.5 | 7.5 | 7.3 |
|  |  | 2000 | 7.0 | 7.2 | 7.6 | 7.6 | 7.5 | 7.6 |
|  | 100 | 0 | 7.0 | 7.0 | 7.0 | 7.1 | 7.0 | 7.0 |
|  |  | 50 | 7.0 | 7.2 | 7.2 | 7.6 | 7.6 | 7.2 |
|  |  | 100 | 7.0 | 7.3 | 7.3 | 7.5 | 7.5 | 7.3 |
|  |  | 500 | 7.0 | 7.5 | 7.4 | 7.5 | 7.5 | 7.4 |
|  |  | 1000 | 7.0 | 7.4 | 7.1 | 7.6 | 7.6 | 7.1 |
|  |  | 2000 | 7.0 | 7.6 | 7.2 | 7.6 | 7.6 | 7.2 |
|  | 250 | 0 | 7.0 | 7.2 | 7.0 | 7.1 | 7.0 | 7.0 |
|  |  | 50 | 7.0 | 7.2 | 7.2 | 7.4 | 7.4 | 7.2 |
|  |  | 100 | 7.0 | 7.3 | 7.2 | 7.5 | 7.5 | 7.2 |
|  |  | 500 | 7.0 | 7.5 | 7.2 | 7.6 | 7.6 | 7.2 |
|  |  | 1000 | 7.0 | 7.3 | 7.2 | 7.5 | 7.5 | 7.2 |
|  |  | 2000 | 7.0 | 7.6 | 7.2 | 7.6 | 7.6 | 7.2 |
|  | 500 | 0 | 7.0 | 7.0 | 7.0 | 7.1 | 7.0 | 7.0 |
|  |  | 50 | 7.0 | 7.2 | 7.2 | 7.5 | 7.5 | 7.2 |
|  |  | 100 | 7.0 | 7.3 | 7.3 | 7.5 | 7.5 | 7.3 |
|  |  | 500 | 7.0 | 7.4 | 7.5 | 7.5 | 7.5 | 7.5 |
|  |  | 1000 | 7.0 | 7.5 | 7.3 | 7.5 | 7.5 | 7.3 |
|  |  | 2000 | 7.0 | 7.3 | 7.6 | 7.5 | 7.5 | 7.6 |
| *Bacillus* sp.isolate SW3 | 0 | 0 | 7.0 | 7.23 | 7.1 | 7.1 | 7.0 | 6.9 |
|  |  | 50 | 7.0 | 7.2 | 7.2 | 7.6 | 7.5 | 7.2 |
|  |  | 100 | 7.0 | 7.3 | 7.3 | 7.5 | 7.5 | 7.3 |
|  |  | 500 | 7.0 | 7.5 | 7.3 | 7.5 | 7.5 | 7.5 |
|  |  | 1000 | 7.0 | 7.3 | 7.3 | 7.6 | 7.5 | 7.3 |
|  |  | 2000 | 7.0 | 7.6 | 7.2 | 7.6 | 7.5 | 7.6 |
|  | 10 | 0 | 7.0 | 7.1 | 7.0 | 7.1 | 7.0 | 6.9 |
|  |  | 50 | 7.0 | 7.2 | 7.3 | 7.4 | 7.6 | 7.2 |
|  |  | 100 | 7.0 | 7.2 | 7.5 | 7.5 | 7.5 | 7.4 |
|  |  | 500 | 7.0 | 7.2 | 7.6 | 7.6 | 7.5 | 7.3 |
|  |  | 1000 | 7.0 | 7.3 | 7.4 | 7.5 | 7.6 | 7.3 |
|  |  | 2000 | 7.0 | 7.2 | 7.4 | 7.6 | 7.6 | 7.3 |
|  | 50 | 0 | 7.0 | 6.9 | 7.1 | 7.1 | 7.0 | 7.1 |
|  |  | 50 | 7.0 | 7.2 | 7.3 | 7.5 | 7.4 | 7.3 |
|  |  | 100 | 7.0 | 7.3 | 7.4 | 7.4 | 7.5 | 7.3 |
|  |  | 500 | 7.0 | 7.5 | 7.2 | 7.6 | 7.6 | 7.3 |
|  |  | 1000 | 7.0 | 7.3 | 7.2 | 7.6 | 7.5 | 7.3 |
|  |  | 2000 | 7.0 | 7.6 | 7.2 | 7.5 | 7.6 | 7.4 |
|  | 100 | 0 | 7.0 | 7.0 | 7.0 | 7.0 | 7.1 | 6.9 |
|  |  | 50 | 7.0 | 7.2 | 7.2 | 7.4 | 7.6 | 7.3 |
|  |  | 100 | 7.0 | 7.3 | 7.3 | 7.5 | 7.5 | 7.4 |
|  |  | 500 | 7.0 | 7.4 | 7.5 | 7.6 | 7.5 | 7.4 |
|  |  | 1000 | 7.0 | 7.1 | 7.4 | 7.5 | 7.6 | 7.3 |
|  |  | 2000 | 7.0 | 7.2 | 7.6 | 7.5 | 7.6 | 7.4 |
|  | 250 | 0 | 7.0 | 7.0 | 7.2 | 7.2 | 7.1 | 7.1 |
|  |  | 50 | 7.0 | 7.2 | 7.2 | 7.5 | 7.5 | 7.2 |
|  |  | 100 | 7.0 | 7.2 | 7.3 | 7.5 | 7.5 | 7.3 |
|  |  | 500 | 7.0 | 7.2 | 7.5 | 7.5 | 7.5 | 7.5 |
|  |  | 1000 | 7.0 | 7.2 | 7.3 | 7.5 | 7.5 | 7.3 |
|  |  | 2000 | 7.0 | 7.2 | 7.6 | 7.5 | 7.5 | 7.6 |
|  | 500 | 0 | 7.0 | 7.0 | 7.0 | 7.0 | 6.9 | 6.9 |
|  |  | 50 | 7.0 | 7.2 | 7.2 | 7.5 | 7.5 | 7.2 |
|  |  | 100 | 7.0 | 7.3 | 7.3 | 7.4 | 7.5 | 7.3 |
|  |  | 500 | 7.0 | 7.5 | 7.4 | 7.6 | 7.5 | 7.4 |
|  |  | 1000 | 7.0 | 7.3 | 7.5 | 7.6 | 7.5 | 7.5 |
|  |  | 2000 | 7.0 | 7.6 | 7.3 | 7.5 | 7.5 | 7.4 |
| *Bacillus* sp.isolate SW7 | 0 | 0 | 7.0 | 7.1 | 7.2 | 7.2 | 7.0 | 6.9 |
|  |  | 50 | 7.0 | 7.2 | 7.2 | 7.5 | 7.5 | 7.2 |
|  |  | 100 | 7.0 | 7.3 | 7.3 | 7.5 | 7.5 | 7.3 |
|  |  | 500 | 7.0 | 7.3 | 7.5 | 7.5 | 7.5 | 7.5 |
|  |  | 1000 | 7.0 | 7.3 | 7.3 | 7.5 | 7.5 | 7.3 |
|  |  | 2000 | 7.0 | 7.2 | 7.6 | 7.5 | 7.5 | 7.6 |
|  | 10 | 0 | 7.0 | 7.0 | 7.1 | 7.1 | 7.0 | 7.0 |
|  |  | 50 | 7.0 | 7.3 | 7.2 | 7.6 | 7.6 | 7.2 |
|  |  | 100 | 7.0 | 7.5 | 7.2 | 7.5 | 7.5 | 7.4 |
|  |  | 500 | 7.0 | 7.6 | 7.2 | 7.5 | 7.5 | 7.4 |
|  |  | 1000 | 7.0 | 7.4 | 7.3 | 7.6 | 7.6 | 7.3 |
|  |  | 2000 | 7.0 | 7.4 | 7.2 | 7.6 | 7.6 | 7.4 |
|  | 50 | 0 | 7.0 | 7.1 | 6.9 | 7.0 | 6.9 | 7.2 |
|  |  | 50 | 7.0 | 7.3 | 7.2 | 7.4 | 7.4 | 7.3 |
|  |  | 100 | 7.0 | 7.4 | 7.3 | 7.5 | 7.5 | 7.4 |
|  |  | 500 | 7.0 | 7.2 | 7.5 | 7.6 | 7.6 | 7.4 |
|  |  | 1000 | 7.0 | 7.2 | 7.3 | 7.5 | 7.5 | 7.4 |
|  |  | 2000 | 7.0 | 7.2 | 7.6 | 7.6 | 7.6 | 7.4 |
|  | 100 | 0 | 7.0 | 7.0 | 7.0 | 7.0 | 7.2 | 6.9 |
|  |  | 50 | 7.0 | 7.2 | 7.2 | 7.5 | 7.6 | 7.2 |
|  |  | 100 | 7.0 | 7.3 | 7.3 | 7.4 | 7.5 | 7.3 |
|  |  | 500 | 7.0 | 7.5 | 7.4 | 7.6 | 7.5 | 7.5 |
|  |  | 1000 | 7.0 | 7.4 | 7.1 | 7.6 | 7.6 | 7.3 |
|  |  | 2000 | 7.0 | 7.6 | 7.2 | 7.5 | 7.6 | 7.6 |
|  | 250 | 0 | 7.0 | 7.2 | 7.0 | 7.0 | 7.1 | 7.1 |
|  |  | 50 | 7.0 | 7.2 | 7.2 | 7.5 | 7.4 | 7.2 |
|  |  | 100 | 7.0 | 7.3 | 7.2 | 7.5 | 7.5 | 7.3 |
|  |  | 500 | 7.0 | 7.5 | 7.2 | 7.5 | 7.6 | 7.5 |
|  |  | 1000 | 7.0 | 7.3 | 7.2 | 7.5 | 7.5 | 7.3 |
|  |  | 2000 | 7.0 | 7.6 | 7.2 | 7.5 | 7.6 | 7.6 |
|  | 500 | 0 | 7.0 | 7.0 | 7.0 | 7.0 | 7.1 | 7.1 |
|  |  | 50 | 7.0 | 7.2 | 7.2 | 7.5 | 7.6 | 7.3 |
|  |  | 100 | 7.0 | 7.3 | 7.3 | 7.4 | 7.5 | 7.4 |
|  |  | 500 | 7.0 | 7.4 | 7.5 | 7.6 | 7.5 | 7.5 |
|  |  | 1000 | 7.0 | 7.5 | 7.3 | 7.6 | 7.6 | 7.4 |
|  |  | 2000 | 7.0 | 7.3 | 7.6 | 7.5 | 7.6 | 7.3 |
| *Escherichia* sp.isolate SW11 | 0 | 0 | 7.0 | 7.23 | 7.1 | 7.0 | 7.0 | 6.9 |
|  |  | 50 | 7.0 | 7.2 | 7.2 | 7.5 | 7.4 | 7.4 |
|  |  | 100 | 7.0 | 7.3 | 7.3 | 7.5 | 7.5 | 7.4 |
|  |  | 500 | 7.0 | 7.5 | 7.3 | 7.5 | 7.6 | 7.5 |
|  |  | 1000 | 7.0 | 7.3 | 7.3 | 7.5 | 7.5 | 7.4 |
|  |  | 2000 | 7.0 | 7.6 | 7.2 | 7.5 | 7.6 | 7.6 |
|  | 10 | 0 | 7.0 | 7.1 | 7.0 | 7.0 | 7.0 | 7.0 |
|  |  | 50 | 7.0 | 7.2 | 7.3 | 7.6 | 7.5 | 7.2 |
|  |  | 100 | 7.0 | 7.2 | 7.5 | 7.5 | 7.5 | 7.4 |
|  |  | 500 | 7.0 | 7.2 | 7.6 | 7.5 | 7.5 | 7.4 |
|  |  | 1000 | 7.0 | 7.3 | 7.4 | 7.6 | 7.5 | 7.3 |
|  |  | 2000 | 7.0 | 7.2 | 7.4 | 7.6 | 7.5 | 7.4 |
|  | 50 | 0 | 7.0 | 6.9 | 7.1 | 7.1 | 7.0 | 7.2 |
|  |  | 50 | 7.0 | 7.2 | 7.3 | 7.4 | 7.5 | 7.3 |
|  |  | 100 | 7.0 | 7.3 | 7.4 | 7.5 | 7.5 | 7.4 |
|  |  | 500 | 7.0 | 7.5 | 7.2 | 7.6 | 7.5 | 7.4 |
|  |  | 1000 | 7.0 | 7.3 | 7.2 | 7.5 | 7.5 | 7.4 |
|  |  | 2000 | 7.0 | 7.6 | 7.2 | 7.6 | 7.5 | 7.4 |
|  | 100 | 0 | 7.0 | 7.0 | 7.0 | 7.0 | 7.0 | 6.9 |
|  |  | 50 | 7.0 | 7.2 | 7.2 | 7.5 | 7.5 | 7.2 |
|  |  | 100 | 7.0 | 7.3 | 7.3 | 7.4 | 7.5 | 7.3 |
|  |  | 500 | 7.0 | 7.4 | 7.5 | 7.6 | 7.5 | 7.5 |
|  |  | 1000 | 7.0 | 7.1 | 7.4 | 7.6 | 7.5 | 7.3 |
|  |  | 2000 | 7.0 | 7.2 | 7.6 | 7.5 | 7.5 | 7.6 |
|  | 250 | 0 | 7.0 | 7.0 | 7.2 | 7.1 | 7.1 | 7.1 |
|  |  | 50 | 7.0 | 7.2 | 7.2 | 7.5 | 7.5 | 7.2 |
|  |  | 100 | 7.0 | 7.2 | 7.3 | 7.5 | 7.5 | 7.3 |
|  |  | 500 | 7.0 | 7.2 | 7.5 | 7.5 | 7.5 | 7.5 |
|  |  | 1000 | 7.0 | 7.2 | 7.3 | 7.5 | 7.5 | 7.3 |
|  |  | 2000 | 7.0 | 7.2 | 7.6 | 7.5 | 7.5 | 7.6 |
|  | 500 | 0 | 7.0 | 7.0 | 7.0 | 7.0 | 7.1 | 7.1 |
|  |  | 50 | 7.0 | 7.2 | 7.2 | 7.5 | 7.5 | 7.3 |
|  |  | 100 | 7.0 | 7.3 | 7.3 | 7.4 | 7.5 | 7.4 |
|  |  | 500 | 7.0 | 7.5 | 7.4 | 7.6 | 7.5 | 7.5 |
|  |  | 1000 | 7.0 | 7.3 | 7.5 | 7.6 | 7.5 | 7.4 |
|  |  | 2000 | 7.0 | 7.6 | 7.3 | 7.5 | 7.5 | 7.3 |
| Consortium | 0 | 0 | 7.0 | 7.1 | 6.9 | 7.0 | 7.2 | 7.2 |
|  |  | 50 | 7.0 | 7.2 | 7.2 | 7.5 | 7.5 | 7.2 |
|  |  | 100 | 7.0 | 7.3 | 7.3 | 7.5 | 7.5 | 7.3 |
|  |  | 500 | 7.0 | 7.3 | 7.5 | 7.5 | 7.5 | 7.5 |
|  |  | 1000 | 7.0 | 7.3 | 7.3 | 7.5 | 7.5 | 7.3 |
|  |  | 2000 | 7.0 | 7.2 | 7.6 | 7.5 | 7.5 | 7.6 |
|  | 10 | 0 | 7.0 | 7.0 | 6.9 | 7.0 | 7.1 | 7.1 |
|  |  | 50 | 7.0 | 7.3 | 7.2 | 7.6 | 7.6 | 7.2 |
|  |  | 100 | 7.0 | 7.5 | 7.4 | 7.5 | 7.5 | 7.2 |
|  |  | 500 | 7.0 | 7.6 | 7.3 | 7.5 | 7.5 | 7.2 |
|  |  | 1000 | 7.0 | 7.4 | 7.3 | 7.6 | 7.6 | 7.3 |
|  |  | 2000 | 7.0 | 7.4 | 7.3 | 7.6 | 7.6 | 7.2 |
|  | 50 | 0 | 7.0 | 7.1 | 7.1 | 7.1 | 6.9 | 6.9 |
|  |  | 50 | 7.0 | 7.3 | 7.3 | 7.4 | 7.4 | 7.2 |
|  |  | 100 | 7.0 | 7.4 | 7.3 | 7.5 | 7.5 | 7.3 |
|  |  | 500 | 7.0 | 7.2 | 7.3 | 7.6 | 7.6 | 7.5 |
|  |  | 1000 | 7.0 | 7.2 | 7.3 | 7.5 | 7.5 | 7.3 |
|  |  | 2000 | 7.0 | 7.2 | 7.4 | 7.6 | 7.6 | 7.6 |
|  | 100 | 0 | 7.0 | 7.0 | 6.9 | 6.9 | 7.0 | 7.0 |
|  |  | 50 | 7.0 | 7.2 | 7.3 | 7.5 | 7.5 | 7.2 |
|  |  | 100 | 7.0 | 7.3 | 7.4 | 7.5 | 7.4 | 7.3 |
|  |  | 500 | 7.0 | 7.5 | 7.4 | 7.5 | 7.6 | 7.4 |
|  |  | 1000 | 7.0 | 7.4 | 7.3 | 7.5 | 7.6 | 7.1 |
|  |  | 2000 | 7.0 | 7.6 | 7.4 | 7.5 | 7.5 | 7.2 |
|  | 250 | 0 | 7.0 | 7.2 | 7.1 | 7.1 | 7.0 | 7.0 |
|  |  | 50 | 7.0 | 7.2 | 7.2 | 7.5 | 7.6 | 7.2 |
|  |  | 100 | 7.0 | 7.3 | 7.3 | 7.4 | 7.5 | 7.2 |
|  |  | 500 | 7.0 | 7.5 | 7.5 | 7.6 | 7.5 | 7.2 |
|  |  | 1000 | 7.0 | 7.3 | 7.3 | 7.6 | 7.6 | 7.2 |
|  |  | 2000 | 7.0 | 7.6 | 7.6 | 7.5 | 7.6 | 7.2 |
|  | 500 | 0 | 7.0 | 7.0 | 6.9 | 7.0 | 7.0 | 7.0 |
|  |  | 50 | 7.0 | 7.2 | 7.2 | 7.5 | 7.4 | 7.2 |
|  |  | 100 | 7.0 | 7.3 | 7.3 | 7.5 | 7.5 | 7.3 |
|  |  | 500 | 7.0 | 7.4 | 7.4 | 7.5 | 7.6 | 7.5 |
|  |  | 1000 | 7.0 | 7.5 | 7.5 | 7.5 | 7.5 | 7.3 |
|  |  | 2000 | 7.0 | 7.3 | 7.4 | 7.5 | 7.6 | 7.6 |

**Supplementary Figure S1. SEM images of the 13 DPAO isolates.**

**
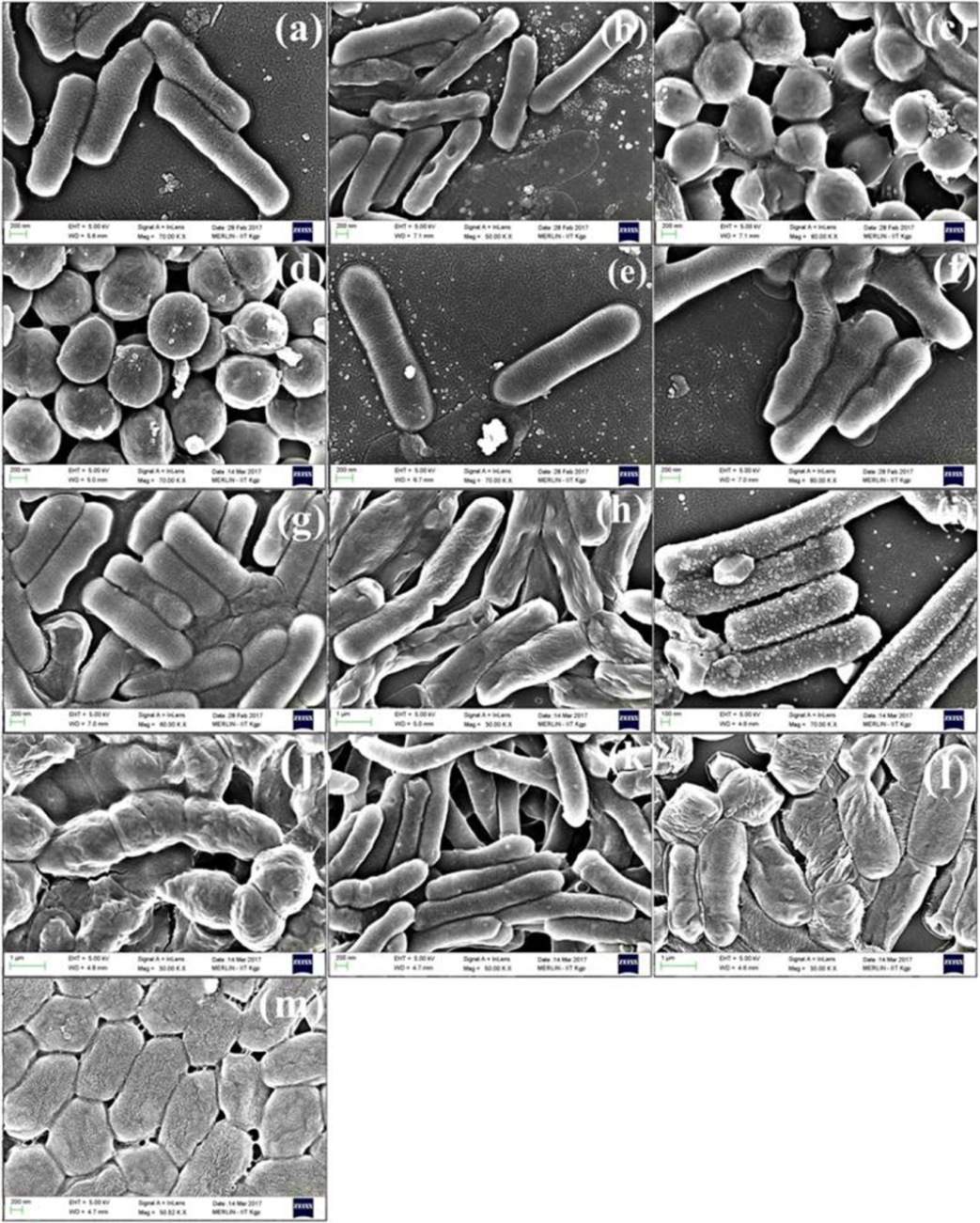
**

(a) Isolate H1 *Bacillus* sp. KU740213-KU740214; (b) Isolate H2 *Bacillus* sp. KU740215-KU740216; (c) Isolate H3 *Staphylococcus* sp. KU740217-KU740218; (d) Isolate H4 *Staphylococcus* sp. KU740219-KU740220; (e) Isolate H9 *Bacillus* sp. KU740221-KU740222; (f) Isolate SB3 *Bacillus* sp. KU740223-KU740224; (g) Isolate SB6 *Bacillus* sp. KU740225-KU740226; (h) Isolate SB12 *Bacillus* sp. KU740227-KU740228; (i) Isolate SB13 *Bacillus* sp. KU740229-KU740230; (j) Isolate SB16 *Bacillus* sp. KU740231-KU740232; (k) Isolate SW3 *Bacillus* sp. KU740233-KU740234; (l) Isolate SW7 *Bacillus* sp. KU740235-KU740236; (m) Isolate SW11 *Escherichia* sp. KU740237-KU740238.

All the images are at the same magnification. Each of the DPAO isolates is observed to show distinct morphology differing from each other in shape and size.

**Supplementary Figure S2. Gram characters and biochemical characteristics of the bacterial isolates.**

Indole production test- IT; Methyl Red test- MRT; Voges-Proskaur test- VPT; Citrate Utilization test- CUT; Oxidase test- OT; Catalase test- CT; Lactose Fermentation test- LFT; MacConkey Agar- MCA; Lactose Fermenter- LF; Non-Lactose Fermenter- NLF; Urease Production test- UPT; Nitrate Reduction test- NRT; Starch Hydrolysis test- SHT; Esculin Hydrolysis test- EHT; Bile Utilization test- BU; Esculin Utilization test- EU; Cysteine Desulfurase test- CDT; Triple Sugar Iron test- TSIT; Phenylalanine Deaminase test- PDT; Sugar Fermentation and Gas Production test with the following sugars: Glucose- Glu, Mannitol- Man, Xylose- Xyl, Lactose- Lac, Maltose- Mal, Sucrose- Suc, Fructose- Fru, Galactose- Gal, Inositol- Ino, Cellobiose- Cel; Sugar Utilization- SU; Gas Production- GP. K/A denotes catabolism of peptone and fermentation of only glucose; A/A denotes fermentation of glucose and lactose and/or sucrose; NC/NC denotes no fermentation. ‘+’ denotes positive result and ‘–’ denotes negative result.

**Supplementary Figure S3.** **Percentage of P removal in 96 hours by the DPAO consortium from SW.**

**
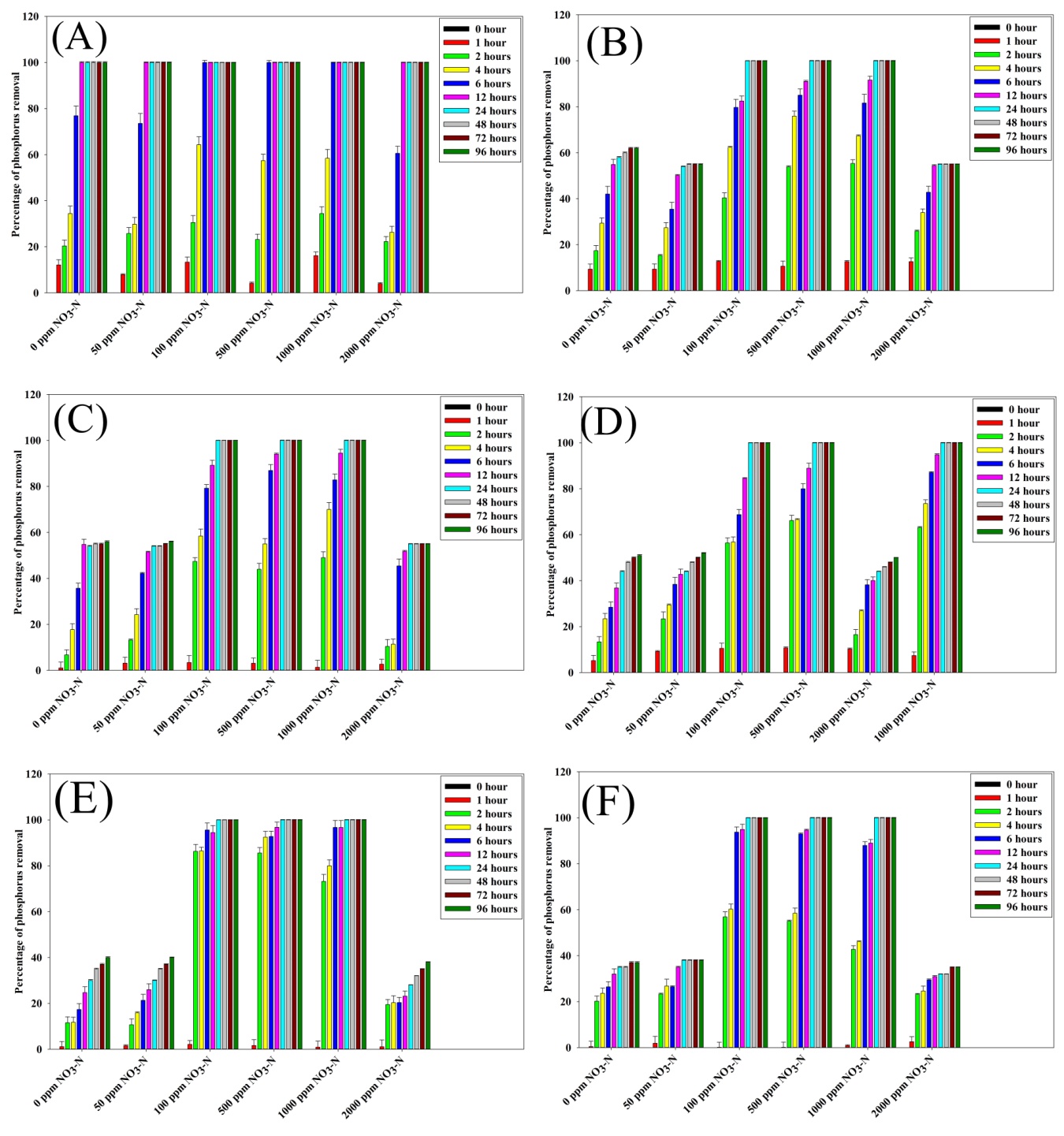
**

Percentage of P removal in 96 hours by DPAO consortium from SW in the presence of 0-2000 ppm of NO_3_^-^-N where, (A) 10 ppm P conc., (B) 50 ppm P conc., (C) 100 ppm P conc., (D) 250 ppm P conc., (E) 500 ppm P conc., (F) 1000 ppm P conc.

**Supplementary Figure S4. Percentage of P removal from SW in 96 hours by the control non-PAO, *Escherichia coli* K12 ER2925.**


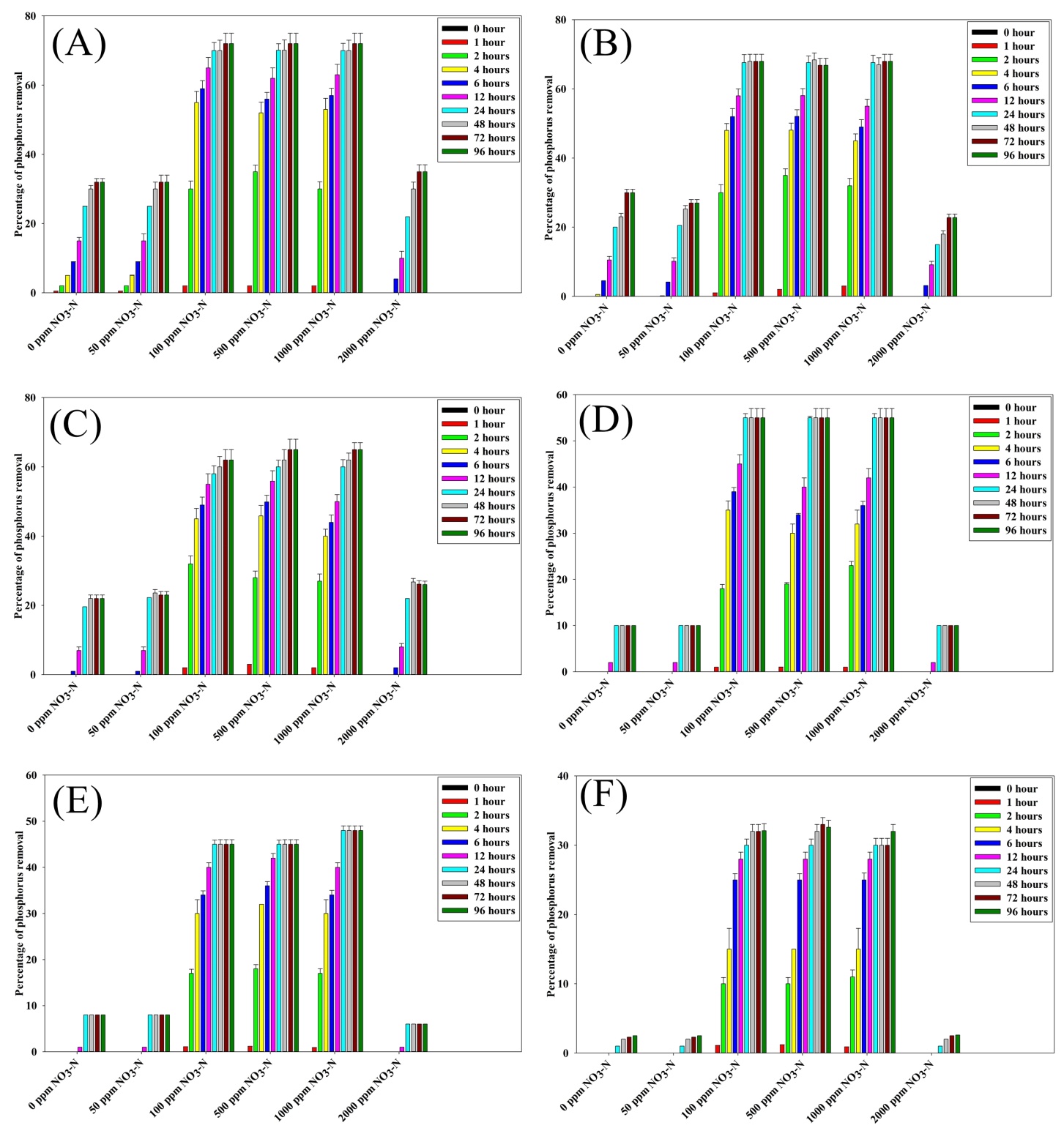


Percentage of P removal in 96 hours by control non-PAO *Escherichia coli* K12 ER2925 from SW in the presence of 0-2000 ppm of NO_3_-N where, (A) 10 ppm P conc., (B) 50 ppm P conc., (C) 100 ppm P conc., (D) 250 ppm P conc., (E) 500 ppm P conc., (F) 1000 ppm P conc.

**Supplementary Figure S5. Growth curve of the different bacterial isolates over 96 hrs.**


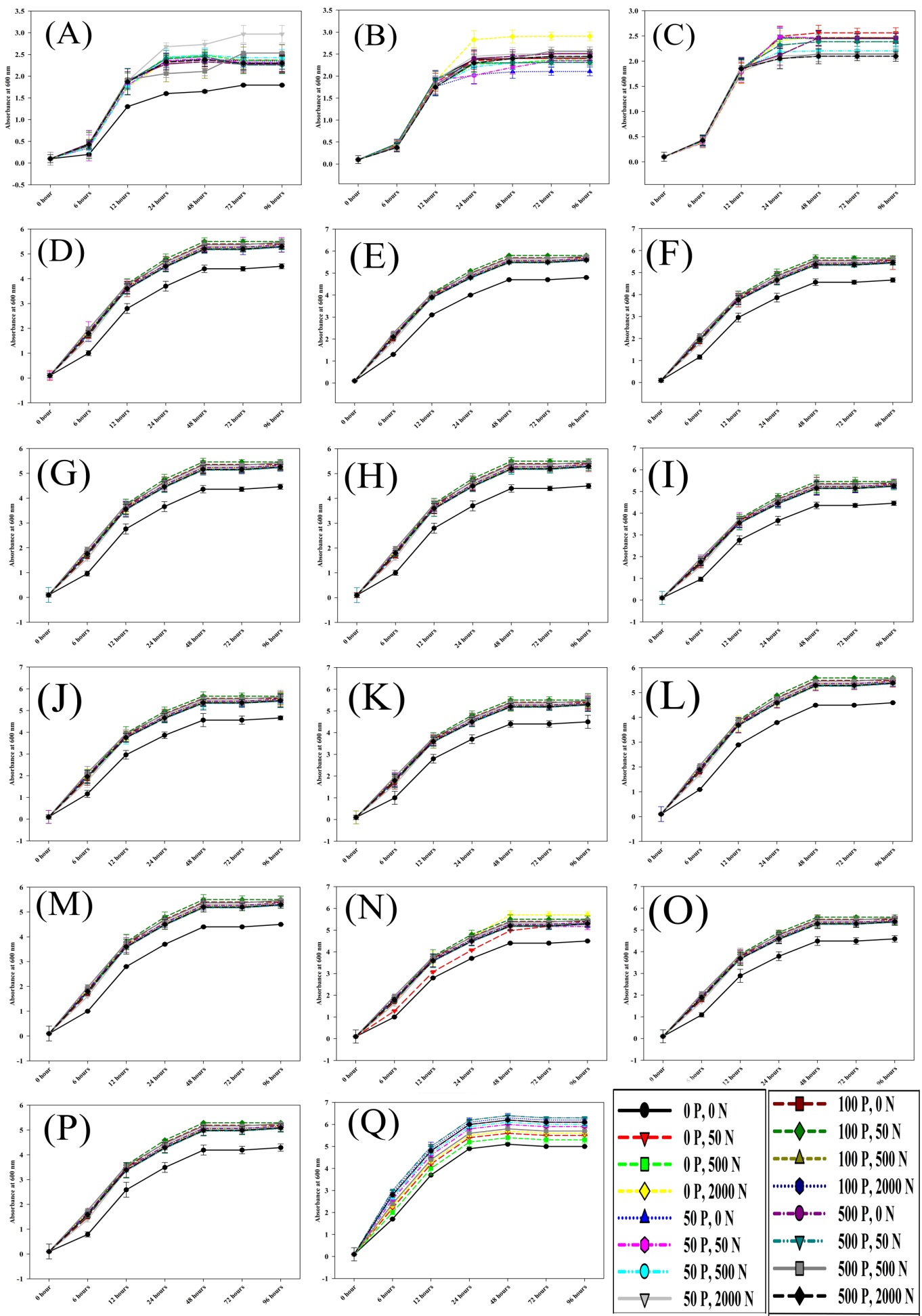


(A) *Escherichia coli* K12 ER2925, (B) *Escherichia coli* K12 PR1031, (C) *Escherichia coli* DH5α, (D) *Bacillus* sp. Isolate H1; (E) *Bacillus* sp. Isolate H2; (F) *Staphylococcus* sp. Isolate H3; (G) *Staphylococcus* sp. Isolate H4; (H) *Bacillus* sp. Isolate H9; (I) *Bacillus* sp. Isolate SB3; (J) *Bacillus* sp. Isolate SB6; (K) *Bacillus* sp. Isolate SB12; (L) *Bacillus* sp. Isolate SB13; (M) *Bacillus* sp. Isolate SB16; (N) *Bacillus* sp. Isolate SW3; (O) *Bacillus* sp. Isolate SW7; (P) *Escherichia* sp. Isolate SW11, (Q) DPAO Consortium.

**Supplementary Figure S6. NO_3_^-^-N removal percentages from the mixed effluent medium by the DPAO consortium.**

**
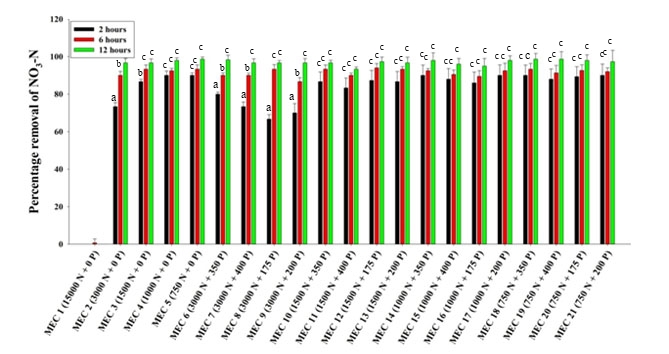
**

Percentages of NO_3_^-^-N removal over 12 hr from the mixed effluent medium with different combinations of PO_4_^3-^-P and NO_3_^-^-N conc. obtained by mixing stillage from a rice-based distillery industry and effluent from an explosive industry. In all the combinations, ~100% NO_3_^-^-N removal was achieved in 12 hr except in MEC 1 which has 15000 ppm NO_3_^-^-N conc. MEC: Mixed effluent combinations.

**Supplementary Figure S7. Chemical kinetics of PO_4_^3-^-P and NO_3_^-^-N** **removal by the DPAO consortium.**


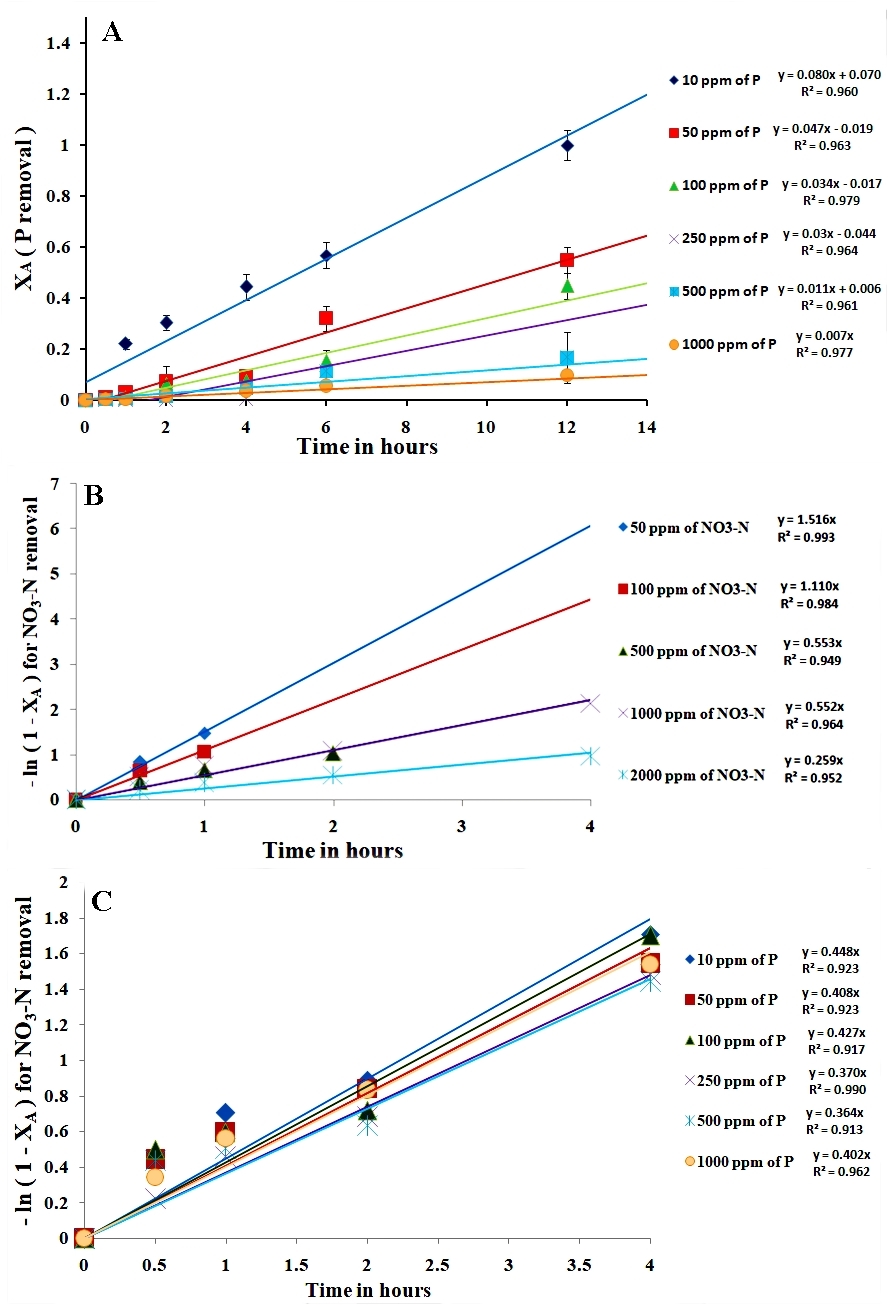


Determination of the rate of PO_4_^3-^-P and NO_3_^-^-N removal by the DPAO consortium. (A) Zero-order kinetics were followed for PO_4_^3-^-P removal within 12 hr in the absence of NO_3_^-^-N. First-order kinetics were followed for NO_3_^-^-N removal within 4 hr (B) in the absence of PO_4_^3-^-P, and (C) in presence of 10-1000 ppm of PO_4_^3-^-P.

**Supplementary Figure S8. The M value of the different reference genes.**

**
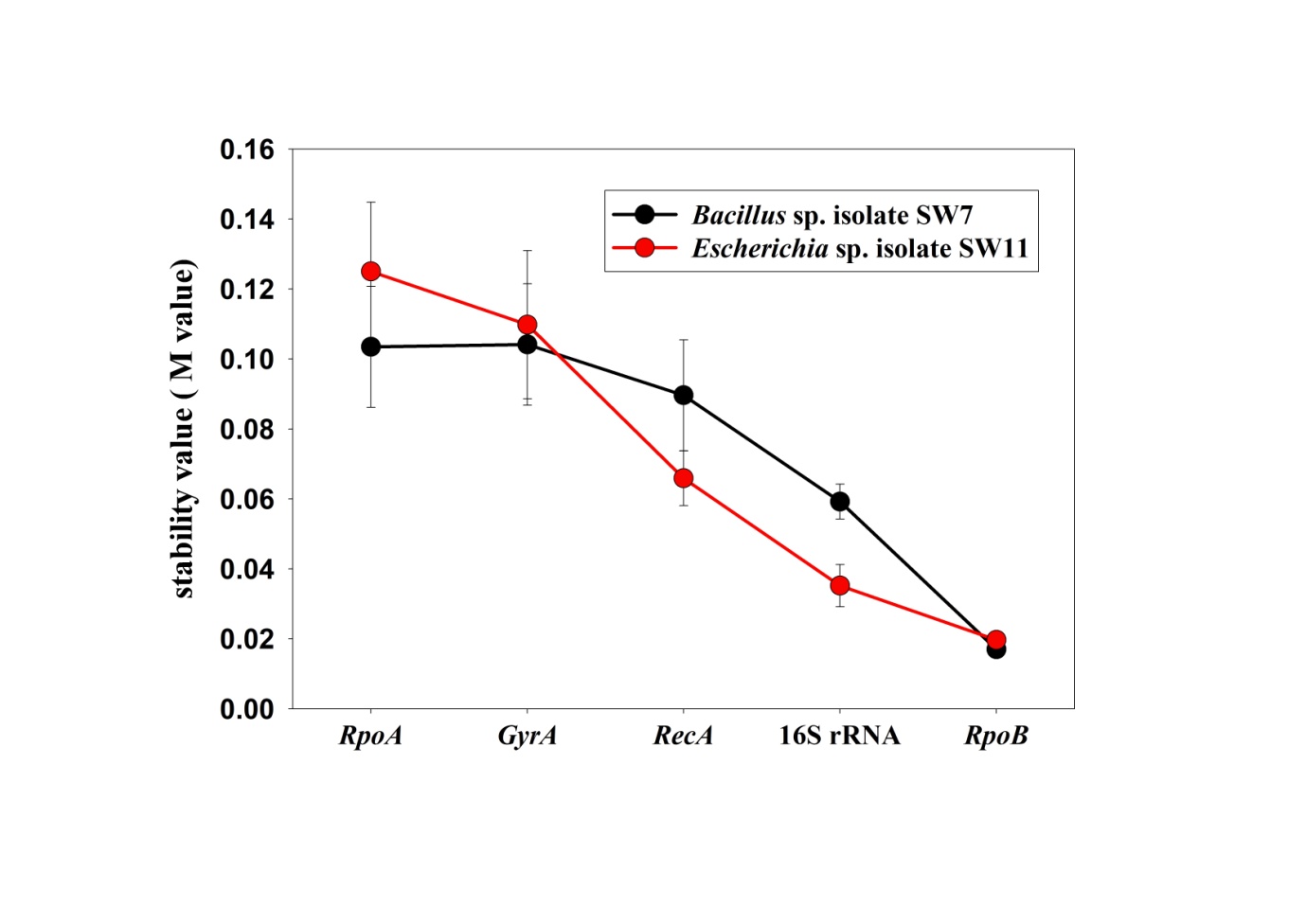
**

The M values of the different reference genes were determined initially. The gene, *RpoB*, showed the least M value for both the bacterial isolates, *Bacillus* sp. isolate SW7 and *Escherichia coli* isolate SW11. Hence, this gene was used as the reference gene in the further experiments of real time PCR.
